# Quality of Surgical Outcome Reporting in Randomised Clinical Trials of Multimodal Rectal Cancer Treatment: A Systematic Review

**DOI:** 10.3390/cancers16010026

**Published:** 2023-12-20

**Authors:** Joanna Janczak, Kristjan Ukegjini, Stephan Bischofberger, Matthias Turina, Philip C. Müller, Thomas Steffen

**Affiliations:** 1 Clinic for General and Visceral Surgery, Hospital for the Region Fürstenland Toggenburg, CH-9500 Wil, Switzerland; joanna.janczak@srft.ch; 2 Department of Surgery, Hospital of the Canton of St. Gallen, CH-9007 St. Gallen, Switzerland; kristjan.ukegjini@kssg.ch (K.U.); stephan.bischofberger@kssg.ch (S.B.); 3 Department of Surgery and Transplantation, University Hospital Zurich, CH-8091 Zurich, Switzerland; matthias.turina@usz.ch; 4 Department of Surgery, Clarunis—University Centre for Gastrointestinal and Hepatopancreatobiliary Diseases, CH-4002 Basel, Switzerland; philip.mueller@clarunis.ch

**Keywords:** rectal cancer, radiotherapy, chemotherapy, randomised controlled trials, surgical quality

## Abstract

**Simple Summary:**

Reporting surgical outcome and complication data in RCTs on rectal cancer is important because it is the basis for judging whether the results of a study warrant a change in clinical practice. In this review, we systematically analysed the quality of reporting in RCTs and the completeness rate of reporting of surgical outcomes and complication data. We found that only 2% (N = 7) of the RCTs met all 14 reporting criteria, and nearly half (N = 168, 49%) completed the procedure-specific quality criteria noted in the article. The most underreported criteria included complication severity (15% of articles).

**Abstract:**

Introduction: Randomised controlled trials (RCTs) continue to provide the best evidence for treatment options, but the quality of reporting in RCTs and the completeness rate of reporting of surgical outcomes and complication data vary widely. The aim of this study was to measure the quality of reporting of the surgical outcome and complication data in RCTs of rectal cancer treatment and whether this quality has changed over time. Methods: Eligible articles with the keywords (“rectal cancer” OR “rectal carcinoma”) AND (“radiation” OR “radiotherapy”) that were RCTs and published in the English, German, Polish, or Italian language were identified by reviewing all abstracts published from 1982 through 2022. Two authors independently screened and analysed all studies. The quality of the surgical outcome and complication data was assessed based on fourteen criteria, and the quality of RCTs was evaluated based on a modified Jadad scale. The primary outcome was the quality of reporting in RCTs and the completeness rate of reporting of surgical results and complication data. Results: A total of 340 articles reporting multimodal therapy outcomes for 143,576 rectal cancer patients were analysed. A total of 7 articles (2%) met all 14 reporting criteria, 13 met 13 criteria, 27 met from 11 to 12 criteria, 36 met from 9 to 10 criteria, 76 met from 7 to 8 criteria, and most articles met fewer than 7 criteria (mean 5.5 criteria). Commonly underreported criteria included complication severity (15% of articles), macroscopic integrity of mesorectal excision (17% of articles), length of stay (18% of articles), number of lymph nodes (21% of articles), distance between the tumour and circumferential resection margin (CRM) (26% of articles), surgical radicality according to the site of the primary tumour (R0 vs. R1 + R2) (29% of articles), and CRM status (38% of articles). Conclusion: Inconsistent surgical outcome and complication data reporting in multimodal rectal cancer treatment RCTs is standard. Standardised reporting of clinical and oncological outcomes should be established to facilitate comparing studies and results of related research topics.

## 1. Introduction

Only little is known about the quality of reporting of surgical outcomes and complication data in randomised controlled trials (RCTs) on the multimodal treatment regimens for rectal cancer. The results of RCTs may have a substantial impact on future treatment regimens. In multimodal treatment regimens, the quality of surgical treatments remains of the utmost prognostic relevance to patients with rectal cancer. Over the last 40 years, rectal cancer treatment has made impressive progress by developing and implementing differentiated quality criteria for surgical treatment, including multimodal therapy concepts [1]. Heald et al. [2] proposed the notion of total mesorectal excision (TME). This has become the surgical standard for cancer of the middle and lower third of the rectum [2,3,4]. A microscopically negative circumferential resection margin (CRM), sufficient lymph node retrieval, and the integrity of the mesorectal fascia are all associated with lower local and distant recurrence rates and better long-term survival [5,6].

In studies, RCTs lower the risk of selection bias. However, this study design is not necessarily associated with high practical relevance. Reporting surgical outcomes and complication data in RCTs is essential because it is the basis for judging whether the results of a study warrant a change in clinical practice. On the contrary, low-quality data reporting or omitting critical data relevant to the overall outcome may result in misleading reports from studies. Different scales and checklists for assessing the quality of reporting in RCTs have been developed [7,8,9], including the Jadad scale, which was developed using standard techniques of scale development [10].

Another critical aspect of evaluating the efficacy of rectal cancer treatment is the need for standardised reporting of surgical outcome and complication data due to the mostly elderly population of rectal cancer patients and their associated medical comorbidities. In addition, the success of multimodal therapies for treating rectal cancer often relies on the timing of chemotherapy or radiotherapy with surgical intervention, and whether these therapies are considered successful can be affected by surgical outcome criteria and perioperative complications. Perioperative complication grading systems [11,12] and, most recently, standard criteria for reporting surgical outcomes have been established [13,14,15].

We hypothesise that the quality of reporting of surgical outcome and complication data in RCTs is high. To address this hypothesis, we conducted this systematic review. This study aimed to measure the quality of the reporting of surgical outcome and complication data in RCTs of rectal cancer treatment and to analyse the change in the reporting quality over 40 years.

## 2. Materials and Methods

This systematic review complied with the PRISMA guidelines [16]. This review was prospectively registered in an international prospective registry for systematic reviews under the study number review registry 1682.

### 2.1. Literature Search

**Figure 1:**
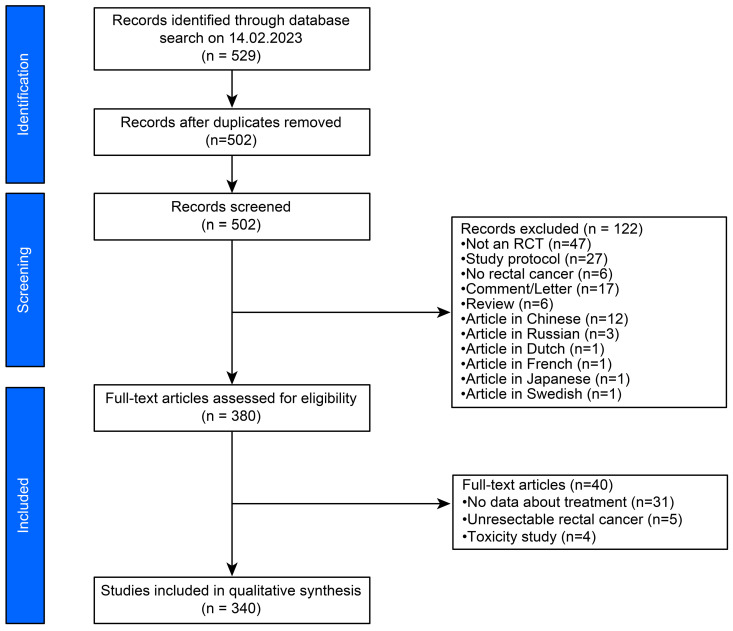
PRISMA flowchart of the literature research.

To identify studies, we performed a systematic literature search of EMBASE, MEDLINE (via PubMed), and Cochrane Central Register of Controlled Trials using the following strings: ((“rectal cancer” OR “rectal carcinoma”) AND (radiation OR radiotherapy)) (Figure 1). The results were limited to from January 1982 to 10 February 2022. Two of the authors (JJ and KU) independently checked whether the full-text articles were eligible. Disagreements were clarified through a one-by-one discussion.

The time interval from 1982 to 2022 was chosen because RCTs were infrequently published in the journals studied before this period and because of the ground-breaking insights in rectal cancer treatment Heald promoted at that time [2,17]. A subgroup analysis was performed to compare publications published before and after the year 2000, as the implementation of the TME concept and related procedure-specific criteria occurred primarily on a global scale in the new millennium. The year 2001 was chosen as the cut-off point because this was the time when these procedures were being performed—specific criteria were already in place or were recommended in many guidelines.

### 2.2. Inclusion and Exclusion Criteria

Articles that met the following criteria were selected for inclusion:

(1) population—patients with rectal cancer;
(2) treatment—radiotherapy as part of the regimen;
(3) outcomes—treatment methods;
(4) study design—RCTs;
(5) language of search results—limited to the English, German, Polish, or Italian languages.

The excluded articles were non-RCTs, literature reviews, observational epidemiological studies (cohort and case-control designs), case series, case reports, articles describing other forms of rectal cancer treatment without radiotherapy in the treatment regimen, and studies including cancers other than rectal cancer and articles are written in a language different than the above mentioned. All duplicate articles were removed, and database limits were utilised to exclude paediatric and anatomical/cadaver articles.

### 2.3. Outcome Parameters

The primary outcome was the quality of reporting in RCTs and the completeness rate of reporting of surgical outcome and complication data; this outcome was assessed based on fourteen criteria (Table 1).

The following secondary outcomes were also collected and assessed: study period, study design, the population size of rectal cancer patients, clinical data, type of treatment, country of origin, publication year, the absolute number of citations, single or multicentre study design, sample size, study intervention and control groups, study endpoints, the conclusion of the study, Quirke grade as a marker of the quality of surgical resection, the field of medical speciality, Scientific SCImago Journal Ranking, journal impact factor (IF) according to the Web of Science Group, and the type of surgical procedures performed.

An additional outcome measure was to objectively evaluate changes in procedure-specific quality criteria over time, particularly with respect to their initial consultation in previous literature.

### 2.4. Quality of Reporting

We modified previously published criteria related to the completeness of surgical outcomes and complication reporting to rate the articles [13]. Fourteen critical components were evaluated (Table 1). Providing information on surgical interventions using the 14 components allows a complete understanding of the quality of outcomes and complication reporting. As initially, the 14 criteria did not address oncological radicality in rectal cancer surgery, this was assessed separately. In particular, the category “Procedure-specific quality” was adjusted, as this outcome is highly relevant in rectal cancer surgery because they are all associated with lower local and distant recurrence rates and better long-term survival. If the items described were mentioned in the assessed articles, 1 point was awarded for each item. If an item was not noted, no point was given.

In turn, the category “quality of reporting in RCTs” was created, as these outcomes are often of interest in RCTs. The Jadad scale [10], shown previously as reliable and valid, was used. The Jadad scale is a 3-item scale covering the randomisation method, the blinding method, and withdrawals/dropouts (Supplementary Table S1). For each item, 1 point was assigned to a study if it was described as randomised or double-blinded or had discontinuations/failures. If the described randomisation or blinding method was judged appropriate, 1 point was assigned for that item.

In contrast, no point was awarded for this item if the described randomisation or blinding method was deemed inadequate. In the original Jadad scale, only double blinding was considered. The scale was modified to include single blinding because double blinding is not always possible in surgical procedures. The blinding method was considered appropriate if the item indicated who was involved in blinding and, depending on the type of intervention, possible additional measures to ensure blinding. The final quality score for each article ranged from 0 (lowest quality) to 5 points (highest quality).

We searched (in August 2023) the official relevant websites for information regarding the main characteristics of the journal IF, according to the SCI Journal website, [18] and the SJR indicator, provided by the SCImago journal and country rank. [19] Journals were classified by the IF, with an IF of 10 or more considered excellent, a value between 3 and 10 considered very good, a value between 1 and 3 considered good, and a value below 1 considered moderately good.

### 2.5. Statistical Analysis

Summary statistics were tabulated via established norms. Baseline characteristics were summarized using counts and percentages. Weighted overall rates were calculated for dichotomous data. Means were determined using normally distributed continuous data. Microsoft Excel was used to analyse the data. A *p*-value less than 0.05 (2-sided) was considered to be statistically significant.

Median and range scores for the quality were calculated for all articles and each journal. Student’s *t*-test was used to compare sample distributions. Differences were statistically significant if the *p*-value was less than 0.05.

### 2.6. Ethical Statement

The Ethical Committee of the Cantonal Hospital St. Gallen reviewed this study. Because all data used are publicly available, the study was exempt from further oversight and requirements concerning informed consent. The Strengthening the Reporting of Observational Studies in Epidemiology (STROBE) reporting guidelines were applied [20].

## 3. Results

We initially identified 529 articles (Figure 1). After excluding 27 duplicates, titles and abstracts were assessed based on the inclusion criteria. A full-text review was performed on all selected articles, after which 340 were further analysed.

### 3.1. Description of Included Studies

The 340 articles identified included outcomes for multimodal therapy in 143,531 rectal cancer patients (Supplementary Tables S2 and S3). A total of 77.1% (N = 262) of the articles had 100 or more participants. Overall, 28.9% (N = 98) of these articles recruited 500 or more participants, and the median number of participants per study was 141.5 (IQR, 106.2–582.2). Most papers comprised multisite RCTs (N = 242, 71.2%); 98 (28.8%) RCTs were performed at single sites. Most RCTs were initiated at a European research site (N = 243, 71.3%), and 335 (98.5%) were open-label. Seventy-three articles (21.5%) focused on overall survival, 73 (21.4%) focused on locoregional recurrence, 51 (15%) focused on disease-free survival, 51 (15%) concentrated on pathological complete response, and 19 (5.6%) focused on postoperative morbidity and mortality. Of the 340 articles reviewed, 69 (20.3%) were published in surgical journals, 23 (6.8%) in medical journals, 135 (39.7%) in oncology journals, and 69 (20.3%) in radiology journals. Most studies (N = 295, 86.7%) were published in journals with an IF of 3 or higher. Nearly 30% (N = 101) were published in high-impact journals with an IF of 10 or higher, such as The New England Journal of Medicine (N = 7, 2.1%) [21,22,23,24,25,26,27], The Lancet (N = 3, 0.9%) [28,29,30], Journal of Clinical Oncology (N = 36, 10.6%) [31,32,33,34,35,36,37,38,39,40,41,42,43,44,45,46,47,48,49,50,51,52,53,54,55,56,57,58,59,60,61,62,63,64,65,66], The Lancet Oncology (N = 11, 3.2%) [67,68,69,70,71,72,73,74,75,76,77], Annals of Oncology (N = 17, 5%) [78,79,80,81,82,83,84,85,86,87,88,89,90,91,92,93,94], Journal of the American Medical Association Oncology (N = 3, 0.9%) [95,96,97], Journal of the American Medical Association Surgery (N = 2, 0.6%) [98,99], Journal of the National Cancer Institute (N = 5, 1.5%) [100,101,102,103,104], Annals of Surgery (N = 12, 3.5%) [105,106,107,108,109,110,111,112,113,114,115,116], Clinical Cancer Research (N = 4, 1.2%) [117,118,119,120], and Cancer Communications (N = 1, 0.3%) [121].

### 3.2. Quality of Reporting in RCTs

All 340 articles described a randomisation method (Table 2). Five articles reported double or single blinding, and 335 reported a correctly performed randomisation method and assessment. The description of withdrawals and dropouts were reported in all 340 articles. The median scale score for the 340 articles was 4 points (range, 4–5).

### 3.3. Critical Appraisal

**Figure 2:**
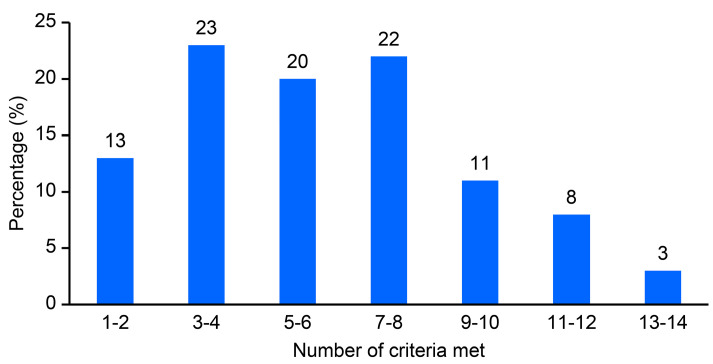
Completeness of surgical outcomes and complication data reporting in a convenience sample of the rectal treatment literature.

Of the 14 criteria related to the completeness of reporting of surgical outcomes and complication data, only seven articles (2%) [61,70,114,122,123,124,125] met all the criteria (Figure 2). Four articles (<1.0%) [71,126,127,128] met 13 criteria, and 27 articles (8%) met either 11 or 12 criteria. Additionally, 36 articles (11%) met from 9 to 10 criteria, 76 (22%) met from 7 to 8 criteria, 68 (20%) met from 5 to 6 criteria, 79 (23%) met from 3 to 4 criteria, and 43 (13%) met from 1 to 2 criteria. A mean of 6.1 ± 3.1 (SD) criteria and a median of 5.5 (IQR 5) were met. There was a linear trend in the quality of the articles, as reflected by the mean criteria met, over time (Supplementary Figure S1).

### 3.4. Quality of Reporting of Surgical Outcome and Complication Data

Table 3 shows specific reporting criteria and compliance rates for each primary endpoint. The method of data accrual (N = 340, 100%) and the duration of follow-up (N = 340, 100%) were reported consistently. Commonly underreported criteria included the complication severity (N = 51, 15%), macroscopic integrity of mesorectal excision (N = 57, 17%), length of stay (N = 61, 18%), number of retrieved lymph nodes (N = 70, 21%), distance between the tumour and CRM (N = 90, 26%), surgical radicality according to the site of the primary tumour (N = 100, 29%), and CRM status (N = 128, 38%). In the articles reporting complication severity, 4 articles (1.2%) used the simple classification of major versus minor complications, and 47 articles (13.8%) used validated grading systems [11,12]. Of the four articles using the major versus minor categorisation, four definitions were used to describe what constituted a major complication. Procedure-specific quality was reported in 172 articles (51%) (mean criteria = 1.3 ± 1.6 (SD); median criteria = 0 (range 0–5)); however, only 20 articles (5.9%) [61,69,70,71,73,74,89,98,114,115,122,123,124,125,126,129,130,131,132,133] met all five procedure-specific quality-reporting criteria, of which 6 of 20 articles were published in a surgical journal and 8 of 20 articles in an oncology journal. Most articles (N = 222, 65%) met one or no procedure-specific quality-reporting criteria.

One hundred fifty-nine articles (47%) reported one or more complications or at least indicated the duration of follow-up for the assessment of complications. One hundred eighty-one (51%) of the articles reported morbidity and mortality rates including the complications leading to death. The death rates occurred most frequently in reports of postoperative morbidity and mortality (19 articles) and least frequently in descriptions of the predictive value of different proteins (0 articles) or radiological features (0 articles). Morbidity risk factor stratification based on factors such as the American Society of Anesthesia classification, body mass index, age, and comorbid diseases for characterising the patient population was described in 128 articles (38%).

Specific reporting criteria and reporting compliance according to the study’s primary endpoint are tabulated in Table 3. Of the articles with the primary endpoint of OS (N = 73), 42 articles (58%) met less than half (less than 7) of the 14 reporting criteria (median 7 criteria, IQR 4). Procedure-specific quality was reported in only 32 of these 73 articles (44%), with the macroscopic integrity of the mesorectal excision plane being the worst rated (N = 8, 11%). The majority of articles reporting disease-free survival (DFS) (30 of 51 articles, 59%) met less than seven reporting criteria (mean = 5.9 ± 2.8 (SD); median = 6 (range 2–14)), including a failure to report the macroscopic integrity of mesorectal excision (44 of 51 articles, 86%), the distance between the tumour and CRM (42 of 51 articles, 82%), CRM status (39 of 51 articles, 76%), number of retrieved lymph nodes (40 of 51 articles, 78%), and surgical radicality according to the site of the primary tumour (30 of 51 articles, 59%). Only one study reporting the locoregional recurrence rate (LRR) met all 14 quality-reporting [122] criteria. The majority of LRR articles (49 of 73 articles, 67%) met seven or fewer reporting criteria (mean = 6.3 ± 3 (SD); median = 5.5 (range 2–14)).

Table 4 shows, for each time period and overall, the specific criteria and the fulfilment of the reporting requirements over time. Among the 73 articles published from 1984 to 2001, a median of 5 (range, 2–9) criteria were met; among the 73 articles published from 2002 to 2009, a median of 6 (range, 2–11) criteria were satisfied; and among the 194 articles published from 2010 to 2022, a median of 6 (range, 2–14) criteria were met. The reporting quality pertaining to the procedure-specific criteria has shown little to no improvement over the years. During the intervals 2000–2004, 2005–2009, 2010–2014, 2015–2019, and 2020–2022, the median criteria values were 0 (range 0–4), 1 (range 0–4), 1 (range 0–5), 1 (range 0–5), and 1 (range 0–5), respectively (Supplementary Figure S2).

The median value of the criteria met for RCTs published in oncology journals was 6 (range 2–14), and it was 7 (range 2–14) for those in surgical journals, 4.5 (range 2–11) for those in medical journals, and 5 (range 0–13) for those in radiology journals (Table 5).

## 4. Discussion

In this critical review of the multimodal rectal cancer treatment literature from over 40 years, we demonstrate considerable inconsistency in the quality of reporting of surgical outcome and complication data. The quality of surgical outcomes and reporting of complication data in articles on RCTs on rectal cancer are essential in the assessment of outcomes and areas for improving the quality of surgical care. An accurate appraisal of surgical quality requires consistency and reliability in the reporting of outcomes, data on specific complications, and procedure-specific quality.

In the study presented here, only 44% of the RCTs related to multimodal rectal cancer treatment reported on a minimum of 7 out of 14 critical surgical reporting criteria. Further, only half of the articles (N = 168, 49%) met the procedure-specific quality criteria reported in the article. Interestingly, surgical articles did not have a higher rate of better compliance regarding reporting procedure-specific quality criteria compared to oncology journals. Only 53% of the articles adequately defined the complications reported in the study. There was substantial variability in the published articles regarding what constituted a complication. The reporting of the complication severity using grading criteria was very limited (15%), and appropriate consideration of risk factors for surgical complications was described in only 38% of the articles. This lack of surgical quality reporting poses a significant challenge to synthesising and analysing the data for surgical interventions and multimodal treatment and potentially confounds the comparison of outcomes across studies.

Consistency in surgical quality criteria reporting would facilitate data comparison across studies and enable authors to perform better meta-analyses.

Although significant progress has been made in improving the reporting of surgical outcomes, our study indicates that further efforts are needed. Our results support the work of others [13,134] who have demonstrated the limitations in how surgical quality outcomes and complications are reported in the surgical literature.

The current study emphasises the need for the use of standard grading systems [11,12] to characterise the severity of complications after rectal cancer surgery. In the surgical literature, there are two areas that have received substantial attention regarding complication reporting: measuring the severity of complications and developing standardised outcome measures and clear definitions of complications. For example, the term “surgical site infection” provides a broad spectrum of different diagnoses. Data on “surgical site infection” are, therefore, only comparable between different studies or further computed in meta-analyses with caution.

The consistent reporting of standardised procedure-specific quality criteria is of equal importance to reporting the severity of complications. Curative surgical therapy of rectal cancer usually requires partial or complete excision of the mesorectum and, thus, the regional lymphatic drainage area and resection of the primary tumour in healthy tissue. Surgical therapy should include the following principles: complete mesorectal excision, CRM status, R0 status depending on the location of the primary tumour, a distance between the tumour and CRM of >1 mm, sufficient lymph nodes removed, and complete resection of the mesorectum. All these procedure-specific quality criteria influence OS, DFS, and LLR. However, our study shows that current strategies for reporting these data complicate the comparison of different multimodal therapy regimens and, thus, could be better. For example, if an attempt is made to compare two neoadjuvant therapy protocols wherein the surgical quality is described in one study but not in the other, it is difficult to determine which effect is from the neoadjuvant therapy and which is from the surgery.

When evaluating the procedure-specific quality criteria, it is important to note that the treatment of rectal cancer has changed dramatically over the past 40 years; nevertheless, it must be assumed that years or even decades are required before a novel surgical technique becomes an accepted standard. TME was introduced in 1982 [2], and the distance between the tumour and circumferential resection margin was introduced in 1986 [135]. It took several years to gain acceptance, so TME has only been considered the procedure of choice since 2004 [136]. It can be assumed that studies published before 2004 describe many fewer procedure-specific quality criteria than studies published after 2004 because the criteria did not yet exist. Interestingly, we found that in studies published after 2004 and even after 2020, the rate of reporting procedure-specific quality criteria remained low, at 15–45%, depending on the criterion.

The limitation of the study was that only randomized control trials were used. Selection bias may limit the generalizability of our findings. More recent articles may reflect improvements in complication reporting not captured in this study. A further limitation is the evaluation criteria used. The criteria used here were previously published and utilised in several studies to evaluate complication data. However, we acknowledge that these criteria are not universally accepted. Unfortunately, while checklists such as the STROBE statement [20] exist for evaluating what items should be reported in different studies, there are no universally accepted guidelines for assessing the quality of reporting on specific complications. Thus, we chose to use these criteria and modify them because they represent a practical and easy-to-understand strategy for characterising the quality of complication reporting. We further acknowledge that some criteria are not ubiquitous in their relevance. For example, it is not always possible to account for complication risk factors if the relationship to the outcome is not established. Another possible limitation is the time period that was chosen. The introduction of three procedure-specific quality criteria after 1982 may have had an effect on the results. To counteract this, we performed a subgroup analysis. The time periods were as follows: 1984–2001 and 2002–2022. The year 2001 was chosen as the reference period because this was the time when these procedures were performed—specific criteria already existed or were recommended in many guidelines. We also wanted to analyse how a surgical quality criterion became established in RCTs over time, so we chose the time when a method was first described.

This study demonstrates inconsistent quality in the reporting of surgical outcome and complication data in RCTs of multimodal rectal cancer treatment. The variability in reporting these data complicates the comparison of surgical outcomes between trials and potentially dilutes its importance for the further clinical development of the treatment.

## 5. Conclusions

The future development of guidelines for the accurate, comparable, and reproducible collection and reporting of quality surgical data is essential for future RCTs on multimodal rectal cancer treatment.

## Figures and Tables

**Table 1:** Criteria utilised to evaluate surgical quality reporting in randomised clinical trials of the treatment of rectal cancer (modified from Martin RCG) [13].

Requirement	Points
Study design/method of accruing data	
Article indicates whether the data were collected prospectively or retrospectively.	+1
Article does not specify whether the data were collected prospectively or retrospectively.	0
Duration of follow-up	
Article indicates how long the patients were followed and evaluated for complications.	+1
Article does not specify how long the patients were observed and evaluated for complications.	0
Definition of complications	
Article defines at least one complication with specific inclusion criteria.	+1
Article does not define the complications.	0
Mortality rate and causes of death	
Number of patients who died in the postoperative period of study are recorded together with cause of death.	+1
Mortality data are not provided.	0
Morbidity rate and total complications	
Number of patients with any complication and the total number of complications are recorded.	+1
Morbidity data are not provided.	0
Severity grade utilized	
Any grading system designed to clarify the severity of complications including “major and minor” is reported.	+1
No arbitrary grading system is given to clarify the severity of complications including “serious and minor.”	0
Procedure-specific quality	
Circumferential resection margin- (CRM-) status	
Circumferential resection margin- (CRM-) status described.	+1
Circumferential resection margin- (CRM-) status not described.	0
Surgical radicality according to the site of the primary tumour ^a^	
Surgical radicality according to the site of the primary tumour described.	+1
Surgical radicality according to the site of the primary tumour not described.	0
Distance between the tumour and circumferential resection margin	
CRM distance described.	+1
CRM distance not described.	0
Number of retrieved lymph nodes	
Number of retrieved lymph nodes described.	+1
Number of retrieved lymph nodes not described.	0
Macroscopic intactness of mesorectal excision ^b^	
Macroscopic intactness of mesorectal excision described.	+1
Macroscopic intactness of mesorectal excision not described.	0
Type of surgery	
Type of surgery described.	+1
Type of surgery not described.	0
Length of stay	
Median or mean length of stay indicated in the study.	+1
No data on mean or average length of stay.	0
Risk factors included in the analysis	
Evidence of risk stratification and method used indicated in the study.	+1
No information on risk stratification or the method used is given in the study.	0

Notes: ^a^ R0 vs. R1 + R2 vs. unresected patients. ^b^ The non-peritonealized surface of the fresh specimen is examined circumferentially, and the completeness of the mesorectum is scored as follows: complete vs. near complete vs. incomplete vs. cannot be determined. Adapted from Martin et al. [13].

**Table 2:** Number of articles for each Jadad scale item [10].

Jadad Scale Items	Oncology Journal, n (%) n = 135	Surgical Journal, n (%) n = 69	Medical Journal, n (%) n = 23	Radiology Journal, n (%) n = 69	Gastroenterology Journal, n (%) n = 41	Total, n (%) n = 340
Randomisation						
Study described as randomised	135 (100)	69 (100)	23 (100)	69 (100)	41 (100)	340 (100)
Randomisation method described and appropriate	135 (100)	69 (100)	23 (100)	69 (100)	41 (100)	340 (100)
Randomisation method described and inappropriate	0 (0)	0 (0)	0 (0)	0 (0)	0 (0)	0 (0)
Randomisation method not described	0 (0)	0 (0)	0 (0)	0 (0)	0 (0)	0 (0)
Blinding						
Study described as double-blind (or single-blind)	0 (0)	1 (1.4)	1 (4.3)	2 (2.9)	1 (2.4)	5 (1.5)
Blinding method described and appropriate	135 (100)	68 (98.6)	22 (95.7)	67 (97.1)	40 (97.6)	335 (98.5)
Blinding method described and inappropriate	0 (0)	0 (0)	0 (0)	0 (0)	0 (0)	0 (0)
Blinding method not described	0 (0)	0 (0)	0 (0)	0 (0)	0 (0)	0 (0)
Study not described as blind	0 (0)	0 (0)	0 (0)	0 (0)	0 (0)	0 (0)
Withdrawals and dropouts						
Withdrawals and dropouts described	135 (100)	69 (100)	23 (100)	67 (100)	41 (100)	340 (100)
Withdrawals and dropouts not described	0 (0)	0 (0)	0 (0)	0 (0)	0 (0)	0 (0)

Notes: Adapted from Jadad et al. [10].

**Table 3:** Specific criteria and reporting compliance according to the primary endpoint.

Reporting Criterion	Primary Endpoint												Total, n (%)
	Overall Survival, n (%) n = 73	Disease-Free Survival, n (%) n = 51	Locoregional Recurrence, n (%) n = 73	Pathological Complete Response, n (%) n = 51	Pathological Features, n (%) n = 12	R0-Resection Rate, n (%) n = 8	Treatment-Related Toxicity, n (%) n = 24	Postoperative Morbidity and Mortality, n (%) n = 19	Functional Outcome, n (%) n = 19	Quality of Life, n (%) n = 18	Predictive Value of Different Proteins, n (%) n = 17	Radiological Features, n (%) N = 4	N = 340
Method of accruing data defined	73 (100)	51 (100)	73 (100)	51 (100)	12 (100)	8 (100)	24 (100)	19 (100)	19 (100)	18 (100)	17 (100)	4 (100)	340 (100)
Duration of follow-up indicated	73 (100)	51 (100)	73 (100)	51 (100)	12 (100)	8 (100)	24 (100)	19 (100)	19 (100)	18 (100)	17 (100)	4 (100)	340 (100)
Definitions of complications provided	35 (48)	24 (47)	33 (45)	29 (57)	2 (17)	7 (88)	10 (42)	19 (100)	11 (58)	5 (28)	0 (0)	0 (0)	159 (47)
Mortality rate, cause of death listed	39 (53)	25 (49)	39 (53)	32 (63)	2 (17)	7 (88)	11 (46)	19 (100)	10 (53)	6 (33)	0 (0)	0 (0)	174 (51)
Morbidity rate and total complications reported	37 (51)	24 (47)	38 (52)	31 (61)	2 (17)	7 (88)	10 (42)	19 (100)	12 (63)	6 (33)	1 (6)	0 (0)	173 (51)
Severity grade utilized	8 (11)	9 (18)	5 (7)	13 (25)	2 (17)	3 (38)	4 (17)	4 (21)	3 (16)	3 (17)	0 (0)	0 (0)	51 (15)
Procedure-specific quality													
CRM-status	22 (30)	12 (24)	35 (48)	31 (61)	9 (75)	7 (88)	4 (17)	4 (21)	3 (16)	4 (22)	5 (29)	1 (25)	128 (38)
Surgical radicality according to the site of the primary tumour ^a^	19 (26)	21 (41)	17 (23)	26 (51)	1 (8)	7 (88)	2 (8)	6 (32)	3 (16)	2 (11)	5 (29)	0 (0)	100 (29)
Distance between the tumour and CRM	16 (22)	9 (18)	21 (29)	22 (43)	6 (50)	5 (63)	1 (4)	5 (26)	2 (11)	4 (22)	5 (29)	1 (25)	90 (26)
Number of retrieved lymph nodes	15 (21)	11 (22)	18 (25)	17 (33)	7 (58)	2 (25)	0 (0)	4 (21)	2 (11)	1 (6)	0 (0)	0 (0)	70 (21)
Macroscopic intactness of mesorectal excision ^b^	8 (11)	7 (14)	12 (16)	10 (20)	8 (67)	2 (25)	2 (8)	3 (16)	0 (0)	1 (6)	6 (35)	1 (25)	57 (17)
Type of surgery indicated	44 (60)	33 (65)	56 (77)	36 (71)	10 (83)	5 (63)	13 (54)	17 (89)	11 (58)	12 (67)	4 (24)	1 (25)	222 (65)
Length of stay data reported	11 (15)	8 (16)	11 (15)	9 (18)	2 (17)	3 (38)	3 (13)	13 (68)	2 (11)	1 (6)	0 (0)	0 (0)	61 (18)
Risk factors included in analysis	17 (23)	17 (33)	29 (40)	24 (47)	3 (25)	8 (100)	7 (29)	15 (79)	9 (47)	6 (33)	2 (12)	1 (25)	130 (38)

Abbreviations: DFS: disease-free survival; LRR: locoregional recurrence rate; MoMo: postoperative morbidity and mortality; OS: overall survival; pCR: pathological complete response; CRM: Circumferential resection margin. Notes: ^a^ R0 vs. R1 + R2 vs. unresected patients. ^b^ The non-peritonealized surface of the fresh specimen is examined circumferentially, and the completeness of the mesorectum is scored as follows: complete vs. near complete vs. incomplete vs. cannot be determined. Adapted from Martin et al. [13].

**Table 4:** Specific criteria and reporting compliance according to time.

Reporting Criteria	Time Period Study Published								Total
	1984–1989	1990–1994	1995–1999	2000–2004	2005–2009	2010–2014	2015–2019	2020–2022	
	N = 11	N = 19	N = 28	N = 33	N = 55	N = 71	N = 83	N = 40	N = 340
Method of accruing data defined	11 (100%)	19 (100%)	28 (100%)	33 (100%)	55 (100%)	71 (100%)	83 (100%)	40 (100%)	340 (100%)
Duration of follow-up indicated	11 (100%)	19 (100%)	28 (100%)	33 (100%)	55 (100%)	71 (100%)	83 (100%)	40 (100%)	340 (100%)
Definitions of complications provided	7 (64%)	10 (53%)	13 (46%)	11 (33%)	26 (47%)	33 (47%)	35 (42%)	24 (60%)	159 ((47%)
Mortality rate, cause of death listed	8 (73%)	15 (79%)	16 (57%)	13 (39%)	26 (47%)	33 (47%)	37 (45%)	25 (63%)	174 (51%)
Morbidity rate and total complications	8 (73%)	12 (63%)	15 (54%)	12 (36%)	29 (53%)	33 (47%)	39 (47%)	25 (63%)	173 (51%)
Severity grade utilized	0 (0%)	1 (5%)	1 (4%)	3 (9%)	6 (11%)	6 (8%)	18 (22%)	16 (40%)	51 (15%)
Procedure-specific quality									
CRM status	0 (0%)	3 (16%)	7 (25%)	14 (42%)	20 (36%)	33 (47%)	33 (40%)	18 (45%)	128 (38%)
Surgical radicality according to site of primary tumour ^a^	0 (0%)	0 (0%)	1 (4%)	4 (12%)	11 (20%)	34 (48%)	36 (43%)	14 (35%)	100 (29%)
Distance between tumour and CRM	0 (0%)	0 (0%)	1 (4%)	5 (15%)	12 (22%)	28 (39%)	29 (35%)	15 (38%)	90 (26%)
Numbers of retrieved lymph nodes	0 (0%)	0 (0%)	3 (11%)	5 (15%)	17 (31%)	21 (30%)	18 (22%)	6 (15%)	70 (21%)
Macroscopic intactness of mesorectal excision ^b^	0 (0%)	0 (0%)	1 (4%)	5 (15%)	3 (5%)	12 (17%)	24 (29%)	12 (30%)	57 (17%)
Type of Surgery	9 (82%)	16 (84%)	17 (61%)	24 (73%)	34 (62%)	49 (69%)	47 (57%)	26 (65%)	222 (65%)
Length of stay data reported	7 (64%)	4 (21%)	1 (4%)	3 (9%)	7 (13%)	11 (17%)	19 (23%)	9 (23%)	61 (18%)
Risk factors included in analysis	1 (9%)	4 (21%)	5 (18%)	5 (15%)	18 (33%)	23 (32%)	42 (51%)	32 (80%)	130 (38%)

Abbreviations: CRM: Circumferential resection margin. Notes: ^a^ R0 vs. R1 + R2 vs. unresected patients; ^b^ The non-peritonealized surface of the fresh specimen is examined circumferentially, and the completeness of the mesorectum is scored as follows: complete vs. near complete vs. incomplete vs. cannot be determined. Adapted from Martin et al. [13].

**Table 5:** Specific criteria and reporting compliance according to the type of journal.

Reporting Criterion	Type of Journal						Total, n (%)
	Oncology, n (%) n = 135	Surgical, n (%) n = 69	Medical, n (%) n = 23	Radiology, n (%) n = 69	Gastroenterology, n (%) n = 41	Other, n (%) n = 3	n = 340
Method of accruing data defined	135 (100)	69 (100)	23 (100)	69 (100)	41 (100)	3 (100)	340 (100)
Duration of follow-up indicated	135 (100)	69 (100)	23 (100)	69 (100)	41 (100)	3 (100)	340 (100)
Definitions of complications provided	57 (42)	45 (65)	7 (30)	22 (32)	27 (66)	1 (33)	159 (47)
Mortality rate, cause of death listed	63 (47)	44 (64)	11 (48)	25 (36)	30 (73)	1 (33)	174 (51)
Morbidity rate and total complications reported	61 (45)	45 (65)	10 (43)	28 (41)	28 (68)	1 (33)	173 (51)
Severity grade utilized	17 (13)	11 (16)	1 (4)	10 (14)	12 (29)	0 (0)	51 (15)
Procedure-specific quality							
CRM status	54 (40)	26 (38)	5 (22)	25 (36)	17 (41)	1 (33)	128 (38)
Surgical radicality according to the site of the primary tumour ^a^	51 (38)	14 (20)	3 (13)	20 (29)	12 (29)	0 (0)	100 (29)
Distance between the tumour and CRM	38 (28)	21 (30)	3 (13)	16 (23)	12 (29)	0 (0)	90 (26)
Number of retrieved lymph nodes	25 (19)	13 (19)	5 (22)	15 (22)	11 (27)	1 (33)	70 (21)
Macroscopic intactness of mesorectal excision ^b^	25 (19)	13 (19)	2 (9)	7 (10)	9 (22)	1 (33)	57 (17)
Type of surgery	84 (62)	54 (78)	13 (57)	40 (58)	31 (76)	0 (0)	222 (65)
Length of stay data reported	23 (17)	22 (32)	3 (13)	3 (4)	10 (24)	0 (0)	61 (18)
Risk factors included in analysis	48 (36)	32 (46)	7 (30)	17 (25)	26 (63)	0 (0)	130 (38)

Abbreviations: DFS: disease-free survival; LRR: locoregional recurrence rate; MoMo: postoperative morbidity and mortality; OS: overall survival; pCR: pathological complete response; CRM: Circumferential resection margin. Notes: ^a^ R0 vs. R1 + R2 vs. unresected patients. ^b^ The non-peritonealized surface of the fresh specimen is examined circumferentially, and the completeness of the mesorectum is scored as follows: complete vs. near complete vs. incomplete vs. cannot be determined. Adapted from Martin et al. [13].

## Data Availability

The authors confirm that the data supporting the findings of this study are available within the article and its Supplementary Materials.

## Supplementary Materials

The following supporting information can be downloaded at: https://www.mdpi.com/article/10.3390/cancers16010026/s1, Figure S1. Analysis of mean quality criteria met from 1984 to 2022. The figure demonstrates no significant linear trend between the quality of articles; Figure S2. Analysis of Procedure-specific quality criteria met from 1984 to 2022. The figure demonstrates no significant linear trend between the quality of articles; Table S1. Jadad scale for assessing the quality of reporting in randomised clinical trials of rectal cancer treatments (modified from Jadad et al.); Table S2. Characteristics and publication output of RCTs of multimodal rectal cancer treatment; Table S3. Baseline characteristics of the included studies.

## References

[1] De Caluwé L., Van Nieuwenhove Y., Ceelen W.P. (2013). Preoperative chemoradiation versus radiation alone for stage II and III resectable rectal cancer. Cochrane Database Syst. Rev..

[2] Heald R.J., Husband E.M., Ryall R.D. (1982). The mesorectum in rectal cancer surgery—the clue to pelvic recurrence?. Br. J. Surg..

[3] Fielding L.P., Arsenault P.A., Chapuis P.H., Dent O., Gathright B., Hardcastle J.D., Hermanek P., Jass J.R., Newland R.C. (1991). Clinicopathological staging for colorectal cancer: An International Documentation System (IDS) and an International Comprehensive Anatomical Terminology (ICAT). J. Gastroenterol. Hepatol..

[4] Bokey E.L., Ojerskog B., Chapuis P.H., Dent O.F., Newland R.C., Sinclair G. (1999). Local recurrence after curative excision of the rectum for cancer without adjuvant therapy: Role of total anatomical dissection. Br. J. Surg..

[5] Quirke P., Steele R., Monson J., Grieve R., Khanna S., Couture J., O’Callaghan C., Myint A.S., Bessell E., Thompson L.C. (2009). Effect of the plane of surgery achieved on local recurrence in patients with operable rectal cancer: A prospective study using data from the MRC CR07 and NCIC-CTG CO16 randomised clinical trial. Lancet.

[6] Kusters M., Marijnen C.A., van de Velde C.J., Rutten H.J., Lahaye M.J., Kim J.H., Beets-Tan R.G., Beets G.L. (2010). Patterns of local recurrence in rectal cancer; a study of the Dutch TME trial. Eur. J. Surg. Oncol..

[7] Scherer R.W., Crawley B. (1998). Reporting of randomized clinical trial descriptors and use of structured abstracts. JAMA.

[8] Moher D., Jones A., Lepage L., Consort Group (2001). Use of the CONSORT statement and quality of reports of randomized trials: A comparative before-and-after evaluation. JAMA.

[9] Moher D., Jadad A.R., Nichol G., Penman M., Tugwell P., Walsh S. (1995). Assessing the quality of randomized controlled trials: An annotated bibliography of scales and checklists. Control Clin. Trials.

[10] Jadad A.R., Moore R.A., Carroll D., Jenkinson C., Reynolds D.J., Gavaghan D.J., McQuay H.J. (1996). Assessing the quality of reports of randomized clinical trials: Is blinding necessary?. Control Clin. Trials.

[11] Dindo D., Demartines N., Clavien P.A. (2004). Classification of surgical complications: A new proposal with evaluation in a cohort of 6336 patients and results of a survey. Ann. Surg..

[12] Slankamenac K., Graf R., Barkun J., Puhan M.A., Clavien P.A. (2013). The comprehensive complication index: A novel continuous scale to measure surgical morbidity. Ann. Surg..

[13] Martin R.C., Brennan M.F., Jaques D.P. (2002). Quality of complication reporting in the surgical literature. Ann. Surg..

[14] Khuri S.F. (2005). The NSQIP: A new frontier in surgery. Surgery.

[15] Pomposelli J.J., Gupta S.K., Zacharoulis D.C., Landa R., Miller A., Nanda R. (1997). Surgical complication outcome (SCOUT) score: A new method to evaluate quality of care in vascular surgery. J. Vasc. Surg..

[16] Tricco A.C., Lillie E., Zarin W., O’Brien K.K., Colquhoun H., Levac D., Moher D., Peters M.D.J., Horsley T., Weeks L. (2018). PRISMA extension for scoping reviews (PRISMA-ScR): Checklist and explanation. Ann. Intern. Med..

[17] Heald R.J. (1979). A new approach to rectal cancer. Br. J. Hosp. Med..

[18] SCI Journal Check the Latest Impact Factor. https://www.scijournal.org.

[19] SCImago Scimago Journal & Country Rank. https://www.scimagojr.com/index.php.

[20] von Elm E., Altman D.G., Egger M., Pocock S.J., Gotzsche P.C., Vandenbroucke J.P., Strobe Initiative (2007). The strengthening the reporting of observational studies in epidemiology (STROBE) statement: Guidelines for reporting observational studies. Lancet.

[21] Gastrointestinal Tumor Study Group (1985). Prolongation of the disease-free interval in surgically treated rectal carcinoma. N. Engl. J. Med..

[22] Krook J.E., Moertel C.G., Gunderson L.L., Wieand H.S., Collins R.T., Beart R.W., Kubista T.P., Poon M.A., Meyers W.C., Mailliard J.A. (1991). Effective surgical adjuvant therapy for high-risk rectal carcinoma. N. Engl. J. Med..

[23] O’Connell M.J., Martenson J.A., Wieand H.S., Krook J.E., Macdonald J.S., Haller D.G., Mayer R.J., Gunderson L.L., Rich T.A. (1994). Improving adjuvant therapy for rectal cancer by combining protracted-infusion fluorouracil with radiation therapy after curative surgery. N. Engl. J. Med..

[24] Cedermark B., Dahlberg M., Glimelius B., Pahlman L., Rutqvist L.E., Wilking N., Swedish Rectal Cancer Trial (1997). Improved survival with preoperative radiotherapy in resectable rectal cancer. N. Engl. J. Med..

[25] Kapiteijn E., Marijnen C.A., Nagtegaal I.D., Putter H., Steup W.H., Wiggers T., Rutten H.J., Pahlman L., Glimelius B., van Krieken J.H. (2001). Preoperative radiotherapy combined with total mesorectal excision for resectable rectal cancer. N. Engl. J. Med..

[26] Sauer R., Becker H., Hohenberger W., Rodel C., Wittekind C., Fietkau R., Martus P., Tschmelitsch J., Hager E., Hess C.F. (2004). Preoperative versus postoperative chemoradiotherapy for rectal cancer. N. Engl. J. Med..

[27] Bosset J.F., Collette L., Calais G., Mineur L., Maingon P., Radosevic-Jelic L., Daban A., Bardet E., Beny A., Ollier J.C. (2006). Chemotherapy with preoperative radiotherapy in rectal cancer. N. Engl. J. Med..

[28] Medical Research Council Rectal Cancer Working Party (1996). Randomised trial of surgery alone versus surgery followed by radiotherapy for mobile cancer of the rectum. Lancet.

[29] Sebag-Montefiore D., Stephens R.J., Steele R., Monson J., Grieve R., Khanna S., Quirke P., Couture J., de Metz C., Myint A.S. (2009). Preoperative radiotherapy versus selective postoperative chemoradiotherapy in patients with rectal cancer (MRC CR07 and NCIC-CTG C016): A multicentre, randomised trial. Lancet.

[30] Rullier E., Rouanet P., Tuech J.J., Valverde A., Lelong B., Rivoire M., Faucheron J.L., Jafari M., Portier G., Meunier B. (2017). Organ preservation for rectal cancer (GRECCAR 2): A prospective, randomised, open-label, multicentre, phase 3 trial. Lancet.

[31] Gastrointestinal Tumor Study Group (1992). Radiation therapy and fluorouracil with or without semustine for the treatment of patients with surgical adjuvant adenocarcinoma of the rectum. J. Clin. Oncol..

[32] Hoover H.C., Brandhorst J.S., Peters L.C., Surdyke M.G., Takeshita Y., Madariaga J., Muenz L.R., Hanna M.G. (1993). Adjuvant active specific immunotherapy for human colorectal cancer: 6.5-year median follow-up of a phase III prospectively randomized trial. J. Clin. Oncol..

[33] Tepper J.E., O’Connell M.J., Petroni G.R., Hollis D., Cooke E., Benson A.B., Cummings B., Gunderson L.L., Macdonald J.S., Martenson J.A. (1997). Adjuvant postoperative fluorouracil-modulated chemotherapy combined with pelvic radiation therapy for rectal cancer: Initial results of intergroup 0114. J. Clin. Oncol..

[34] Francois Y., Nemoz C.J., Baulieux J., Vignal J., Grandjean J.P., Partensky C., Souquet J.C., Adeleine P., Gerard J.P. (1999). Influence of the interval between preoperative radiation therapy and surgery on downstaging and on the rate of sphincter-sparing surgery for rectal cancer: The Lyon R90-01 randomized trial. J. Clin. Oncol..

[35] Marijnen C.A., Nagtegaal I.D., Kranenbarg E.K., Hermans J., van de Velde C.J., Leer J.W., van Krieken J.H., Pathology Review Committee the Cooperative Clinical Investigators (2001). No downstaging after short-term preoperative radiotherapy in rectal cancer patients. J. Clin. Oncol..

[36] Tepper J.E., O’Connell M.J., Niedzwiecki D., Hollis D., Compton C., Benson A.B., Cummings B., Gunderson L., Macdonald J.S., Mayer R.J. (2001). Impact of number of nodes retrieved on outcome in patients with rectal cancer. J. Clin. Oncol..

[37] Lee J.H., Lee J.H., Ahn J.H., Bahng H., Kim T.W., Kang Y.K., Lee K.H., Kim J.C., Yu C.S., Kim J.H. (2002). Randomized trial of postoperative adjuvant therapy in stage II and III rectal cancer to define the optimal sequence of chemotherapy and radiotherapy: A preliminary report. J. Clin. Oncol..

[38] Nagtegaal I.D., van de Velde C.J., van der Worp E., Kapiteijn E., Quirke P., van Krieken J.H., Cooperative Clinical Investigators of the Dutch Colorectal Cancer Group (2002). Macroscopic evaluation of rectal cancer resection specimen: Clinical significance of the pathologist in quality control. J. Clin. Oncol..

[39] Gerard J.P., Chapet O., Nemoz C., Hartweig J., Romestaing P., Coquard R., Barbet N., Maingon P., Mahe M., Baulieux J. (2004). Improved sphincter preservation in low rectal cancer with high-dose preoperative radiotherapy: The lyon R96-02 randomized trial. J. Clin. Oncol..

[40] van den Brink M., Stiggelbout A.M., van den Hout W.B., Kievit J., Kranenbarg E.K., Marijnen C.A., Nagtegaal I.D., Rutten H.J., Wiggers T., van de Velde C.J. (2004). Clinical nature and prognosis of locally recurrent rectal cancer after total mesorectal excision with or without preoperative radiotherapy. J. Clin. Oncol..

[41] Bosset J.F., Calais G., Mineur L., Maingon P., Radosevic-Jelic L., Daban A., Bardet E., Beny A., Briffaux A., Collette L. (2005). Enhanced tumorocidal effect of chemotherapy with preoperative radiotherapy for rectal cancer: Preliminary results—EORTC 22921. J. Clin. Oncol..

[42] Folkesson J., Birgisson H., Pahlman L., Cedermark B., Glimelius B., Gunnarsson U. (2005). Swedish Rectal Cancer Trial: Long lasting benefits from radiotherapy on survival and local recurrence rate. J. Clin. Oncol..

[43] Nagtegaal I.D., van de Velde C.J., Marijnen C.A., van Krieken J.H., Quirke P., Dutch Colorectal Cancer Group, Pathology Review Committee (2005). Low rectal cancer: A call for a change of approach in abdominoperineal resection. J. Clin. Oncol..

[44] Rödel C., Martus P., Papadoupolos T., Füzesi L., Klimpfinger M., Fietkau R., Liersch T., Hohenberger W., Raab R., Sauer R. (2005). Prognostic significance of tumor regression after preoperative chemoradiotherapy for rectal cancer. J. Clin. Oncol..

[45] Mohiuddin M., Winter K., Mitchell E., Hanna N., Yuen A., Nichols C., Shane R., Hayostek C., Willett C., Radiation Therapy Oncology Group Trial (2006). Randomized phase II study of neoadjuvant combined-modality chemoradiation for distal rectal cancer: Radiation therapy oncology group trial 0012. J. Clin. Oncol..

[46] Smalley S.R., Benedetti J.K., Williamson S.K., Robertson J.M., Estes N.C., Maher T., Fisher B., Rich T.A., Martenson J.A., Kugler J.W. (2006). Phase III trial of fluorouracil-based chemotherapy regimens plus radiotherapy in postoperative adjuvant rectal cancer: GI INT 0144. J. Clin. Oncol..

[47] Collette L., Bosset J.F., den Dulk M., Nguyen F., Mineur L., Maingon P., Radosevic-Jelic L., Pierart M., Calais G., European Organisation for Research and Treatment of Cancer Radiation Oncology Group (2007). Patients with curative resection of cT3-4 rectal cancer after preoperative radiotherapy or radiochemotherapy: Does anybody benefit from adjuvant fluorouracil-based chemotherapy? A trial of the European organisation for research and treatment of cancer radiation oncology group. J. Clin. Oncol..

[48] Braendengen M., Tveit K.M., Berglund A., Birkemeyer E., Frykholm G., Påhlman L., Wiig J.N., Byström P., Bujko K., Glimelius B. (2008). Randomized phase III study comparing preoperative radiotherapy with chemoradiotherapy in nonresectable rectal cancer. J. Clin. Oncol..

[49] Roh M.S., Colangelo L.H., O’Connell M.J., Yothers G., Deutsch M., Allegra C.J., Kahlenberg M.S., Baez-Diaz L., Ursiny C.S., Petrelli N.J. (2009). Preoperative multimodality therapy improves disease-free survival in patients with carcinoma of the rectum: NSABP R-03. J. Clin. Oncol..

[50] Fernández-Martos C., Pericay C., Aparicio J., Salud A., Safont M., Massuti B., Vera R., Escudero P., Maurel J., Marcuello E. (2010). Phase II, randomized study of concomitant chemoradiotherapy followed by surgery and adjuvant capecitabine plus oxaliplatin (CAPOX) compared with induction CAPOX followed by concomitant chemoradiotherapy and surgery in magnetic resonance imaging-defined, locally advanced rectal cancer: Grupo cancer de recto 3 study. J. Clin. Oncol..

[51] Gérard J.P., Azria D., Gourgou-Bourgade S., Martel-Laffay I., Hennequin C., Etienne P.L., Vendrely V., François E., de La Roche G., Bouché O. (2010). Comparison of two neoadjuvant chemoradiotherapy regimens for locally advanced rectal cancer: Results of the phase III trial ACCORD 12/0405-Prodige 2. J. Clin. Oncol..

[52] Stephens R.J., Thompson L.C., Quirke P., Steele R., Grieve R., Couture J., Griffiths G.O., Sebag-Montefiore D. (2010). Impact of short-course preoperative radiotherapy for rectal cancer on patients’ quality of life: Data from the Medical Research Council CR07/National Cancer Institute of Canada Clinical Trials Group C016 randomized clinical trial. J. Clin. Oncol..

[53] Aschele C., Cionini L., Lonardi S., Pinto C., Cordio S., Rosati G., Artale S., Tagliagambe A., Ambrosini G., Rosetti P. (2011). Primary tumor response to preoperative chemoradiation with or without oxaliplatin in locally advanced rectal cancer: Pathologic results of the STAR-01 randomized phase III trial. J. Clin. Oncol..

[54] Dewdney A., Cunningham D., Tabernero J., Capdevila J., Glimelius B., Cervantes A., Tait D., Brown G., Wotherspoon A., de Castro D.G. (2012). Multicenter randomized phase II clinical trial comparing neoadjuvant oxaliplatin, capecitabine, and preoperative radiotherapy with or without cetuximab followed by total mesorectal excision in patients with high-risk rectal cancer (EXPERT-C). J. Clin. Oncol..

[55] Gérard J.P., Azria D., Gourgou-Bourgade S., Martel-Lafay I., Hennequin C., Etienne P.L., Vendrely V., François E., de La Roche G., Bouché O. (2012). Clinical outcome of the ACCORD 12/0405 PRODIGE 2 randomized trial in rectal cancer. J. Clin. Oncol..

[56] Ngan S.Y., Burmeister B., Fisher R.J., Solomon M., Goldstein D., Joseph D., Ackland S.P., Schache D., McClure B., McLachlan S.A. (2012). Randomized trial of short-course radiotherapy versus long-course chemoradiation comparing rates of local recurrence in patients with T3 rectal cancer: Trans-Tasman Radiation Oncology Group trial 01.04. J. Clin. Oncol..

[57] Sauer R., Liersch T., Merkel S., Fietkau R., Hohenberger W., Hess C., Becker H., Raab H.R., Villanueva M.T., Witzigmann H. (2012). Preoperative versus postoperative chemoradiotherapy for locally advanced rectal cancer: Results of the German CAO/ARO/AIO-94 randomized phase III trial after a median follow-up of 11 years. J. Clin. Oncol..

[58] Fokas E., Liersch T., Fietkau R., Hohenberger W., Beissbarth T., Hess C., Becker H., Ghadimi M., Mrak K., Merkel S. (2014). Tumor regression grading after preoperative chemoradiotherapy for locally advanced rectal carcinoma revisited: Updated results of the CAO/ARO/AIO-94 trial. J. Clin. Oncol..

[59] O’Connell M.J., Colangelo L.H., Beart R.W., Petrelli N.J., Allegra C.J., Sharif S., Pitot H.C., Shields A.F., Landry J.C., Ryan D.P. (2014). Capecitabine and oxaliplatin in the preoperative multimodality treatment of rectal cancer: Surgical end points from national surgical adjuvant breast and bowel project trial R-04. J. Clin. Oncol..

[60] Deng Y., Chi P., Lan P., Wang L., Chen W., Cui L., Chen D., Cao J., Wei H., Peng X. (2016). Modified FOLFOX6 with or without Radiation Versus Fluorouracil and Leucovorin with Radiation in Neoadjuvant Treatment of Locally Advanced Rectal Cancer: Initial Results of the Chinese FOWARC Multicenter, Open-Label, Randomized Three-Arm Phase III Trial. J. Clin. Oncol..

[61] Lefevre J.H., Mineur L., Kotti S., Rullier E., Rouanet P., de Chaisemartin C., Meunier B., Mehrdad J., Cotte E., Desrame J. (2016). Effect of Interval (7 or 11 weeks) Between Neoadjuvant Radiochemotherapy and Surgery on Complete Pathologic Response in Rectal Cancer: A Multicenter, Randomized, Controlled Trial (GRECCAR-6). J. Clin. Oncol..

[62] Fokas E., Allgauer M., Polat B., Klautke G., Grabenbauer G.G., Fietkau R., Kuhnt T., Staib L., Brunner T., Grosu A.L. (2019). Randomized phase II trial of chemoradiotherapy plus induction or consolidation chemotherapy as total neoadjuvant therapy for locally advanced rectal cancer: CAO/ARO/AIO-12. J. Clin. Oncol..

[63] Hong Y.S., Kim S.Y., Lee J.S., Nam B.H., Kim K.P., Kim J.E., Park Y.S., Park J.O., Baek J.Y., Kim T.Y. (2019). Oxaliplatin-Based Adjuvant Chemotherapy for Rectal Cancer after Preoperative Chemoradiotherapy (ADORE): Long-Term Results of a Randomized Controlled Trial. J. Clin. Oncol..

[64] Schmoll H.J., Stein A., Van Cutsem E., Price T., Hofheinz R.D., Nordlinger B., Daisne J.F., Janssens J., Brenner B., Reinel H. (2021). Pre- and Postoperative Capecitabine without or with Oxaliplatin in Locally Advanced Rectal Cancer: PETACC 6 Trial by EORTC GITCG and ROG, AIO, AGITG, BGDO, and FFCD. J. Clin. Oncol..

[65] Zhu J., Liu A., Sun X., Liu L., Zhu Y., Zhang T., Jia J., Tan S., Wu J., Wang X. (2020). Multicenter, Randomized, Phase III Trial of Neoadjuvant Chemoradiation with Capecitabine and Irinotecan Guided by UGT1A1 Status in Patients with Locally Advanced Rectal Cancer. J. Clin. Oncol..

[66] Jin J., Tang Y., Hu C., Jiang L.M., Jiang J., Li N., Liu W.Y., Chen S.L., Li S., Lu N.N. (2022). Multicenter, Randomized, Phase III Trial of Short-Term Radiotherapy Plus Chemotherapy Versus Long-Term Chemoradiotherapy in Locally Advanced Rectal Cancer (STELLAR). J. Clin. Oncol..

[67] Hofheinz R.D., Wenz F., Post S., Matzdorff A., Laechelt S., Hartmann J.T., Müller L., Link H., Moehler M., Kettner E. (2012). Chemoradiotherapy with capecitabine versus fluorouracil for locally advanced rectal cancer: A randomised, multicentre, non-inferiority, phase 3 trial. Lancet Oncol..

[68] Bosset J.F., Calais G., Mineur L., Maingon P., Stojanovic-Rundic S., Bensadoun R.J., Bardet E., Beny A., Ollier J.C., Bolla M. (2014). Fluorouracil-based adjuvant chemotherapy after preoperative chemoradiotherapy in rectal cancer: Long-term results of the EORTC 22921 randomised study. Lancet Oncol.

[69] van Gijn W., Marijnen C.A., Nagtegaal I.D., Kranenbarg E.M., Putter H., Wiggers T., Rutten H.J., Pahlman L., Glimelius B., van de Velde C.J. (2011). Preoperative radiotherapy combined with total mesorectal excision for resectable rectal cancer: 12-year follow-up of the multicentre, randomised controlled TME trial. Lancet Oncol..

[70] Rodel C., Liersch T., Becker H., Fietkau R., Hohenberger W., Hothorn T., Graeven U., Arnold D., Lang-Welzenbach M., Raab H.R. (2012). Preoperative chemoradiotherapy and postoperative chemotherapy with fluorouracil and oxaliplatin versus fluorouracil alone in locally advanced rectal cancer: Initial results of the German CAO/ARO/AIO-04 randomised phase 3 trial. Lancet Oncol..

[71] van der Pas M.H., Haglind E., Cuesta M.A., Furst A., Lacy A.M., Hop W.C., Bonjer H.J., Colorectal cancer Laparoscopic or Open Resection II (COLOR II) Study Group (2013). Laparoscopic versus open surgery for rectal cancer (COLOR II): Short-term outcomes of a randomised, phase 3 trial. Lancet Oncol..

[72] Hong Y.S., Nam B.H., Kim K.P., Kim J.E., Park S.J., Park Y.S., Park J.O., Kim S.Y., Kim T.Y., Kim J.H. (2014). Oxaliplatin, fluorouracil, and leucovorin versus fluorouracil and leucovorin as adjuvant chemotherapy for locally advanced rectal cancer after preoperative chemoradiotherapy (ADORE): An open-label, multicentre, phase 2, randomised controlled trial. Lancet Oncol..

[73] Jeong S.Y., Park J.W., Nam B.H., Kim S., Kang S.B., Lim S.B., Choi H.S., Kim D.W., Chang H.J., Kim D.Y. (2014). Open versus laparoscopic surgery for mid-rectal or low-rectal cancer after neoadjuvant chemoradiotherapy (COREAN trial): Survival outcomes of an open-label, non-inferiority, randomised controlled trial. Lancet Oncol..

[74] Rodel C., Graeven U., Fietkau R., Hohenberger W., Hothorn T., Arnold D., Hofheinz R.D., Ghadimi M., Wolff H.A., Lang-Welzenbach M. (2015). Oxaliplatin added to fluorouracil-based preoperative chemoradiotherapy and postoperative chemotherapy of locally advanced rectal cancer (the German CAO/ARO/AIO-04 study): Final results of the multicentre, open-label, randomised, phase 3 trial. Lancet Oncol..

[75] Erlandsson J., Holm T., Pettersson D., Berglund A., Cedermark B., Radu C., Johansson H., Machado M., Hjern F., Hallbook O. (2017). Optimal fractionation of preoperative radiotherapy and timing to surgery for rectal cancer (Stockholm III): A multicentre, randomised, non-blinded, phase 3, non-inferiority trial. Lancet Oncol..

[76] Bahadoer R.R., Dijkstra E.A., van Etten B., Marijnen C.A.M., Putter H., Kranenbarg E.M., Roodvoets A.G.H., Nagtegaal I.D., Beets-Tan R.G.H., Blomqvist L.K. (2021). Short-course radiotherapy followed by chemotherapy before total mesorectal excision (TME) versus preoperative chemoradiotherapy, TME, and optional adjuvant chemotherapy in locally advanced rectal cancer (RAPIDO): A randomised, open-label, phase 3 trial. Lancet Oncol..

[77] Conroy T., Bosset J.F., Etienne P.L., Rio E., Francois E., Mesgouez-Nebout N., Vendrely V., Artignan X., Bouche O., Gargot D. (2021). Neoadjuvant chemotherapy with FOLFIRINOX and preoperative chemoradiotherapy for patients with locally advanced rectal cancer (UNICANCER-PRODIGE 23): A multicentre, randomised, open-label, phase 3 trial. Lancet Oncol..

[78] Fountzilas G., Zisiadis A., Dafni U., Konstantaras C., Hatzitheoharis G., Liaros A., Athanassiou E., Dombros N., Dervenis C., Basdanis G. (1999). Postoperative radiation and concomitant bolus fluorouracil with or without additional chemotherapy with fluorouracil and high-dose leucovorin in patients with high-risk rectal cancer: A randomized phase III study conducted by the Hellenic Cooperative Oncology Group. Ann. Oncol..

[79] Fernandez-Martos C., Garcia-Albeniz X., Pericay C., Maurel J., Aparicio J., Montagut C., Safont M.J., Salud A., Vera R., Massuti B. (2015). Chemoradiation, surgery and adjuvant chemotherapy versus induction chemotherapy followed by chemoradiation and surgery: Long-term results of the Spanish GCR-3 phase II randomized trialdagger. Ann. Oncol..

[80] Gennatas C., Dardoufas C., Mouratidou D., Tsavaris N., Pouli A., Androulakis G., Philippakis M., Voros D., Batalis T., Besbeas S. (2003). Surgical adjuvant therapy of rectal carcinoma: A controlled evaluation of leucovorin, 5-fluorouracil and radiation therapy with or without interferon-alpha2b. Ann. Oncol..

[81] Breugom A.J., van Gijn W., Muller E.W., Berglund A., van den Broek C.B.M., Fokstuen T., Gelderblom H., Kapiteijn E., Leer J.W.H., Marijnen C.A.M. (2015). Adjuvant chemotherapy for rectal cancer patients treated with preoperative (chemo)radiotherapy and total mesorectal excision: A Dutch Colorectal Cancer Group (DCCG) randomized phase III trial. Ann. Oncol..

[82] Maréchal R., Vos B., Polus M., Delaunoit T., Peeters M., Demetter P., Hendlisz A., Demols A., Franchimont D., Verset G. (2012). Short course chemotherapy followed by concomitant chemoradiotherapy and surgery in locally advanced rectal cancer: A randomized multicentric phase II study. Ann. Oncol..

[83] Helbling D., Bodoky G., Gautschi O., Sun H., Bosman F., Gloor B., Burkhard R., Winterhalder R., Madlung A., Rauch D. (2013). Neoadjuvant chemoradiotherapy with or without panitumumab in patients with wild-type KRAS, locally advanced rectal cancer (LARC): A randomized, multicenter, phase II trial SAKK 41/07. Ann. Oncol..

[84] Sclafani F., Roy A., Cunningham D., Wotherspoon A., Peckitt C., de Castro D.G., Tabernero J., Glimelius B., Cervantes A., Eltahir Z. (2013). HER2 in high-risk rectal cancer patients treated in EXPERT-C, a randomized phase II trial of neoadjuvant capecitabine and oxaliplatin (CAPOX) and chemoradiotherapy (CRT) with or without cetuximab. Ann. Oncol..

[85] Borg C., Andre T., Mantion G., Boudghene F., Mornex F., Maingon P., Adenis A., Azria D., Piutti M., Morsli O. (2014). Pathological response and safety of two neoadjuvant strategies with bevacizumab in MRI-defined locally advanced T3 resectable rectal cancer: A randomized, noncomparative phase II study. Ann. Oncol..

[86] Glynne-Jones R., Counsell N., Quirke P., Mortensen N., Maraveyas A., Meadows H.M., Ledermann J., Sebag-Montefiore D. (2014). Chronicle: Results of a randomised phase III trial in locally advanced rectal cancer after neoadjuvant chemoradiation randomising postoperative adjuvant capecitabine plus oxaliplatin (XELOX) versus control. Ann. Oncol..

[87] Delbaldo C., Ychou M., Zawadi A., Douillard J.Y., Andre T., Guerin-Meyer V., Rougier P., Dupuis O., Faroux R., Jouhaud A. (2015). Postoperative irinotecan in resected stage II-III rectal cancer: Final analysis of the French R98 Intergroup trialdagger. Ann. Oncol..

[88] Sclafani F., Chau I., Cunningham D., Peckitt C., Lampis A., Hahne J.C., Braconi C., Tabernero J., Glimelius B., Cervantes A. (2015). Prognostic role of the LCS6 KRAS variant in locally advanced rectal cancer: Results of the EXPERT-C trial. Ann. Oncol..

[89] Bujko K., Wyrwicz L., Rutkowski A., Malinowska M., Pietrzak L., Krynski J., Michalski W., Oledzki J., Kusnierz J., Zajac L. (2016). Long-course oxaliplatin-based preoperative chemoradiation versus 5 × 5 Gy and consolidation chemotherapy for cT4 or fixed cT3 rectal cancer: Results of a randomized phase III study. Ann. Oncol..

[90] Rosati G., Ambrosini G., Barni S., Andreoni B., Corradini G., Luchena G., Daniele B., Gaion F., Oliverio G., Duro M. (2016). A randomized trial of intensive versus minimal surveillance of patients with resected Dukes B2-C colorectal carcinoma. Ann. Oncol..

[91] Azria D., Doyen J., Jarlier M., Martel-Lafay I., Hennequin C., Etienne P., Vendrely V., François E., de La Roche G., Bouché O. (2017). Late toxicities and clinical outcome at 5 years of the ACCORD 12/0405-PRODIGE 02 trial comparing two neoadjuvant chemoradiotherapy regimens for intermediate-risk rectal cancer. Ann. Oncol..

[92] Fokas E., Fietkau R., Hartmann A., Hohenberger W., Grutzmann R., Ghadimi M., Liersch T., Strobel P., Grabenbauer G.G., Graeven U. (2018). Neoadjuvant rectal score as individual-level surrogate for disease-free survival in rectal cancer in the CAO/ARO/AIO-04 randomized phase III trial. Ann. Oncol..

[93] Hofheinz R.D., Arnold D., Fokas E., Kaufmann M., Hothorn T., Folprecht G., Fietkau R., Hohenberger W., Ghadimi M., Liersch T. (2018). Impact of age on the efficacy of oxaliplatin in the preoperative chemoradiotherapy and adjuvant chemotherapy of rectal cancer: A post hoc analysis of the CAO/ARO/AIO-04 phase III trial. Ann. Oncol..

[94] Cisel B., Pietrzak L., Michalski W., Wyrwicz L., Rutkowski A., Kosakowska E., Cencelewicz A., Spalek M., Polkowski W., Jankiewicz M. (2019). Long-course preoperative chemoradiation versus 5 × 5 Gy and consolidation chemotherapy for clinical T4 and fixed clinical T3 rectal cancer: Long-term results of the randomized Polish II study. Ann. Oncol..

[95] Diefenhardt M., Ludmir E.B., Hofheinz R.D., Ghadimi M., Minsky B.D., Rödel C., Fokas E. (2020). Association of Treatment Adherence with Oncologic Outcomes for Patients with Rectal Cancer: A Post Hoc Analysis of the CAO/ARO/AIO-04 Phase 3 Randomized Clinical Trial. JAMA Oncol..

[96] Rahma O.E., Yothers G., Hong T.S., Russell M.M., You Y.N., Parker W., Jacobs S.A., Colangelo L.H., Lucas P.C., Gollub M.J. (2021). Use of Total Neoadjuvant Therapy for Locally Advanced Rectal Cancer: Initial Results from the Pembrolizumab Arm of a Phase 2 Randomized Clinical Trial. JAMA Oncol..

[97] Fokas E., Schlenska-Lange A., Polat B., Klautke G., Grabenbauer G.G., Fietkau R., Kuhnt T., Staib L., Brunner T., Grosu A.L. (2022). Chemoradiotherapy plus induction or consolidation chemotherapy as total neoadjuvant therapy for patients with locally advanced rectal cancer: Long-term results of the CAO/ARO/AIO-12 randomized clinical trial. JAMA Oncol..

[98] Kitz J., Fokas E., Beissbarth T., Strobel P., Wittekind C., Hartmann A., Ruschoff J., Papadopoulos T., Rosler E., Ortloff-Kittredge P. (2018). Association of plane of total mesorectal excision with prognosis of rectal cancer: Secondary analysis of the CAO/ARO/AIO-04 phase 3 randomized clinical trial. JAMA Surg..

[99] Zhao S., Zhang L., Gao F., Wu M., Zheng J., Bai L., Li F., Liu B., Pan Z., Liu J. (2021). Transanal Drainage Tube Use for Preventing Anastomotic Leakage after Laparoscopic Low Anterior Resection in Patients with Rectal Cancer: A Randomized Clinical Trial. JAMA Surg..

[100] Fisher B., Wolmark N., Rockette H., Redmond C., Deutsch M., Wickerham D.L., Fisher E.R., Caplan R., Jones J., Lerner H. (1988). Postoperative adjuvant chemotherapy or radiation therapy for rectal cancer: Results from NSABP protocol R-01. J. Natl. Cancer Inst..

[101] Gelber R.D., Goldhirsch A., Cole B.F., Wieand H.S., Schroeder G., Krook J.E. (1996). A quality-adjusted time without symptoms or toxicity (Q-TWiST) analysis of adjuvant radiation therapy and chemotherapy for resectable rectal cancer. J. Natl. Cancer Inst..

[102] Wolmark N., Wieand H.S., Hyams D.M., Colangelo L., Dimitrov N.V., Romond E.H., Wexler M., Prager D., Cruz A.B., Gordon P.H. (2000). Randomized trial of postoperative adjuvant chemotherapy with or without radiotherapy for carcinoma of the rectum: National surgical adjuvant breast and bowel project protocol R-02. J. Natl. Cancer Inst..

[103] Allegra C.J., Yothers G., O’Connell M.J., Beart R.W., Wozniak T.F., Pitot H.C., Shields A.F., Landry J.C., Ryan D.P., Arora A. (2015). Neoadjuvant 5-FU or capecitabine plus radiation with or without oxaliplatin in rectal cancer patients: A phase III randomized clinical trial. J. Natl. Cancer Inst..

[104] Fokas E., Strobel P., Fietkau R., Ghadimi M., Liersch T., Grabenbauer G.G., Hartmann A., Kaufmann M., Sauer R., Graeven U. (2017). Tumor regression grading after preoperative chemoradiotherapy as a prognostic factor and individual-level surrogate for disease-free survival in rectal cancer. J. Natl. Cancer Inst..

[105] Påhlman L., Glimelius B. (1990). Pre- or postoperative radiotherapy in rectal and rectosigmoid carcinoma. Report from a randomized multicenter trial. Ann. Surg..

[106] Sause W.T., Pajak T.F., Noyes R.D., Dobelbower R., Fischbach J., Doggett S., Mohiuddin M. (1994). Evaluation of preoperative radiation therapy in operable colorectal cancer. Ann. Surg..

[107] Gérard A., Buyse M., Nordlinger B., Loygue J., Pène F., Kempf P., Bosset J.F., Gignoux M., Arnaud J.P., Desaive C. (1988). Preoperative radiotherapy as adjuvant treatment in rectal cancer. Final results of a randomized study of the European Organization for Research and Treatment of Cancer (EORTC). Ann. Surg..

[108] den Dulk M., Marijnen C.A., Putter H., Rutten H.J., Beets G.L., Wiggers T., Nagtegaal I.D., van de Velde C.J. (2007). Risk factors for adverse outcome in patients with rectal cancer treated with an abdominoperineal resection in the total mesorectal excision trial. Ann. Surg..

[109] Matthiessen P., Hallböök O., Rutegård J., Simert G., Sjödahl R. (2007). Defunctioning stoma reduces symptomatic anastomotic leakage after low anterior resection of the rectum for cancer: A randomized multicenter trial. Ann. Surg..

[110] Peeters K.C., Marijnen C.A., Nagtegaal I.D., Kranenbarg E.K., Putter H., Wiggers T., Rutten H., Pahlman L., Glimelius B., Leer J.W. (2007). The TME trial after a median follow-up of 6 years: Increased local control but no survival benefit in irradiated patients with resectable rectal carcinoma. Ann. Surg..

[111] Boelens P.G., Heesakkers F.F., Luyer M.D., van Barneveld K.W., de Hingh I.H., Nieuwenhuijzen G.A., Roos A.N., Rutten H.J. (2014). Reduction of postoperative ileus by early enteral nutrition in patients undergoing major rectal surgery: Prospective, randomized, controlled trial. Ann. Surg..

[112] Ansari N., Solomon M.J., Fisher R.J., Mackay J., Burmeister B., Ackland S., Heriot A., Joseph D., McLachlan S.A., McClure B. (2017). Acute adverse events and postoperative complications in a randomized trial of preoperative short-course radiotherapy versus long-course chemoradiotherapy for T3 adenocarcinoma of the rectum: Trans-Tasman radiation oncology group trial (TROG 01.04). Ann. Surg..

[113] Musters G.D., Klaver C.E.L., Bosker R.J.I., Burger J.W.A., van Duijvendijk P., van Etten B., van Geloven A.A.W., de Graaf E.J.R., Hoff C., Leijtens J.W.A. (2017). Biological Mesh Closure of the Pelvic Floor after Extralevator Abdominoperineal Resection for Rectal Cancer: A Multicenter Randomized Controlled Trial (the BIOPEX-Study). Ann. Surg..

[114] Lefevre J.H., Mineur L., Cachanado M., Denost Q., Rouanet P., de Chaisemartin C., Meunier B., Mehrdad J., Cotte E., Desrame J. (2019). Does a longer waiting period after neoadjuvant radio-chemotherapy improve the oncological prognosis of rectal cancer? Three years’ follow-up results of the greccar-6 randomized multicenter trial. Ann. Surg..

[115] Deng X., Liu P., Jiang D., Wei M., Wang X., Yang X., Zhang Y., Wu B., Liu Y., Qiu M. (2020). Neoadjuvant radiotherapy versus surgery alone for stage II/III mid-low rectal cancer with or without high-risk factors: A prospective multicenter stratified randomized trial. Ann. Surg..

[116] Blok R.D., Sharabiany S., Stoker J., Laan E.T.M., Bosker R.J.I., Burger J.W.A., Chaudhri S., van Duijvendijk P., van Etten B., van Geloven A.A.W. (2022). Cumulative 5-year Results of a Randomized Controlled Trial Comparing Biological Mesh with Primary Perineal Wound Closure after Extralevator Abdominoperineal Resection (BIOPEX-Study). Ann. Surg..

[117] Edler D., Hallström M., Johnston P.G., Magnusson I., Ragnhammar P., Blomgren H. (2000). Thymidylate synthase expression: An independent prognostic factor for local recurrence, distant metastasis, disease-free and overall survival in rectal cancer. Clin. Cancer Res..

[118] de Bruin E.C., van de Velde C.J., van de Pas S., Nagtegaal I.D., van Krieken J.H., Gosens M.J., Peltenburg L.T., Medema J.P., Marijnen C.A. (2006). Prognostic value of apoptosis in rectal cancer patients of the dutch total mesorectal excision trial: Radiotherapy is redundant in intrinsically high-apoptotic tumors. Clin. Cancer Res..

[119] de Heer P., Gosens M.J., de Bruin E.C., Dekker-Ensink N.G., Putter H., Marijnen C.A., van den Brule A.J., van Krieken J.H., Rutten H.J., Kuppen P.J. (2007). Cyclooxygenase 2 expression in rectal cancer is of prognostic significance in patients receiving preoperative radiotherapy. Clin. Cancer Res..

[120] Sprenger T., Rödel F., Beissbarth T., Conradi L.C., Rothe H., Homayounfar K., Wolff H.A., Ghadimi B.M., Yildirim M., Becker H. (2011). Failure of downregulation of survivin following neoadjuvant radiochemotherapy in rectal cancer is associated with distant metastases and shortened survival. Clin. Cancer Res..

[121] Wang F., Fan W., Peng J., Lu Z., Pan Z., Li L., Gao Y., Li H., Chen G., Wu X. (2018). Total mesorectal excision with or without preoperative chemoradiotherapy for resectable mid/low rectal cancer: A long-term analysis of a prospective, single-center, randomized trial. Cancer Commun..

[122] Foster J.D., Ewings P., Falk S., Cooper E.J., Roach H., West N.P., Williams-Yesson B.A., Hanna G.B., Francis N.K., Starrcat Investigators (2016). Surgical timing after chemoradiotherapy for rectal cancer, analysis of technique (STARRCAT): Results of a feasibility multi-centre randomized controlled trial. Tech. Coloproctol..

[123] Moore J., Price T., Carruthers S., Selva-Nayagam S., Luck A., Thomas M., Hewett P. (2017). Prospective randomized trial of neoadjuvant chemotherapy during the ‘wait period’ following preoperative chemoradiotherapy for rectal cancer: Results of the WAIT trial. Color. Dis..

[124] Akgun E., Caliskan C., Bozbiyik O., Yoldas T., Sezak M., Ozkok S., Kose T., Karabulut B., Harman M., Ozutemiz O. (2018). Randomized clinical trial of short or long interval between neoadjuvant chemoradiotherapy and surgery for rectal cancer. Br. J. Surg..

[125] Rutkowski A., Pietrzak L., Krynski J., Zajac L., Bednarczyk M., Olesinski T., Szpakowski M., Saramak P., Pierzankowski I., Hevelke P. (2018). The gentamicin-collagen implant and the risk of distant metastases of rectal cancer following short-course radiotherapy and curative resection: The long-term outcomes of a randomized study. Int. J. Color. Dis..

[126] Bujko K., Nasierowska-Guttmejer A., Wyrwicz L., Malinowska M., Krynski J., Kosakowska E., Rutkowski A., Pietrzak L., Kepka L., Radziszewski J. (2013). Neoadjuvant treatment for unresectable rectal cancer: An interim analysis of a multicentre randomized study. Radiother. Oncol..

[127] Seshadri R.A., West N.P., Sundersingh S. (2017). A pilot randomized study comparing extralevator with conventional abdominoperineal excision for low rectal cancer after neoadjuvant chemoradiation. Color. Dis..

[128] Diefenhardt M., Ludmir E.B., Hofheinz R.D., Ghadimi M., Minsky B.D., Fleischmann M., Fokas E., Rödel C. (2021). Impact of body-mass index on treatment and outcome in locally advanced rectal cancer: A secondary, post-hoc analysis of the CAO/ARO/AIO-04 randomized phase III trial. Radiother. Oncol..

[129] Kapiteijn E., Kranenbarg E.K., Steup W.H., Taat C.W., Rutten H.J., Wiggers T., van Krieken J.H., Hermans J., Leer J.W., van de Velde C.J. (1999). Total mesorectal excision (TME) with or without preoperative radiotherapy in the treatment of primary rectal cancer. Prospective randomised trial with standard operative and histopathological techniques. Dutch ColoRectal Cancer Group. Eur. J. Surg..

[130] Masaki T., Matsuoka H., Kobayashi T., Abe N., Takayama M., Tonari A., Sugiyama M., Atomi Y. (2010). Quality assurance of pelvic autonomic nerve-preserving surgery for advanced lower rectal cancer—Preliminary results of a randomized controlled trial. Langenbecks Arch. Surg..

[131] Rullier A., Gourgou-Bourgade S., Jarlier M., Bibeau F., Chassagne-Clément C., Hennequin C., Tisseau L., Leroux A., Ettore F., Peoc’h M. (2013). Predictive factors of positive circumferential resection margin after radiochemotherapy for rectal cancer: The French randomised trial ACCORD12/0405 PRODIGE 2. Eur. J. Cancer.

[132] von den Grun J.M., Hartmann A., Fietkau R., Ghadimi M., Liersch T., Hohenberger W., Weitz J., Sauer R., Wittekind C., Strobel P. (2018). Can clinicopathological parameters predict for lymph node metastases in ypT0-2 rectal carcinoma? Results of the CAO/ARO/AIO-94 and CAO/ARO/AIO-04 phase 3 trials. Radiother. Oncol..

[133] Gérard J.P., Chamorey E., Gourgou-Bourgade S., Benezery K., de Laroche G., Mahé M.A., Boige V., Juzyna B. (2015). Clinical complete response (cCR) after neoadjuvant chemoradiotherapy and conservative treatment in rectal cancer. Findings from the ACCORD 12/PRODIGE 2 randomized trial. Radiother. Oncol..

[134] Donat S.M. (2007). Standards for surgical complication reporting in urologic oncology: Time for a change. Urology.

[135] Quirke P., Durdey P., Dixon M.F., Williams N.S. (1986). Local recurrence of rectal adenocarcinoma due to inadequate surgical resection. Histopathological study of lateral tumour spread and surgical excision. Lancet.

[136] AWMF Leitlinie Kolorektales Karzinom. http://www.awmf-leitli-nien.de/.

