# Supplementary material for: Quality of Surgical Outcome Reporting in Randomised Clinical Trials of Multimodal Rectal Cancer Treatment: A Systematic Review
Source: Cancers (Basel). 2023 Dec 20;16(1):26. doi: 10.3390/cancers16010026 (PMC10778098; doi:10.3390/cancers16010026)
Supplement: Supplementary file 1 [file cancers-16-00026-s001.zip › cancers-2714258-supplementary.docx]

**Quality of Surgical Outcome Reporting in Randomised Clinical Trials of Multimodal Rectal Cancer Treatment: A Systematic Review**

Joanna Janczak^1^, Kristjan Ukegjini, MD^2^, Stephan Bischofberger, MD^2^ Matthias Turina, MD PhD^3^, Philip C. Müller, MD^4^, Thomas Steffen, MD^2^

^1^Clinic for General and Visceral Surgery, Hospital for the Region Fürstenland Toggenburg, CH-9500 Wil SG, Switzerland

^2^Department of Surgery, Hospital of the Canton of St. Gallen, CH-9007 St. Gallen, Switzerland

^3^Department of Surgery and Transplantation, University Hospital Zurich, Zurich, Switzerland

^4^Department of Surgery, Clarunis – University Centre for Gastrointestinal and Hepatopancreatobiliary Diseases, Basel, Switzerland

**Supplementary Materials - Index**

| **Supplementary Figures and Tables** |  |
| --- | --- |
| **Supplementary Figure S1**  ***Supplementary Figure S2*** | *page 7 lines 223-224*  ***page 10 lines 283-284*** |
| **Supplementary Table S1** | *page 5 line 152* |
| **Supplementary Table S2**  ***Supplementary Table S3*** | *page 6 line 190*  ***page 6 line 190*** |
| ***Supplementary References*** | ***page 6 line 190*** |

**Supplementary Figures and Tables**

**Supplementary Figure S1.** Analysis of mean quality criteria met from 1984 to 2022. The figure demonstrates no significant linear trend between the quality of articles.

*Supplementary Figure S2. Analysis of Procedure-specific quality criteria met from 1984 to 2022. The figure demonstrates no significant linear trend between the quality of articles.*

**Supplementary Table S1.** Jadad scale for assessing the quality of reporting in randomised clinical trials of rectal cancer treatments (modified from Jadad et al.[1])

| **Requirement** | **Points** |
| --- | --- |
| Randomisation |  |
| Study described as randomised. | +1 |
| Randomisation method described and appropriate. | +1 |
| Randomisation method described and inappropriate. | 0 |
| Randomisation method not described. | 0 |
| Blinding |  |
| Study described as double blind (or single blind). | +1 |
| Blinding method described and appropriate. | +1 |
| Blinding method described and inappropriate. | 0 |
| Blinding method not described. | 0 |
| Study not described as blind. | 0 |
| Withdrawals and dropouts |  |
| Withdrawals and dropouts described. | +1 |
| Withdrawals and dropouts not described. | 0 |

Adapted from Jadad et al.[1]

**Supplementary Table S2.** Characteristics and publication output of RCTs of multimodal rectal cancer treatment

| Characteristic | All RCTs, n (%) n = 340 |
| --- | --- |
| Primary purpose |  |
| Treatment | 263 (77.4) |
| Prevention | 16 (4.7) |
| Diagnostic | 8 (2.3) |
| Supportive care | 21 (6.2) |
| Other^a^ | 32 (9.4) |
| Intervention type^a^ |  |
| Drug | 9 (2.6) |
| Procedure | 304 (89.4) |
| Diagnostic test | 14 (4.1) |
| Other | 13 (3.8) |
| Region^a^ |  |
| Africa | 5 (1.5) |
| Australia | 6 (1.7) |
| Europe | 243 (71.3) |
| North America | 45 (13.2) |
| Central and South America | 6 (1.7) |
| Asia and Pacific | 59 (17.3) |
| Middle East | 2 (0.6) |
| Number of institutions |  |
| Single centre | 98 (28.8) |
| Multicentre | 242 (71.2) |
| Number of patients |  |
| 1–49 | 30 (8.8) |
| 50–99 | 48 (14.1) |
| 100–499 | 164 (48.2) |
| 500–999 | 53 (15.6) |
| > 1000 | 45 (13.3) |
| Primary disease^a^ |  |
| UICC stage I | 49 (14.4) |
| UICC stage II | 302 (88.6) |
| UICC stage III | 288 (84.5) |
| UICC stage IV | 16 (4.7) |
| Missing | 20 (5.9) |
| Publication year |  |
| 1980–1989 | 11 (3.2) |
| 1990–1999 | 47 (13.8) |
| 2000–2009 | 88 (25.9) |
| 2010–2019 | 154 (45.3) |
| 2020–2022 | 40 (11.8) |
| Blinding |  |
| None (open label) | 335 (98.5) |
| Single blind | 1 (0.3) |
| Double blind | 4 (1.2) |
| Trial phase |  |
| Phase I | 1 (0.3) |
| Phase II | 50 (14.7) |
| Phase II/III | 5 (1.5) |
| Phase III | 92 (27) |
| Missing | 192 (56.5) |
| Number of citations |  |
| < 50 | 272 (80) |
| 50–99 | 30 (8.8) |
| 100–499 | 35 (10.3) |
| 500–999 | 2 (0.6) |
| > 1000 | 1 (0.3) |
| Number of study arms |  |
| I | 4 (1.2) |
| II | 314 (92.4) |
| III | 11 (3.2) |
| IV | 9 (2.6) |
| V | 1 (0.3) |
| Missing | 1 (0.3) |
| Primary study endpoint^b^ |  |
| Overall survival (OS) | 73 (21.5) |
| Disease-free survival (DFS) | 51 (15) |
| Locoregional recurrence (LRR) | 73 (21.5) |
| Pathological complete response (pCR) | 51 (15) |
| Pathological features | 12 (3.5) |
| R0-resection rate | 8 (2.4) |
| Treatment-related toxicity | 24 (7.1) |
| Postoperative morbidity and mortality | 19 (5.6) |
| Functional outcome | 19 (5.6) |
| Quality of life (QoL) | 18 (5.3) |
| Predictive value of different proteins | 17 (5) |
| Radiological features | 4 (1.2) |
| Journal of publication |  |
| New England Journal of Medicine | 7 (2.1) |
| The Lancet | 3 (0.9) |
| Journal of Clinical Oncology | 36 (10.6) |
| The Lancet Oncology | 11 (3.2) |
| Annals of Oncology | 17 (5) |
| Journal of the American Medical Association (JAMA) Oncology | 3 (0.9) |
| Journal of the American Medical Association (JAMA) Surgery | 2 (0.6) |
| Journal of the National Cancer Institute | 5 (1.5) |
| Annals of Surgery | 12 (3.5) |
| Clinical Cancer Research | 4 (1.2) |
| Cancer Communications | 1 (0.3) |
| European Journal of Cancer | 13 (3.8) |
| Journal of the American Medical Association (JAMA) Network Open | 1 (0.3) |
| eBioMedicine | 1 (0.3) |
| The Journal of Pathology | 1 (0.3) |
| British Journal of Cancer | 1 (0.3) |
| International Journal of Cancer | 1 (0.3) |
| Clinical Nutrition | 1 (0.3) |
| International Journal of Radiation Oncology*Biology*Physics | 27 (7.9) |
| British Journal of Surgery | 34 (10) |
| Cancers | 11 (3.2) |
| The American Journal of Surgical Pathology | 1 (0.3) |
| Radiotherapy and Oncology | 29 (8.5) |
| World Journal of Gastroenterology | 1 (0.3) |
| Annals of Surgical Oncology | 3 (0.9) |
| Carcinogenesis | 1 (0.3) |
| Journal of Magnetic Resonance Imaging | 1 (0.3) |
| Diseases of the Colon & Rectum | 14 (4.1) |
| Clinical Colorectal Cancer | 3 (0.9) |
| BioMed Central Cancer | 5 (1.5) |
| Scientific Reports | 1 (0.3) |
| Clinical Oncology | 1 (0.3) |
| Acta Oncologica | 2 (0.6) |
| Digestive and Liver Disease | 1 (0.3) |
| European Journal of Clinical Nutrition | 1 (0.3) |
| International Journal of Hyperthermia | 1 (0.3) |
| Oncology Reports | 1 (0.3) |
| Colorectal Disease | 9 (2.6) |
| Techniques in Coloproctology | 2 (0.6) |
| Strahlentherapie und Onkologie | 5 (1.5) |
| Supportive Care in Cancer | 1 (0.3) |
| Radiation Oncology | 5 (1.5) |
| Journal of Surgical Oncology | 4 (1.2) |
| Surgical Oncology | 1 (0.3) |
| Langenbeck's Archives of Surgery | 5 (1.5) |
| International Journal of Clinical Oncology | 1 (0.3) |
| British Journal of Surgery open | 1 (0.3) |
| Integrative Cancer Therapies | 1 (0.3) |
| Japanese Journal of Clinical Oncology | 2 (0.6) |
| Medical Science Monitor | 1 (0.3) |
| International Journal of Colorectal Disease | 6 (1.8) |
| The American Journal of Surgery | 1 (0.3) |
| Anticancer Research | 2 (0.6) |
| Scandinavian Journal of Surgery | 1 (0.3) |
| In Vivo | 1 (0.3) |
| Medicine | 1 (0.3) |
| Indian Journal of Cancer | 1 (0.3) |
| Acta Chirurgica Belgica | 2 (0.6) |
| Nutrición Hospitalaria | 1 (0.3) |
| Acta Chirurgica Hungarica | 1 (0.3) |
| American Journal of Clinical Oncology | 2 (0.6) |
| Asian Pacific Journal of Cancer Prevention | 2 (0.6) |
| Australasian RadioIogy | 1 (0.3) |
| Chinese Journal of Cancer | 1 (0.3) |
| Chirurgie | 1 (0.3) |
| Danish Medical Bulletin | 1 (0.3) |
| Drugs under Experimental and Clinical Research | 2 (0.6) |
| Frontiers of Radiation Therapy and Oncology | 1 (0.3) |
| Hepatogastroenterology | 4 (1.2) |
| Journal of the Egyptian National Cancer Institute | 1 (0.3) |
| The Lancet Gastroenterology & Hepatology | 3 (0.9) |
| Medicina | 1 (0.3) |
| Oncologist | 1 (0.3) |
| Oncotarget | 1 (0.3) |
| Recent Results Cancer Research | 1 (0.3) |
| Revista Do Hospital Das Clínicas | 1 (0.3) |
| Surgical Endoscopy and Other Interventional Techniques | 3 (0.9) |
| Web of Science Group journal impact factor |  |
| > 10 | 101 (29.7) |
| 3–9.999 | 194 (57) |
| 1–2.999 | 17 (5) |
| < 1 | 1 (0.3) |
| N/A | 27 (7.9) |

Abbreviations: UICC = Union for International Cancer Control; N/A = not available

Notes:^a^ Percentages may not add up to 100%, as categories are not mutually exclusive. ^b^ Trials could have > 1 therapeutic focus. For analysis, each therapeutic focus was treated as a binary variable.

***Supplementary Table S3. Baseline characteristics of the included studies***

| **Author** | **Published year** | **Study design** | **Number of institutions** | **Number of patients** | **Primary purpose of trial** | **Primary disease** | **Trial phase** |
| --- | --- | --- | --- | --- | --- | --- | --- |
| Duncan W et al.[2] | 1984 | Randomized | Multicenter | 824 | Treatment | UICC Stage I-III | N/A |
| Boulis- Wassif S. et al.[3] | 1984 | Randomized | Multicenter | 247 | Treatment | UICC Stage I-III | N/A |
| Gastrointestinal Tumor Study Group[4] | 1985 | Randomized | Multicenter | 227 | Treatment | UICC Stage II-III | N/A |
| Pahlman L et al.[5] | 1985 | Randomized | Multicenter | 360 | Treatment | UICC Stage II-III | N/A |
| Gerard A et al.[6] | 1985 | Randomized | Multicenter | 410 | Treatment | UICC Stage II-III | III |
| Stockholm Rectal Cancer Study Group[7] | 1987 | Randomized | Multicenter | 694 | Treatment | UICC Stage I-III | N/A |
| Fisher B et al.[8] | 1988 | Randomized | Multicenter | 555 | Treatment | UICC Stage II-III | N/A |
| Thomas PR et al.[9] | 1988 | Randomized | Multicenter | 227 | Treatment | UICC Stage II-III | N/A |
| Gerard A et al.[10] | 1988 | Randomized | Multicenter | 466 | Treatment | UICC Stage II-III | N/A |
| Niebel W et al.[11] | 1988 | Randomized | Multicenter | 142 | Treatment | UICC Stage II-III | N/A |
| Reis Neto JA et al.[12] | 1989 | Randomized | Single center | 68 | Treatment | UICC Stage I-III | N/A |
| Dahl O et al.[13] | 1990 | Randomized | Multicenter | 309 | Treatment | UICC Stage II-III | N/A |
| Stockholm Rectal Cancer Study Group[14] | 1990 | Randomized | Multicenter | 847 | Treatment | UICC Stage I-III | N/A |
| Pahlman L et al.[15] | 1990 | Randomized | Multicenter | 471 | Treatment | UICC Stage I-III | N/A |
| Horn A et al.[16] | 1990 | Randomized | Multicenter | 309 | Treatment | UICC Stage II-III | N/A |
| Krook JE et al.[17] | 1991 | Randomized | Multicenter | 204 | Treatment | UICC Stage II-III | N/A |
| Mameghan H et al.[18] | 1991 | Randomized | Multicenter | 70 | Treatment | UICC Stage II-III | N/A |
| Treurniet-Donker AD et al.[19] | 1991 | Randomized | Multicenter | 172 | Treatment | UICC Stage II-III | N/A |
| Gastrointestinal Tumor Study Group[20] | 1992 | Randomized | Multicenter | 210 | Treatment | UICC Stage II-III | N/A |
| Pahlman L et al.[21] | 1993 | Randomized | Multicenter | 1168 | Treatment | UICC Stage I-III | N/A |
| Hoover jr HC et al.[22] | 1993 | Randomized | Multicenter | 98 | Treatment | UICC Stage II-III | III |
| You QS et al.[23] | 1993 | Randomized | Single center | 146 | Treatment | UICC Stage I-III | N/A |
| Dahl O et al.[24] | 1994 | Randomized | Single center | 161 | Treatment | UICC Stage I-III | N/A |
| Holm T et al.[25] | 1994 | Randomized | Multicenter | 849 | Treatment | UICC Stage I-III | N/A |
| Illényi L et al.[26] | 1994 | Randomized | Single center | 231 | Treatment | UICC Stage II-III | N/A |
| Letschert JG et al.[27] | 1994 | Randomized | Multicenter | 203 | Other | UICC Stage II-III | N/A |
| O`Conell MJ et al.[28] | 1994 | Randomized | Multicenter | 445 | Treatment | UICC Stage I-III | N/A |
| Sause WT et al.[29] | 1994 | Randomized | Multicenter | 70 | Treatment | UICC Stage II-III | N/A |
| Goldberg PA et al.[30] | 1994 | Randomized | Multicenter | 468 | Treatment | UICC Stage I-III | N/A |
| Marsh PJ et al.[31] | 1994 | Randomized | Multicenter | 284 | Treatment | UICC Stage II-III | N/A |
| Cedermark B et al.[32] | 1995 | Randomized | Multicenter | 849 | Treatment | UICC Stage I-III | N/A |
| Marsh PJ et al.[33] | 1995 | Randomized | Multicenter | 284 | Treatment | UICC Stage II-III | N/A |
| Martenson JA et al.[34] | 1995 | Randomized | Single center | 625 | Treatment | UICC Stage II-III | N/A |
| Holm T et al.[35] | 1995 | Randomized | Multicenter | 1292 | Treatment | UICC Stage I-III | N/A |
| Stockholm Colorectal Cancer Study Group[36] | 1996 | Randomized | Multicenter | 577 | Treatment | UICC Stage II-III | N/A |
| Gelber RD et al.[37] | 1996 | Randomized | Multicenter | 204 | Treatment | UICC Stage IIIC | N/A |
| Swedish Rectal Cancer Trial[38] | 1996 | Randomized | Multicenter | 1168 | Treatment | UICC Stage I-III | N/A |
| Susak YM et al.[39] | 1996 | Randomized | Multicenter | 96 | Treatment | UICC Stage I-III | N/A |
| Arnott SJ et al.[40] | 1996 | Randomized | Multicenter | 469 | Treatment | UICC Stage II-III | N/A |
| Holm T et al.[41] | 1996 | Randomized | Multicenter | 1027 | Treatment | UICC Stage II-III | N/A |
| Mohiuddin M et al.[42] | 1996 | Randomized | Multicenter | 297 | Treatment | UICC Stage II-III | N/A |
| Arnaud JP et al.[43] | 1997 | Randomized | Multicenter | 172 | Treatment | UICC Stage II-III | III |
| Bussieres E et al.[44] | 1997 | Randomized | Multicenter | 57 | Treatment | UICC Stage II-III | III |
| Graf W et al.[45] | 1997 | Randomized | Multicenter | 1316 | Treatment | UICC Stage I-III | N/A |
| Holm T et al.[46] | 1997 | Randomized | Multicenter | 849 | Treatment | UICC Stage II-III | N/A |
| Hyams DM et al.[47] | 1997 | Randomized | Single center | 116 | Treatment | UICC Stage II-III | N/A |
| Tepper JE et al.[48] | 1997 | Randomized | Multicenter | 1696 | Treatment | UICC Stage II-III | N/A |
| Tveit KM et al.[49] | 1997 | Randomized | Multicenter | 144 | Treatment | UICC Stage II-III | N/A |
| Cedermark B et al.[50] | 1997 | Randomized | Multicenter | 1168 | Treatment | UICC Stage I-III | N/A |
| Bondar GV et al.[51] | 1998 | Randomized | Single center | 48 | Treatment | UICC Stage II-III | N/A |
| Dahlberg M et al.[52] | 1998 | Randomized | Multicenter | 203 | Other | UICC Stage I-III | N/A |
| Hagmüller E et al.[53] | 1998 | Randomized | Multicenter | 206 | Treatment | UICC Stage II-III | N/A |
| Petersen S et al.[54] | 1998 | Randomized | Single center | 93 | Treatment | UICC Stage II-III | N/A |
| Fountzilas G et al.[55] | 1999 | Randomized | Multicenter | 220 | Treatment | UICC Stage II-III | III |
| Adell G et al.[56] | 1999 | Randomized | Single center | 163 | other | UICC Stage I-IV | N/A |
| Herrmann T et al.[57] | 1999 | Randomized | Single center | 94 | Treatment | UICC Stage I-IV | N/A |
| Kapiteijn E et al.[58] | 1999 | Randomized | Multicenter | 500 | Treatment | UICC Stage II-III | N/A |
| Francois Y et al.[59] | 1999 | Randomized | Multicenter | 201 | Treatment | UICC Stage II-III | N/A |
| Edler D et al.[60] | 2000 | Randomized | Single center | 243 | Other | UICC Stage I-III | N/A |
| Russell AH et al.[61] | 2000 | Randomized | Multicenter | 65 | Treatment | UICC Stage I-III | II |
| Wolmark N et al.[62] | 2000 | Randomized | Multicenter | 400 | Treatment | UICC Stage II-III | N/A |
| Cafiero f et al.[63] | 2000 | Randomized | Single center | 218 | Treatment | UICC Stage II-III | N/A |
| Bosset JF et al.[64] | 2001 | Randomized | Multicenter | 484 | Treatment | UICC Stage II-III | N/A |
| Sauer R et al.[65] | 2001 | Randomized | Multicenter | 628 | Treatment | UICC Stage II-III | III |
| Adell G et al.[66] | 2001 | Randomized | Multicenter | 150 | Treatment | UICC Stage I-III | N/A |
| Frykholm GJ et al.[67] | 2001 | Randomized | Single center | 70 | Treatment | UICC Stage II-III | N/A |
| Holm T et al.[68] | 2001 | Randomized | Multicenter | 457 | Treatment | UICC Stage I-IV | N/A |
| Kapiteijn E et al.[69] | 2001 | Randomized | Multicenter | 1861 | Treatment | UICC Stage II-III | N/A |
| Kim NK et al.[70] | 2001 | Randomized | Single center | 28 | Treatment | UICC Stage III | N/A |
| Marijnen CA et al.[71] | 2001 | Randomized | Multicenter | 1530 | Treatment | UICC Stage I-III | N/A |
| Martling A et al.[72] | 2001 | Randomized | Multicenter | 557 | Treatment | UICC Stage II-III | N/A |
| Tepper JE et al.[73] | 2001 | Randomized | Multicenter | 1664 | Treatment | UICC Stage II-III | N/A |
| Nagawa H et al.[74] | 2001 | Randomized | Single center | 51 | Treatment | UICC Stage I-III | III |
| Lee JH et al.[75] | 2002 | Randomized | Multicenter | 308 | Treatment | UICC Stage II-III | N/A |
| Nagtegaal ID et al.[76] | 2002 | Randomized | Multicenter | 1306 | Other | UICC Stage II-III | N/A |
| Nagtegaal ID et al.[77] | 2002 | Randomized | Multicenter | 180 | Diagnostic | UICC Stage II-III | N/A |
| Nagtegaal ID et al.[78] | 2002 | Randomized | Multicenter | 656 | Treatment | UICC Stage II-III | N/A |
| Sauer R et al.[79] | 2003 | Randomized | Multicenter | 805 | Treatment | UICC Stage II-III | III |
| Araujo S et al.[80] | 2003 | Randomized | Single center | 28 | Treatment | UICC Stage I-III | N/A |
| Gennatas C et al.[81] | 2003 | Randomized | Single center | 207 | Treatment | UICC Stage II-III | N/A |
| Marijnen CA et al.[82] | 2003 | Randomized | Multicenter | 1318 | Treatment | UICC Stage II-III | N/A |
| Cafiero F et al.[83] | 2003 | Randomized | Multicenter | 218 | Treatment | UICC Stage II-III | N/A |
| James RD et al.[84] | 2003 | Randomized | multicenter | 3681 | Treatment | UICC Stage I-IV | N/A |
| Glehen O et al.[85] | 2003 | Randomized | Multicenter | 201 | Prevention | UICC Stage II-III | N/A |
| Tsavaris N et al.[86] | 2004 | Randomized | Single center | 150 | Treatment | UICC Stage II-III | N/A |
| Bosset JF et al.[87] | 2004 | Randomized | Multicenter | 809 | Treatment | UICC Stage II-III | N/A |
| Habr-Gama A et al.[88] | 2004 | Randomized | Single center | 100 | Treatment | UICC Stage I-III | N/A |
| Sauer R et al.[89] | 2004 | Randomized | Multicenter | 823 | Treatment | UICC Stage II-III | N/A |
| van den Brink M et al.[90] | 2004 | Randomized | Multicenter | 96 | Treatment | UICC Stage II-III | N/A |
| Watanabe M et al.[91] | 2004 | Randomized | Multicenter | 669 | Treatment | UICC Stage II-IV | N/A |
| Gerard J-P et al.[92] | 2004 | Randomized | Single center | 88 | Prevention | UICC Stage II-III | N/A |
| Bosset JF et al.[93] | 2005 | Randomized | Multicenter | 1011 | Diagnostic | UICC Stage II-III | N/A |
| Folkesson J et al.[94] | 2005 | Randomized | Multicenter | 1168 | Treatment | UICC Stage I-III | N/A |
| Lezoche E et al.[95] | 2005 | Randomized | Single center | 40 | Treatment | UICC Stage I | N/A |
| Nagtegaal ID et al.[96] | 2005 | Randomized | Multicenter | 1219 | Treatment | UICC Stage I-III | N/A |
| Rödel C et al.[97] | 2005 | Randomized | Multicenter | 385 | Treatment | UICC Stage II-III | N/A |
| Lygidakis NJ et al.[98] | 2005 | Randomized | Multicenter | 210 | Treatment | UICC Stage II-III | N/A |
| Bujko K et al.[99] | 2005 | Randomized | Multicenter | 316 | Prevention | UICC Stage II-III | III |
| Smalley SR et al.[100] | 2006 | Randomized | Multicenter | 1917 | Treatment | UICC Stage II-III | III |
| Taher AN et al.[101] | 2006 | Randomized | Single center | 50 | Treatment | UICC Stage II-III | N/A |
| Akasu T et al.[102] | 2006 | Randomized | Multicenter | 274 | Treatment | UICC Stage III | N/A |
| Brivio F et al.[103] | 2006 | Randomized | Single center | 88 | Treatment | UICC Stage II-III | N/A |
| de Bruin EC et al.[104] | 2006 | Randomized | Multicenter | 1198 | Other | UICC Stage II-III | N/A |
| Hildebrandt B et al.[105] | 2006 | Randomized | Multicenter | 92 | Treatment | UICC Stage II-III | III |
| Mohiuddin M et al.[106] | 2006 | Randomized | Single center | 103 | Treatment | UICC Stage II-III | II |
| Pollack J et al.[107] | 2006 | Randomized | Multicenter | 139 | Other | UICC Stage I-III | II |
| Bosset JF et al.[108] | 2006 | Randomized | Multicenter | 1011 | Treatment | UICC Stage II-III | N/A |
| Bujko K et al.[109] | 2006 | Randomized | Single center | 312 | Prevention | UICC Stage II-III | N/A |
| Fietkau R et al.[110] | 2007 | Randomized | Multicenter | 879 | Treatment | UICC Stage II-III | N/A |
| Collette L et al.[111] | 2007 | Randomized | Multicenter | 785 | Treatment | UICC Stage II | N/A |
| Rhomberg W et al.[112] | 2007 | Randomized | Multicenter | 36 | Treatment | UICC Stage IV | II |
| Bujko K et al.[113] | 2007 | Randomized | Multicenter | 316 | Diagnostic | UICC Stage II-III | N/A |
| de Heer P et al.[114] | 2007 | Randomized | Multicenter | 510 | Prevention | UICC Stage II-III | N/A |
| den Dulk M et al.[115] | 2007 | Randomized | Multicenter | 1412 | Treatment | UICC Stage II-III | N/A |
| den Dulk M et al.[116] | 2007 | Randomized | Multicenter | 884 | Treatment | UICC Stage II-III | N/A |
| Lange MM et al.[117] | 2007 | Randomized | Multicenter | 339 | Other | UICC Stage II-III | N/A |
| Matthiessen P et al.[118] | 2007 | Randomized | Multicenter | 234 | Treatment | UICC Stage II-III | N/A |
| Peeters K et al.[119] | 2007 | Randomized | Multicenter | 1861 | Treatment | UICC Stage I-IV | N/A |
| Pietrzak Let al.[120] | 2007 | Randomized | Multicenter | 516 | Supportive Care | UICC Stage II-III | N/A |
| Rink AD et al.[121] | 2007 | Randomized | Single center | 30 | Treatment | UICC Stage I-III | N/A |
| Kaçar S et al.[122] | 2008 | Randomized | Single center | 51 | Treatment | UICC Stage II-III | N/A |
| Penopoulos V et al.[123] | 2008 | Randomized | Single center | 150 | Treatment | UICC Stage II-III | N/A |
| Kalofonos HP et al.[124] | 2008 | Randomized | Multicenter | 347 | Treatment | UICC Stage II-III | III |
| Masaki T et al.[125] | 2008 | Randomized | Single center | 44 | Treatment | UICC Stage II-III | N/A |
| Birgisson H et al.[126] | 2008 | Randomized | Multicenter | 1147 | Other | UICC Stage II-III | N/A |
| Braendengen M et al.[127] | 2008 | Randomized | Multicenter | 207 | Treatment | UICC Stage II-III | III |
| de la Torre A et al.[128] | 2008 | Randomized | Single center | 155 | Treatment | UICC Stage II-III | N/A |
| Debucquoy A et al.[129] | 2008 | Randomized | Single center | 95 | Other | UICC Stage II-III | N/A |
| Horisberger K et al.[130] | 2008 | Randomized | Single center | 59 | Treatment | UICC Stage II-III | N/A |
| Lange MM et al.[131] | 2008 | Randomized | Multicenter | 785 | Other | UICC Stage II-III | N/A |
| Lezoche G et al.[132] | 2008 | Randomized | Single center | 70 | Treatment | UICC Stage I | N/A |
| Ulrich AB et al.[133] | 2008 | Randomized | Single center | 149 | Treatment | UICC Stage I-III | N/A |
| Valentini V et al.[134] | 2008 | Randomized | Multicenter | 164 | Treatment | UICC Stage II | II |
| Rutkowski A et al.[135] | 2008 | Randomized | Multicenter | 287 | Prevention | UICC Stage II-III | N/A |
| Kaçar S et al.[136] | 2009 | Randomized | Single center | 51 | Treatment | UICC Stage II-III | N/A |
| Roh MS et al.[137] | 2009 | Randomized | Multicenter | 267 | Treatment | UICC Stage II-III | N/A |
| Buunen M et al.[138] | 2009 | Randomized | Multicenter | 1275 | Treatment | UICC Stage II-III | II |
| Debucquoy A et al.[139] | 2009 | Randomized | Single center | 35 | Treatment | UICC Stage II-III | II |
| Fokstuen T et al.[140] | 2009 | Randomized | Multicenter | 274 | Treatment | UICC Stage II-III | N/A |
| Keilholz L et al.[141] | 2009 | Randomized | Single center | 103 | Treatment | UICC Stage II-III | N/A |
| Lange MM et al.[142] | 2009 | Randomized | Multicenter | 990 | Supportive Care | UICC Stage II-III | N/A |
| Lööf J et al.[143] | 2009 | Randomized | Single center | 138 | Preventio | UICC Stage II-III | N/A |
| Lujan J et al.[144] | 2009 | Randomized | Single center | 204 | Treatment | UICC Stage II-III | N/A |
| Matsuoka H et al.[145] | 2009 | Randomized | Single center | 24 | Other | UICC Stage II-III | N/A |
| Parc Y et al.[146] | 2009 | Randomized | Single center | 364 | Other | UICC Stage I-III | N/A |
| Sebag-Montefiore D et al.[147] | 2009 | Randomized | Multicenter | 1350 | Treatment | UICC Stage II-III | N/A |
| Kornmann M et al.[148] | 2010 | Randomized | Single center | 796 | Treatment | UICC Stage II-III | N/A |
| Bujko K et al.[149] | 2010 | Randomized | Single center | 131 | Treatment | UICC Stage II-III | N/A |
| Fernandez-Martos C et al.[150] | 2010 | Randomized | Multicenter | 108 | Treatment | UICC Stage II-III | II |
| Gérard JP et al.[151] | 2010 | Randomized | Multicenter | 598 | Treatment | UICC Stage II-III | III |
| Kusters M et al.[152] | 2010 | Randomized | Multicenter | 1417 | Treatment | UICC Stage IV | N/A |
| Masaki T et al.[153] | 2010 | Randomized | Single center | 58 | Treatment | UICC Stage II-III | N/A |
| Pettersson D et al.[154] | 2010 | Randomized | Multicenter | 303 | Treatment | UICC Stage II-III | N/A |
| Stephens RJ et al.[155] | 2010 | Randomized | Multicenter | 1208 | Supportive Care | UICC Stage II-III | N/A |
| Tiv M et al.[156] | 2010 | Randomized | Multicenter | 207 | Supportive Care | UICC Stage II-III | N/A |
| Tunio MA et al.[157] | 2010 | Randomized | Single center | 36 | Treatment | UICC Stage III | N/A |
| Velenik V et al.[158] | 2010 | Randomized | Single center | 37 | Treatment | UICC Stage II-III | II |
| Velenik V et al.[159] | 2010 | Randomized | Single center | 57 | Treatment | UICC Stage II-III | II |
| Aschele C et al.[160] | 2011 | Randomized | Multicenter | 747 | Treatment | UICC Stage II-III | III |
| Dubois JB et al.[161] | 2011 | Randomized | Multicenter | 142 | Other | UICC Stage II-III | III |
| Fuller CD et al.[162] | 2011 | Randomized | Multicenter | 14 | Diagnostic | UICC Stage I-IV | N/A |
| Kim TW et al.[163] | 2011 | Randomized | Single center | 308 | Treatment | UICC Stage II-III | N/A |
| Kim YC et al.[164] | 2011 | Randomized | Single center | 34 | Treatment | UICC Stage II-III | N/A |
| Nijkamp J et al.[165] | 2011 | Randomized | Multicenter | 1417 | Treatment | UICC Stage II-III | N/A |
| Park JH et al.[166] | 2011 | Randomized | Single center | 220 | Treatment | UICC Stage II-III | III |
| Rotovnik Kozjek N et al.[167] | 2011 | Randomized | Single center | 33 | Other | UICC Stage II-III | N/A |
| Sprenger T et al.[168] | 2011 | Randomized | Single center | 116 | Prevention | UICC Stage II-III | N/A |
| van Gijn W et al.[169] | 2011 | Randomized | Multicenter | 1861 | Treatment | UICC Stage II-III | N/A |
| Wolff HA et al.[170] | 2011 | Randomized | Single center | 22 | Prevention | UICC Stage II-III | N/A |
| Brændengen M et al.[171] | 2011 | Randomized | Multicenter | 207 | Treatment | UICC Sstage II | III |
| Hofheinz RD et al.[172] | 2012 | Randomized | Multicenter | 401 | Treatment | UICC Stage II-III | III |
| Pach R et al.[173] | 2012 | Randomized | Single center | 154 | Treatment | UICC Stage I-III | N/A |
| Salmenkylä S et al.[174] | 2012 | Randomized | Multicenter | 278 | Treatment | UICC Stage II-III | N/A |
| Sauer R et al.[175] | 2012 | Randomized | Multicenter | 799 | Treatment | UICC Stage II-III | III |
| Brændengen M et al.[176] | 2012 | Randomized | Multicenter | 207 | Supportive Care | UICC Stage IV | III |
| Dewdney A et al.[177] | 2012 | Randomized | Multicenter | 254 | Treatment | N/A | II |
| Doeksen A et al.[178] | 2012 | Randomized | Multicenter | 107 | Treatment | UICC Stage II-III | N/A |
| Gérard JP et al.[179] | 2012 | Randomized | Multicenter | 598 | Other | UICC Stage II-III | N/A |
| Jakobsen A et al.[180] | 2012 | Randomized | Multicenter | 248 | Treatment | UICC Stage II | N/A |
| Latkauskas T et al.[181] | 2012 | Randomized | Single center | 83 | Treatment | UICC Stage II-III | N/A |
| Maréchal R et al.[182] | 2012 | Randomized | Single center | 57 | Treatment | UICC Stage II-III | N/A |
| Ngan SY et al.[183] | 2012 | Randomized | Multicenter | 323 | Treatment | UICC Stage III | N/A |
| Niazi TM et al.[184] | 2012 | Randomized | Single center | 42 | Other | UICC Stage II-III | III |
| Ortholan C et al.[185] | 2012 | Randomized | Single center | 88 | Treatment | UICC Stage II | III |
| Rödel C et al.[186] | 2012 | Randomized | Multicenter | 1236 | Treatment | UICC Stage III | III |
| Wong SJ et al.[187] | 2012 | Randomized | Single center | 146 | Treatment | UICC Stage II | II |
| van den Broek CB et al.[188] | 2013 | Randomized | Multicenter | 293 | Treatment | UICC Stage II-III | N/A |
| Bujko K et al.[189] | 2013 | Randomized | Multicenter | 97 | Treatment | UICC Stage III-IV | N/A |
| Engineer R et al.[190] | 2013 | Randomized | Single center | 90 | Treatment | N/A | III |
| Helbling D et al.[191] | 2013 | Randomized | Single center | 68 | Treatment | UICC Stage II-III | II |
| Mohammadzadeh M et al.[192] | 2013 | Randomized | Single center | 34 | Treatment | UICC Stage II-III | N/A |
| Mohiuddin M et al.[193] | 2013 | Randomized | Single center | 106 | Treatment | UICC Stage II | II |
| Pettersson D et al.[194] | 2013 | Randomized | Multicenter | 585 | Treatment | N/A | III |
| Rullier A et al.[195] | 2013 | Randomized | Multicenter | 567 | Treatment | UICC Stage II | III |
| Sclafani F et al.[196] | 2013 | Randomized | Single center | 118 | Prevention | UICC Stage II-III | II |
| van der Pas MH et al.[197] | 2013 | Randomized | Multicenter | 1103 | Treatment | UICC Stage II-III | III |
| Wolff HA et al.[198] | 2013 | Randomized | Multicenter | 654 | Treatment | UICC Stage II-III | III |
| Kotti A et al.[199] | 2013 | Randomized | Single center | 136 | Treatment | N/A | N/A |
| Bosset JF et al.[200] | 2014 | Randomized | Multicenter | 1011 | Treatment | UICC Stage II | III |
| Sainato A et al.[201] | 2014 | Randomized | Multicenter | 645 | Treatment | UICC Stage II | III |
| Andersson J et al.[202] | 2014 | Randomized | Multicenter | 385 | Other | UICC Stage II-III | III |
| Appelt AL et al.[203] | 2014 | Randomized | Multicenter | 221 | Treatment | UICC Stage II | N/A |
| Boelens PG et al.[204] | 2014 | Randomized | Multicenter | 123 | Supportive Care | UICC Stage II-III | NA |
| Borg C et al.[205] | 2014 | Randomized | Multicenter | 91 | Treatment | UICC Stage II | II |
| Borowski DW et al.[206] | 2014 | Randomized | Single center | 100 | Treatment | N/A | N/A |
| Fokas E et al.[207] | 2014 | Randomized | Multicenter | 386 | Treatment | UICC Stage II-III | III |
| François E et al.[208] | 2014 | Randomized | Multicenter | 584 | Treatment | UICC Stage II | III |
| Glynne-Jones R et al.[209] | 2014 | Randomized | Multicenter | 113 | Treatment | UICC Stage III | III |
| Hong YS et al.[210] | 2014 | Randomized | Multicenter | 321 | Treatment | UICC Stage II-III | II |
| Jeong SY et al.[211] | 2014 | Randomized | Multicenter | 340 | Treatment | UICC Stage II-III | III |
| Mavroidis P et al.[212] | 2014 | Randomized | Multicenter | 13 | Diagnostic | UICC Stage II | N/A |
| Ng S et al.[213] | 2014 | Randomized | Single center | 80 | Treatment | UICC Stage II-III | III |
| O`Conell MJ et al.[214] | 2014 | Randomized | Multicenter | 1608 | Treatment | UICC Stage II-III | N/A |
| Rutkowski A et al.[215] | 2014 | Randomized | Single center | 176 | Other | UICC Stage II-III | N/A |
| Sclafani F et al.[216] | 2014 | Randomized | Multicenter | 149 | Treatment | UICC Stage II-III | III |
| Touny A et al.[217] | 2014 | Randomized | Single center | 60 | Other | UICC Stage 0-III | N/A |
| Wiltink LM et al.[218] | 2014 | Randomized | Multicenter | 1530 | Supportive Care | UICC Stage II-III | N/A |
| Fernandez-Martos C et al.[219] | 2015 | Randomized | Multicenter | 108 | Treatment | UICC Stage II-III | II |
| Berugom AJK et al.[220] | 2015 | Randomized | Multicenter | 437 | Treatment | UICC Stage II-III | III |
| Allegra CJ et al.[221] | 2015 | Randomized | Multicenter | 1595 | Prevention | UICC Stage II-III | III |
| Chen TY et al.[222] | 2015 | Randomized | Multicenter | 478 | Supportive Care | UICC Stage 0-IV | N/A |
| Delbaldo C et al.[223] | 2015 | Randomized | Multicenter | 357 | Treatment | UICC Stage II-III | N/A |
| Fan WH et al.[224] | 2015 | Randomized | Multicenter | 184 | Treatment | UICC Stage II-III | II |
| Frøseth TC et al.[225] | 2015 | Randomized | Multicenter | 91 | Treatment | UICC Stage II-III | N/A |
| Gérard JP et al.[226] | 2015 | Randomized | Multicenter | 201 | Treatment | N/A | III |
| Jung M et al.[227] | 2015 | Randomized | Single center | 142 | Treatment | UICC Stage II-III | II |
| Pettersson D et al.[228] | 2015 | Randomized | Multicenter | 462 | Treatment | UICC Stage II-III | III |
| Rödel C et al.[229] | 2015 | Randomized | Multicenter | 1236 | Treatment | UICC Stage II-III | III |
| Salazar R et al.[230] | 2015 | Randomized | Multicenter | 90 | Treatment | UICC Stage II-III | II |
| Sclafani F at al.[231] | 2015 | Randomized | Multicenter | 155 | Prevention | N/A | II |
| Sclafani F et al.[232] | 2015 | Randomized | Multicenter | 164 | Supportive Care | UICC Stage II-III | II |
| Viadl-Casariego A et al.[233] | 2015 | Randomized | Single center | 10 | Other | N/A | N/A |
| Ansari N et al.[234] | 2015 | Randomized | Multicenter | 165 | Other | UICC Stage II-III | N/A |
| Bujko K et al.[235] | 2016 | Randomized | Multicenter | 515 | Treatment | UICC Stage II-III | III |
| Cho H et al.[236] | 2016 | Randomized | Single center | 38 | Treatment | UICC Stage II-III | II |
| Deng Y et al.[237] | 2016 | Randomized | Multicenter | 495 | Treatment | UICC Stage II-III | III |
| Feng YR et al.[238] | 2016 | Randomized | Multicenter | 492 | Treatment | UICC Stage II-III | III |
| Garrer WY et al.[239] | 2016 | Randomized | Single center | 52 | Prevention | UICC Stage II-III | NA |
| Huang M et al.[240] | 2016 | Randomized | Multicenter | 102 | Other | UICC Stage II-III | II |
| Kye BH et al.[241] | 2016 | Randomized | Single center | 56 | Other | UICC Stage II-III | NA |
| Latkauskas T et al.[242] | 2016 | Randomized | Single center | 140 | Treatment | UICC Stage II-III | III |
| Lefevre et al.[243] | 2016 | Randomized | Multicenter | 265 | Treatment | UICC Stage II-III | III |
| McLachlan SA et al.[244] | 2016 | Randomized | Multicenter | 297 | Supportive Care | UICC Stage II-III | N/A |
| Nahas SC et al.[245] | 2016 | Randomized | Single center | 118 | Treatment | UICC Stage II-III | N/A |
| Qin Q et al.[246] | 2016 | Randomized | Multicenter | 318 | Other | UICC Stage II-III | II/III |
| Rosati G et al.[247] | 2016 | Randomized | Multicenter | 1228 | Supportive Care | UICC Stage II-III | N/A |
| Saito S et al.[248] | 2016 | Randomized | Multicenter | 701 | Supportive Care | UICC Stage II-III | III |
| Sclafani F et al.[249] | 2016 | Randomized | Multicenter | 155 | Prevention | N/A | II |
| Shi L et al.[250] | 2016 | Randomized | Single center | 57 | Treatment | UICC Stage II-IV | II |
| Wiltink LM et al.[251] | 2016 | Randomized | Multicenter | 478 | Supportive Care | UICC Stage II-III | N/A |
| Wiśniowska K et al.[252] | 2016 | Randomized | Multicenter | 272 | Treatment | UICC Stage II-III | III |
| Bianco F et al.[253] | 2016 | Randomized | Multicenter | 34 | Treatment | UICC Stage II-III | N/A |
| Cotte E et al.[254] | 2016 | Randomized | Multicenter | 210 | Treatment | UICC Stage II-III | N/A |
| Foster JD et al.[255] | 2016 | Randomized | Multicenter | 31 | Other | UICC Stage II-III | N/A |
| Kairevičė L et al.[256] | 2017 | Randomized | Multicenter | 150 | Treatment | UICC Stage II-III | III |
| Ansari N et al.[257] | 2017 | Randomized | Multicenter | 322 | Treatment | UICC Stage II | III |
| Azria D et al.[258] | 2017 | Randomized | Multicenter | 598 | Other | UICC Stage II-III | III |
| Erlandsson J et al.[259] | 2017 | Randomized | Multicenter | 840 | Treatment | UICC Stage II-III | III |
| Fokas E et al.[260] | 2017 | Randomized | Multicenter | 1179 | Treatment | UICC Stage II-III | III |
| Haddad P et al.[261] | 2017 | Randomized | Single center | 63 | Treatment | UICC Stage II-III | N/A |
| Rotovnik Kozjek N et al.[262] | 2017 | Randomized | Single center | 73 | Treatment | UICC Stage II | N/A |
| Moore J et al.[263] | 2017 | Randomized | Single center | 49 | Treatment | UICC Stage II-III | III |
| Musters GD et al.[264] | 2017 | Randomized | Multicenter | 94 | Supportive Care | UICC Stage II-III | III |
| Rouanet P et al.[265] | 2017 | Randomized | Multicenter | 206 | Treatment | UICC Stage II-III | II |
| Rullier E et al.[266] | 2017 | Randomized | Multicenter | 186 | Treatment | UICC Stage II | III |
| Seshadri RA et al.[267] | 2017 | Randomized | Single center | 20 | Treatment | UICC Stage II-III | III |
| Singh K et al.[268] | 2017 | Randomized | Single center | 30 | Treatment | N/A | N/A |
| Rutkowski A et al.[269] | 2018 | Randomized | Single center | 162 | Treatment | UICC Stage II-III | N/A |
| Sprenger T et al.[270] | 2018 | Randomized | Multicenter | 799 | Treatment | N/A | III |
| von den Grün JM et al.[271] | 2018 | Randomized | Multicenter | 776 | Treatment | UICC Stage I | III |
| Akgun E et al.[272] | 2018 | Randomized | Single center | 327 | Treatment | UICC Stage II-III | III |
| Dias AS et al.[273] | 2018 | Randomized | Single center | 130 | Treatment | UICC Stage II-III | N/A |
| Fokas E et al.[274] | 2018 | Randomized | Multicenter | 1191 | Treatment | UICC Stage II-III | III |
| Hofheinz RD et al.[275] | 2018 | Randomized | Multicenter | 1232 | Treatment | N/A | III |
| Kim SY et al.[276] | 2018 | Randomized | Multicenter | 110 | Treatment | UICC Stage II-III | II |
| Okada K et al.[277] | 2018 | Randomized | Single center | 80 | Treatment | UICC Stage II-III | N/A |
| Qi F et al.[278] | 2018 | Randomized | Multicenter | 245 | Treatment | N/A | N/A |
| Sclafani F et al.[279] | 2018 | Randomized | Multicenter | 59 | Prevention | N/A | II |
| Wawok P et al.[280] | 2018 | Randomized | Single center | 51 | Treatment | UICC Stage I-II | III |
| Zhu J et al.[281] | 2018 | Randomized | Single center | 26 | Treatment | UICC Stage II-III | I |
| Hu H et al.[282] | 2018 | Randomized | Multicenter | 146 | Diagnostic | N/A | III |
| Wang F et al.[283] | 2018 | Randomized | Single center | 184 | Treatment | UICC Stage II | II |
| Kitz J et al.[284] | 2018 | Randomized | Multicenter | 1152 | Treatment | UICC Stage II-III | III |
| Fiori E et al.[285] | 2019 | Randomized | Single center | 40 | Treatment | UICC Stage IV | II |
| Diefenhardt M et al.[286] | 2019 | Randomized | Multicenter | 1236 | Treatment | UICC Stage II-III | III |
| Cisel B et al.[287] | 2019 | Randomized | Multicenter | 515 | Treatment | UICC Stage II | II |
| Erlandsson J et al.[288] | 2019 | Randomized | Multicenter | 810 | Treatment | UICC Stage II-III | III |
| Erlandsson J et al.[289] | 2019 | Randomized | Multicenter | 810 | Treatment | UICC Stage II-III | III |
| Fokas E et al.[290] | 2019 | Randomized | Multicenter | 306 | Treatment | UICC Stage II-III | II |
| Nougaret S et al.[291] | 2019 | Randomized | Multicenter | 133 | Diagnostic | N/A | II |
| Moug SJ et al.[292] | 2019 | Randomized | Multicenter | 48 | Supportive Care | UICC Stage II-III | N/A |
| Hong YS et al.[293] | 2019 | Randomized | Multicenter | 321 | Treatment | UICC Stage II-III | II |
| Borg C et al.[294] | 2019 | Randomized | Single center | 91 | Treatment | UICC Stage II-III | II |
| van der Valk M et al.[295] | 2019 | Randomized | Multicenter | 226 | Supportive Care | UICC Stage II-III | III |
| Lefèvre JH et al.[296] | 2019 | Randomized | Multicenter | 265 | Treatment | UICC Stage II-III | III |
| Deng Y et al.[297] | 2019 | Randomized | Multicenter | 495 | Treatment | UICC Stage II-III | III |
| Wang S et al.[298] | 2019 | Randomized | Multicenter | 515 | Other | UICC Stage II | II |
| Wang J et al.[299] | 2019 | Randomized | Multicenter | 120 | Treatment | UICC Stage II-III | II |
| Sun W et al.[300] | 2019 | Randomized | Multicenter | 220 | Other | UICC Stage II-III | III |
| Valentini V et al.[301] | 2019 | Randomized | Multicenter | 534 | Treatment | UICC Stage II-III | III |
| Chakravarthy AB et al.[302] | 2020 | Randomized | Multicenter | 355 | Treatment | UICC Stage II-III | III |
| Rullier E et al.[303] | 2020 | Randomized | Multicenter | 148 | Treatment | UICC Stage II | III |
| Masaki T et al.[304] | 2020 | Randomized | Single center | 79 | Treatment | UICC Stage II-III | N/A |
| Terzi C et al.[305] | 2020 | Randomized | Multicenter | 330 | Treatment | UICC Stage II-III | N/A |
| Deng X et al.[306] | 2020 | Randomized | Multicenter | 401 | Treatment | UICC Stage II-III | II/III |
| van den Ende R et al.[307] | 2020 | Randomized | Multicenter | 30 | Treatment | UICC Stage I-II | II |
| van der Valk MJM et al.[308] | 2020 | Randomized | Multicenter | 901 | Supportive Care | UICC Stage II-III | III |
| Couwenberg AM et al.[309] | 2020 | Randomized | Multicenter | 128 | Treatment | UICC Stage II-III | II |
| Diefenhardt M et al.[310] | 2020 | Randomized | Multicenter | 1233 | Treatment | UICC Stage II-III | III |
| Sprenger T et al.[311] | 2020 | Randomized | Multicenter | 799 | Treatment | UICC Stage II-III | III |
| Zhu J et al.[312] | 2020 | Randomized | Multicenter | 356 | Treatment | UICC Stage II-III | III |
| Masaki T et al.[313] | 2020 | Randomized | Single center | 79 | Other | UICC Stage II-III | N/A |
| Salazar R et al.[314] | 2020 | Randomized | Multicenter | 90 | Treatment | UICC Stage II-III | II |
| Bach SP et al.[315] | 2020 | Randomized | Multicenter | 123 | Treatment | UICC Stage I | N/A |
| Schmoll HJ et al.[316] | 2021 | Randomized | Multicenter | 1068 | Treatment | UICC Stage II-III | III |
| Rouanet P et al.[317] | 2021 | Randomized | Multicenter | 195 | Treatment | N/A | III |
| Monteil J et al.[318] | 2021 | Randomized | Multicenter | 365 | Treatment | UICC Stage II-III | III |
| Garant A et al.[319] | 2021 | Randomized | Multicenter | 180 | Diagnostic | UICC Stage II-III | II |
| Bahadoer RR et al.[320] | 2021 | Randomized | Multicenter | 912 | Treatment | UICC Stage II-III | III |
| Kosmala R et al.[321] | 2021 | Randomized | Multicenter | 1236 | Supportive Care | UICC Stage II-III | III |
| Miwa K et al. [322] | 2021 | Randomized | Single center | 103 | Treatment | UICC Stage IV | II |
| Chakrabarti D et al.[323] | 2021 | Randomized | Single center | 140 | Treatment | UICC Stage II-III | N/A |
| Conroy T et al.[324] | 2021 | Randomized | Multicenter | 461 | Treatment | UICC Stage II-III | III |
| Park JW et al.[325] | 2021 | Randomized | Multicenter | 340 | Treatment | UICC Stage II-III | II |
| Xie Y et al.[326] | 2021 | Randomized | Multicenter | 495 | Treatment | UICC Stage II-III | II/III |
| Morielli AR et al.[327] | 2021 | Randomized | Single center | 36 | Other | UICC Stage II-III | II |
| Rahma EO et al.[328] | 2021 | Randomized | Multicenter | 185 | Other | UICC Stage II-III | II |
| Zhao S et al.[329] | 2021 | Randomized | Multicenter | 560 | Treatment | UICC Stage I | N/A |
| Diefenhardt M et al.[330] | 2021 | Randomized | Multicenter | 1265 | Treatment | N/A | III |
| Pach R et al.[331] | 2021 | Randomized | Single center | 154 | Treatment | UICC Stage II-III | N/A |
| Li N et al.[332] | 2021 | Randomized | Multicenter | 589 | Treatment | UICC Stage II-III | N/A |
| Blok RD et al.[333] | 2022 | Randomized | Multicenter | 104 | Other | N/A | III |
| Verweij M et al.[334] | 2022 | Randomized | Multicenter | 128 | Supportive Care | N/A | II |
| Rouanet P et al.[335] | 2022 | Randomized | Multicenter | 133 | Treatment | UICC Stage II-III | II |
| Fokas E et al.[336] | 2022 | Randomized | Multicenter | 311 | Treatment | UICC Stage II-III | II |
| Erlandsson J et al.[337] | 2022 | Randomized | Multicenter | 840 | Treatment | UICC Stage II-III | III |
| Jin J et al.[338] | 2022 | Randomized | Multicenter | 599 | Treatment | UICC Stage III-IV | III |
| Liu W et al.[339] | 2022 | Randomized | Multicenter | 137 | Treatment | UICC Stage II-III | II/III |
| Dijkstra EA et al.[340] | 2022 | Randomized | Multicenter | 663 | Supportive Care | UICC Stage II-III | III |
| Araujo RO et al.[341] | 2022 | Randomized | Multicenter | 63 | Supportive Care | UICC Stage II-III | II/III |

Abbreviations: UICC = Union for International Cancer Control; N/A = not available; Other = functional outcome or treatment-related toxicity

**Supplementary Reference**

1. Jadad, A.R.; Moore, R.A.; Carroll, D.; Jenkinson, C.; Reynolds, D.J.; Gavaghan, D.J.; McQuay, H.J. Assessing the quality of reports of randomized clinical trials: is blinding necessary? *Control Clin Trials* **1996**, *17*, 1-12, doi:10.1016/0197-2456(95)00134-4.

2. The evaluation of low dose pre-operative X-ray therapy in the management of operable rectal cancer; results of a randomly controlled trial. *The British journal of surgery* **1984**, *71*, 21-25, doi:10.1002/bjs.1800710107.

3. Boulis-Wassif, S.; Gerard, A.; Loygue, J.; Camelot, D.; Buyse, M.; Duez, N. Final results of a randomized trial on the treatment of rectal cancer with preoperative radiotherapy alone or in combination with 5-fluorouracil, followed by radical surgery. Trial of the European Organization on Research and Treatment of Cancer Gastrointestinal Tract Cancer Cooperative Group. *Cancer* **1984**, *53*, 1811-1818, doi:10.1002/1097-0142(19840501)53:9<1811::aid-cncr2820530902>3.0.co;2-h.

4. Prolongation of the disease-free interval in surgically treated rectal carcinoma. *N Engl J Med* **1985**, *312*, 1465-1472, doi:10.1056/nejm198506063122301.

5. Påhlman, L.; Glimelius, B.; Graffman, S. Pre- versus postoperative radiotherapy in rectal carcinoma: an interim report from a randomized multicentre trial. *The British journal of surgery* **1985**, *72*, 961-966, doi:10.1002/bjs.1800721209.

6. Gerard, A.; Berrod, J.L.; Pene, F.; Loygue, J.; Laugier, A.; Bruckner, R.; Camelot, G.; Arnaud, J.P.; Metzger, U.; Buyse, M.; et al. Interim analysis of a phase III study on preoperative radiation therapy in resectable rectal carcinoma. Trial of the Gastrointestinal Tract Cancer Cooperative Group of the European Organization for Research on Treatment of Cancer (EORTC). *Cancer* **1985**, *55*, 2373-2379, doi:10.1002/1097-0142(19850515)55:10<2373::aid-cncr2820551012>3.0.co;2-d.

7. Short-term preoperative radiotherapy for adenocarcinoma of the rectum. An interim analysis of a randomized multicenter trial. Stockholm Rectal Cancer Study Group. *Am J Clin Oncol* **1987**, *10*, 369-375, doi:10.1097/00000421-198710000-00001.

8. Fisher, B.; Wolmark, N.; Rockette, H.; Redmond, C.; Deutsch, M.; Wickerham, D.L.; Fisher, E.R.; Caplan, R.; Jones, J.; Lerner, H.; et al. Postoperative adjuvant chemotherapy or radiation therapy for rectal cancer: results from NSABP protocol R-01. *Journal of the National Cancer Institute* **1988**, *80*, 21-29, doi:10.1093/jnci/80.1.21.

9. Thomas, P.R.; Lindblad, A.S. Adjuvant postoperative radiotherapy and chemotherapy in rectal carcinoma: a review of the Gastrointestinal Tumor Study Group experience. *Radiother Oncol* **1988**, *13*, 245-252, doi:10.1016/0167-8140(88)90219-8.

10. Gérard, A.; Buyse, M.; Nordlinger, B.; Loygue, J.; Pène, F.; Kempf, P.; Bosset, J.F.; Gignoux, M.; Arnaud, J.P.; Desaive, C.; et al. Preoperative radiotherapy as adjuvant treatment in rectal cancer. Final results of a randomized study of the European Organization for Research and Treatment of Cancer (EORTC). *Annals of surgery* **1988**, *208*, 606-614, doi:10.1097/00000658-198811000-00011.

11. Niebel, W.; Schulz, U.; Ried, M.; Erhard, J.; Beersiek, F.; Blöcher, G.; Nier, H.; Halama, H.; Scherer, E.; Zeller, G.; et al. Five-year results of a prospective and randomized study: experience with combined radiotherapy and surgery of primary rectal carcinoma. *Recent Results Cancer Res* **1988**, *110*, 111-113, doi:10.1007/978-3-642-83293-2_16.

12. Reis Neto, J.A.; Quilici, F.A.; Reis, J.A., Jr. A comparison of nonoperative vs. preoperative radiotherapy in rectal carcinoma. A 10-year randomized trial. *Dis Colon Rectum* **1989**, *32*, 702-710, doi:10.1007/bf02555778.

13. Dahl, O.; Horn, A.; Morild, I.; Halvorsen, J.F.; Odland, G.; Reinertsen, S.; Reisaeter, A.; Kavli, H.; Thunold, J. Low-dose preoperative radiation postpones recurrences in operable rectal cancer. Results of a randomized multicenter trial in western Norway. *Cancer* **1990**, *66*, 2286-2294, doi:10.1002/1097-0142(19901201)66:11<2286::aid-cncr2820661106>3.0.co;2-t.

14. Preoperative short-term radiation therapy in operable rectal carcinoma. A prospective randomized trial. Stockholm Rectal Cancer Study Group. *Cancer* **1990**, *66*, 49-55, doi:10.1002/1097-0142(19900701)66:1<49::aid-cncr2820660111>3.0.co;2-1.

15. Påhlman, L.; Glimelius, B. Pre- or postoperative radiotherapy in rectal and rectosigmoid carcinoma. Report from a randomized multicenter trial. *Annals of surgery* **1990**, *211*, 187-195, doi:10.1097/00000658-199002000-00011.

16. Horn, A.; Halvorsen, J.F.; Dahl, O. Preoperative radiotherapy in operable rectal cancer. *Dis Colon Rectum* **1990**, *33*, 823-828, doi:10.1007/bf02051916.

17. Krook, J.E.; Moertel, C.G.; Gunderson, L.L.; Wieand, H.S.; Collins, R.T.; Beart, R.W.; Kubista, T.P.; Poon, M.A.; Meyers, W.C.; Mailliard, J.A.; et al. Effective surgical adjuvant therapy for high-risk rectal carcinoma. *N Engl J Med* **1991**, *324*, 709-715, doi:10.1056/nejm199103143241101.

18. Mameghan, H.; Gray, B.N.; de Zwart, J.; Richer, R.; Burns, I.; Hurley, R.; Ibister, W.H.; Reasbeck, P.; Newstead, G. Adjuvant post-operative radiotherapy in rectal cancer: results from the ANZ Bowel Cancer Trial (Protocol 8202). *Australas Radiol* **1991**, *35*, 61-65, doi:10.1111/j.1440-1673.1991.tb02994.x.

19. Treurniet-Donker, A.D.; van Putten, W.L.; Wereldsma, J.C.; Bruggink, E.D.; Hoogenraad, W.J.; Roukema, J.A.; Snijders-Keilholz, A.; Meijer, W.S.; Meerwaldt, J.H.; Wijnmaalen, A.J.; et al. Postoperative radiation therapy for rectal cancer. An interim analysis of a prospective, randomized multicenter trial in The Netherlands. *Cancer* **1991**, *67*, 2042-2048, doi:10.1002/1097-0142(19910415)67:8<2042::aid-cncr2820670806>3.0.co;2-4.

20. Radiation therapy and fluorouracil with or without semustine for the treatment of patients with surgical adjuvant adenocarcinoma of the rectum. Gastrointestinal Tumor Study Group. *Journal of clinical oncology : official journal of the American Society of Clinical Oncology* **1992**, *10*, 549-557, doi:10.1200/jco.1992.10.4.549.

21. Initial report from a Swedish multicentre study examining the role of preoperative irradiation in the treatment of patients with resectable rectal carcinoma. Swedish Rectal Cancer Trial. *The British journal of surgery* **1993**, *80*, 1333-1336, doi:10.1002/bjs.1800801040.

22. Hoover, H.C., Jr.; Brandhorst, J.S.; Peters, L.C.; Surdyke, M.G.; Takeshita, Y.; Madariaga, J.; Muenz, L.R.; Hanna, M.G., Jr. Adjuvant active specific immunotherapy for human colorectal cancer: 6.5-year median follow-up of a phase III prospectively randomized trial. *Journal of clinical oncology : official journal of the American Society of Clinical Oncology* **1993**, *11*, 390-399, doi:10.1200/jco.1993.11.3.390.

23. You, Q.S.; Wang, R.Z.; Suen, G.Q.; Yan, F.C.; Gao, Y.J.; Cui, S.R.; Zhao, J.H.; Zhao, T.Z.; Ding, L. Combination preoperative radiation and endocavitary hyperthermia for rectal cancer: long-term results of 44 patients. *Int J Hyperthermia* **1993**, *9*, 19-24, doi:10.3109/02656739309061475.

24. Dahl, O.; Horn, A.; Mella, O. Do acute side-effects during radiotherapy predict tumour response in rectal carcinoma? *Acta Oncol* **1994**, *33*, 409-413, doi:10.3109/02841869409098437.

25. Holm, T.; Cedermark, B.; Rutqvist, L.E. Local recurrence of rectal adenocarcinoma after 'curative' surgery with and without preoperative radiotherapy. *The British journal of surgery* **1994**, *81*, 452-455, doi:10.1002/bjs.1800810344.

26. Illényi, L.; Grexa, E.; Gecser, G.; Kött, I. Local recurrence of rectal cancer following preoperative irradiation. *Acta Chir Hung* **1994**, *34*, 333-347.

27. Letschert, J.G.; Lebesque, J.V.; Aleman, B.M.; Bosset, J.F.; Horiot, J.C.; Bartelink, H.; Cionini, L.; Hamers, J.P.; Leer, J.W.; van Glabbeke, M. The volume effect in radiation-related late small bowel complications: results of a clinical study of the EORTC Radiotherapy Cooperative Group in patients treated for rectal carcinoma. *Radiother Oncol* **1994**, *32*, 116-123, doi:10.1016/0167-8140(94)90097-3.

28. O'Connell, M.J.; Martenson, J.A.; Wieand, H.S.; Krook, J.E.; Macdonald, J.S.; Haller, D.G.; Mayer, R.J.; Gunderson, L.L.; Rich, T.A. Improving adjuvant therapy for rectal cancer by combining protracted-infusion fluorouracil with radiation therapy after curative surgery. *N Engl J Med* **1994**, *331*, 502-507, doi:10.1056/nejm199408253310803.

29. Sause, W.T.; Pajak, T.F.; Noyes, R.D.; Dobelbower, R.; Fischbach, J.; Doggett, S.; Mohiuddin, M. Evaluation of preoperative radiation therapy in operable colorectal cancer. *Annals of surgery* **1994**, *220*, 668-675, doi:10.1097/00000658-199411000-00011.

30. Goldberg, P.A.; Nicholls, R.J.; Porter, N.H.; Love, S.; Grimsey, J.E. Long-term results of a randomised trial of short-course low-dose adjuvant pre-operative radiotherapy for rectal cancer: reduction in local treatment failure. *Eur J Cancer* **1994**, *30a*, 1602-1606, doi:10.1016/0959-8049(94)00312-s.

31. Marsh, P.J.; James, R.D.; Schofield, P.F. Adjuvant preoperative radiotherapy for locally advanced rectal carcinoma. Results of a prospective, randomized trial. *Dis Colon Rectum* **1994**, *37*, 1205-1214, doi:10.1007/bf02257783.

32. Cedermark, B.; Johansson, H.; Rutqvist, L.E.; Wilking, N. The Stockholm I trial of preoperative short term radiotherapy in operable rectal carcinoma. A prospective randomized trial. Stockholm Colorectal Cancer Study Group. *Cancer* **1995**, *75*, 2269-2275, doi:10.1002/1097-0142(19950501)75:9<2269::aid-cncr2820750913>3.0.co;2-i.

33. Marsh, P.J.; James, R.D.; Schofield, P.F. Definition of local recurrence after surgery for rectal carcinoma. *The British journal of surgery* **1995**, *82*, 465-468, doi:10.1002/bjs.1800820412.

34. Martenson, J.A., Jr.; Urias, R.; Smalley, S.R.; Coia, L.R.; Tepper, J.E.; Rotman, M.; Rich, T.A.; O'Connell, M.J. Radiation therapy quality control in a clinical trial of adjuvant postoperative treatment for rectal cancer. *Int J Radiat Oncol Biol Phys* **1995**, *32*, 51-55, doi:10.1016/0360-3016(95)00526-5.

35. Holm, T.; Rutqvist, L.E.; Johansson, H.; Cedermark, B. Abdominoperineal resection and anterior resection in the treatment of rectal cancer: results in relation to adjuvant preoperative radiotherapy. *The British journal of surgery* **1995**, *82*, 1213-1216, doi:10.1002/bjs.1800820920.

36. Randomized study on preoperative radiotherapy in rectal carcinoma. Stockholm Colorectal Cancer Study Group. *Annals of surgical oncology* **1996**, *3*, 423-430, doi:10.1007/bf02305759.

37. Gelber, R.D.; Goldhirsch, A.; Cole, B.F.; Wieand, H.S.; Schroeder, G.; Krook, J.E. A quality-adjusted time without symptoms or toxicity (Q-TWiST) analysis of adjuvant radiation therapy and chemotherapy for resectable rectal cancer. *Journal of the National Cancer Institute* **1996**, *88*, 1039-1045, doi:10.1093/jnci/88.15.1039.

38. Local recurrence rate in a randomised multicentre trial of preoperative radiotherapy compared with operation alone in resectable rectal carcinoma. Swedish Rectal Cancer Trial. *Eur J Surg* **1996**, *162*, 397-402.

39. Susak, Y.M.; Zemskov, V.S.; Yaremchuk, O.Y.; Kravchenco, O.B.; Yatsyk, I.M.; Korsh, O.B. Comparison of chemotherapy and X-ray therapy with Ukrain monotherapy for colorectal cancer. *Drugs Exp Clin Res* **1996**, *22*, 115-122.

40. Randomised trial of surgery alone versus surgery followed by radiotherapy for mobile cancer of the rectum. Medical Research Council Rectal Cancer Working Party. *Lancet* **1996**, *348*, 1610-1614.

41. Holm, T.; Singnomklao, T.; Rutqvist, L.E.; Cedermark, B. Adjuvant preoperative radiotherapy in patients with rectal carcinoma. Adverse effects during long term follow-up of two randomized trials. *Cancer* **1996**, *78*, 968-976, doi:10.1002/(sici)1097-0142(19960901)78:5<968::Aid-cncr5>3.0.Co;2-8.

42. Mohiuddin, M.; Regine, W.F.; Marks, G. Prognostic significance of tumor fixation of rectal carcinoma. Implications for adjunctive radiation therapy. *Cancer* **1996**, *78*, 717-722, doi:10.1002/(sici)1097-0142(19960815)78:4<717::Aid-cncr4>3.0.Co;2-b.

43. Arnaud, J.P.; Nordlinger, B.; Bosset, J.F.; Boes, G.H.; Sahmoud, T.; Schlag, P.M.; Pene, F. Radical surgery and postoperative radiotherapy as combined treatment in rectal cancer. Final results of a phase III study of the European Organization for Research and Treatment of Cancer. *The British journal of surgery* **1997**, *84*, 352-357.

44. Bussières, E.; Dubois, J.B.; Demange, L.; Delannes, M.; Richaud, P.; Bécouarn, Y. IORT: a randomized trial in primary rectal cancer by the French group of IORT. *Front Radiat Ther Oncol* **1997**, *31*, 217-220, doi:10.1159/000061186.

45. Graf, W.; Dahlberg, M.; Osman, M.M.; Holmberg, L.; Pählman, L.; Glimelius, B. Short-term preoperative radiotherapy results in down-staging of rectal cancer: a study of 1316 patients. *Radiother Oncol* **1997**, *43*, 133-137, doi:10.1016/s0167-8140(96)01867-1.

46. Holm, T.; Johansson, H.; Cedermark, B.; Ekelund, G.; Rutqvist, L.E. Influence of hospital- and surgeon-related factors on outcome after treatment of rectal cancer with or without preoperative radiotherapy. *The British journal of surgery* **1997**, *84*, 657-663.

47. Hyams, D.M.; Mamounas, E.P.; Petrelli, N.; Rockette, H.; Jones, J.; Wieand, H.S.; Deutsch, M.; Wickerham, L.; Fisher, B.; Wolmark, N. A clinical trial to evaluate the worth of preoperative multimodality therapy in patients with operable carcinoma of the rectum: a progress report of National Surgical Breast and Bowel Project Protocol R-03. *Dis Colon Rectum* **1997**, *40*, 131-139, doi:10.1007/bf02054976.

48. Tepper, J.E.; O'Connell, M.J.; Petroni, G.R.; Hollis, D.; Cooke, E.; Benson, A.B., 3rd; Cummings, B.; Gunderson, L.L.; Macdonald, J.S.; Martenson, J.A. Adjuvant postoperative fluorouracil-modulated chemotherapy combined with pelvic radiation therapy for rectal cancer: initial results of intergroup 0114. *Journal of clinical oncology : official journal of the American Society of Clinical Oncology* **1997**, *15*, 2030-2039, doi:10.1200/jco.1997.15.5.2030.

49. Tveit, K.M.; Guldvog, I.; Hagen, S.; Trondsen, E.; Harbitz, T.; Nygaard, K.; Nilsen, J.B.; Wist, E.; Hannisdal, E. Randomized controlled trial of postoperative radiotherapy and short-term time-scheduled 5-fluorouracil against surgery alone in the treatment of Dukes B and C rectal cancer. Norwegian Adjuvant Rectal Cancer Project Group. *The British journal of surgery* **1997**, *84*, 1130-1135.

50. Cedermark, B.; Dahlberg, M.; Glimelius, B.; Påhlman, L.; Rutqvist, L.E.; Wilking, N. Improved survival with preoperative radiotherapy in resectable rectal cancer. *N Engl J Med* **1997**, *336*, 980-987, doi:10.1056/nejm199704033361402.

51. Bondar, G.V.; Borota, A.V.; Yakovets, Y.I.; Zolotukhin, S.E. Comparative evaluation of the complex treatment of rectal cancer patients (chemotherapy and X-ray therapy, Ukrain monotherapy). *Drugs Exp Clin Res* **1998**, *24*, 221-226.

52. Dahlberg, M.; Glimelius, B.; Graf, W.; Påhlman, L. Preoperative irradiation affects functional results after surgery for rectal cancer: results from a randomized study. *Dis Colon Rectum* **1998**, *41*, 543-549; discussion 549-551, doi:10.1007/bf02235256.

53. Hagmüller, E.; Hartung, G.; Sturm, J.; Diezler, P.; Queisser, W. [Adjuvant radiochemotherapy with 5-FU and folinic acid in Dukes stage B and C rectum carcinoma: an interim analysis]. *Langenbecks Arch Chir Suppl Kongressbd* **1998**, *115*, 1397-1399.

54. Petersen, S.; Hellmich, G.; Baumann, M.; Herrmann, T.; Henke, G.; Ludwig, K. [Brief preoperative radiotherapy in surgical therapy of rectal carcinoma. Long-term results of a prospective randomized study]. *Chirurg* **1998**, *69*, 759-765, doi:10.1007/s001040050487.

55. Fountzilas, G.; Zisiadis, A.; Dafni, U.; Konstantaras, C.; Hatzitheoharis, G.; Liaros, A.; Athanassiou, E.; Dombros, N.; Dervenis, C.; Basdanis, G.; et al. Postoperative radiation and concomitant bolus fluorouracil with or without additional chemotherapy with fluorouracil and high-dose leucovorin in patients with high-risk rectal cancer: a randomized phase III study conducted by the Hellenic Cooperative Oncology Group. *Ann Oncol* **1999**, *10*, 671-676, doi:10.1023/a:1008357609434.

56. Adell, G.; Sun, X.F.; Stål, O.; Klintenberg, C.; Sjödahl, R.; Nordenskjöld, B. p53 status: an indicator for the effect of preoperative radiotherapy of rectal cancer. *Radiother Oncol* **1999**, *51*, 169-174, doi:10.1016/s0167-8140(99)00041-9.

57. Herrmann, T.; Petersen, S.; Hellmich, G.; Baumann, M.; Ludwig, K. [Delayed toxicity of brief preoperative irradiation and risk-adjusted postoperative radiotherapy of operative rectal carcinoma. Results of a randomized prospective study]. *Strahlenther Onkol* **1999**, *175*, 430-436, doi:10.1007/s000660050032.

58. Kapiteijn, E.; Kranenbarg, E.K.; Steup, W.H.; Taat, C.W.; Rutten, H.J.; Wiggers, T.; van Krieken, J.H.; Hermans, J.; Leer, J.W.; van de Velde, C.J. Total mesorectal excision (TME) with or without preoperative radiotherapy in the treatment of primary rectal cancer. Prospective randomised trial with standard operative and histopathological techniques. Dutch ColoRectal Cancer Group. *Eur J Surg* **1999**, *165*, 410-420, doi:10.1080/110241599750006613.

59. Francois, Y.; Nemoz, C.J.; Baulieux, J.; Vignal, J.; Grandjean, J.P.; Partensky, C.; Souquet, J.C.; Adeleine, P.; Gerard, J.P. Influence of the interval between preoperative radiation therapy and surgery on downstaging and on the rate of sphincter-sparing surgery for rectal cancer: the Lyon R90-01 randomized trial. *Journal of clinical oncology : official journal of the American Society of Clinical Oncology* **1999**, *17*, 2396, doi:10.1200/jco.1999.17.8.2396.

60. Edler, D.; Hallström, M.; Johnston, P.G.; Magnusson, I.; Ragnhammar, P.; Blomgren, H. Thymidylate synthase expression: an independent prognostic factor for local recurrence, distant metastasis, disease-free and overall survival in rectal cancer. *Clin Cancer Res* **2000**, *6*, 1378-1384.

61. Russell, A.H.; Harris, J.; Rosenberg, P.J.; Sause, W.T.; Fisher, B.J.; Hoffman, J.P.; Kraybill, W.G.; Byhardt, R.W. Anal sphincter conservation for patients with adenocarcinoma of the distal rectum: long-term results of radiation therapy oncology group protocol 89-02. *Int J Radiat Oncol Biol Phys* **2000**, *46*, 313-322, doi:10.1016/s0360-3016(99)00440-x.

62. Wolmark, N.; Wieand, H.S.; Hyams, D.M.; Colangelo, L.; Dimitrov, N.V.; Romond, E.H.; Wexler, M.; Prager, D.; Cruz, A.B., Jr.; Gordon, P.H.; et al. Randomized trial of postoperative adjuvant chemotherapy with or without radiotherapy for carcinoma of the rectum: National Surgical Adjuvant Breast and Bowel Project Protocol R-02. *Journal of the National Cancer Institute* **2000**, *92*, 388-396, doi:10.1093/jnci/92.5.388.

63. Cafiero, F.; Gipponi, M.; Peressini, A.; Bertoglio, S.; Lionetto, R. Preliminary analysis of a randomized clinical trial of adjuvant postoperative RT vs. postoperative RT plus 5-FU and levamisole in patients with TNM stage II-III resectable rectal cancer. *J Surg Oncol* **2000**, *75*, 80-88, doi:10.1002/1096-9098(200010)75:2<80::aid-jso2>3.0.co;2-o.

64. Bosset, J.F.; Horiot, J.C.; Hamers, H.P.; Cionini, L.; Bartelink, H.; Caspers, R.; Untereiner, M.; Ciambelloti, E.; Pierart, M.; Van Glabbeke, M. Postoperative pelvic radiotherapy with or without elective irradiation of para-aortic nodes and liver in rectal cancer patients. A controlled clinical trial of the EORTC Radiotherapy Group. *Radiother Oncol* **2001**, *61*, 7-13, doi:10.1016/s0167-8140(01)00419-4.

65. Sauer, R.; Fietkau, R.; Wittekind, C.; Martus, P.; Rödel, C.; Hohenberger, W.; Jatzko, G.; Sabitzer, H.; Karstens, J.H.; Becker, H.; et al. Adjuvant versus neoadjuvant radiochemotherapy for locally advanced rectal cancer. A progress report of a phase-III randomized trial (protocol CAO/ARO/AIO-94). *Strahlenther Onkol* **2001**, *177*, 173-181, doi:10.1007/pl00002396.

66. Adell, G.; Zhang, H.; Jansson, A.; Sun, X.F.; Stål, O.; Nordenskjöld, B. Decreased tumor cell proliferation as an indicator of the effect of preoperative radiotherapy of rectal cancer. *Int J Radiat Oncol Biol Phys* **2001**, *50*, 659-663, doi:10.1016/s0360-3016(01)01515-2.

67. Frykholm, G.J.; Påhlman, L.; Glimelius, B. Combined chemo- and radiotherapy vs. radiotherapy alone in the treatment of primary, nonresectable adenocarcinoma of the rectum. *Int J Radiat Oncol Biol Phys* **2001**, *50*, 427-434, doi:10.1016/s0360-3016(01)01479-1.

68. Holm, T.; Johansson, H.; Rutqvist, L.E.; Cedermark, B. Tumour location and the effects of preoperative radiotherapy in the treatment of rectal cancer. *The British journal of surgery* **2001**, *88*, 839-843, doi:10.1046/j.0007-1323.2001.01789.x.

69. Kapiteijn, E.; Marijnen, C.A.; Nagtegaal, I.D.; Putter, H.; Steup, W.H.; Wiggers, T.; Rutten, H.J.; Pahlman, L.; Glimelius, B.; van Krieken, J.H.; et al. Preoperative radiotherapy combined with total mesorectal excision for resectable rectal cancer. *N Engl J Med* **2001**, *345*, 638-646, doi:10.1056/NEJMoa010580.

70. Kim, N.K.; Min, J.S.; Park, J.K.; Yun, S.H.; Sung, J.S.; Jung, H.C.; Roh, J.K. Intravenous 5-fluorouracil versus oral doxifluridine as preoperative concurrent chemoradiation for locally advanced rectal cancer: prospective randomized trials. *Jpn J Clin Oncol* **2001**, *31*, 25-29, doi:10.1093/jjco/hye009.

71. Marijnen, C.A.; Nagtegaal, I.D.; Klein Kranenbarg, E.; Hermans, J.; van de Velde, C.J.; Leer, J.W.; van Krieken, J.H. No downstaging after short-term preoperative radiotherapy in rectal cancer patients. *Journal of clinical oncology : official journal of the American Society of Clinical Oncology* **2001**, *19*, 1976-1984, doi:10.1200/jco.2001.19.7.1976.

72. Martling, A.; Holm, T.; Johansson, H.; Rutqvist, L.E.; Cedermark, B. The Stockholm II trial on preoperative radiotherapy in rectal carcinoma: long-term follow-up of a population-based study. *Cancer* **2001**, *92*, 896-902, doi:10.1002/1097-0142(20010815)92:4<896::aid-cncr1398>3.0.co;2-r.

73. Tepper, J.E.; O'Connell, M.J.; Niedzwiecki, D.; Hollis, D.; Compton, C.; Benson, A.B., 3rd; Cummings, B.; Gunderson, L.; Macdonald, J.S.; Mayer, R.J. Impact of number of nodes retrieved on outcome in patients with rectal cancer. *Journal of clinical oncology : official journal of the American Society of Clinical Oncology* **2001**, *19*, 157-163, doi:10.1200/jco.2001.19.1.157.

74. Nagawa, H.; Muto, T.; Sunouchi, K.; Higuchi, Y.; Tsurita, G.; Watanabe, T.; Sawada, T. Randomized, controlled trial of lateral node dissection vs. nerve-preserving resection in patients with rectal cancer after preoperative radiotherapy. *Dis Colon Rectum* **2001**, *44*, 1274-1280, doi:10.1007/bf02234784.

75. Lee, J.H.; Lee, J.H.; Ahn, J.H.; Bahng, H.; Kim, T.W.; Kang, Y.K.; Lee, K.H.; Kim, J.C.; Yu, C.S.; Kim, J.H.; et al. Randomized trial of postoperative adjuvant therapy in stage II and III rectal cancer to define the optimal sequence of chemotherapy and radiotherapy: a preliminary report. *Journal of clinical oncology : official journal of the American Society of Clinical Oncology* **2002**, *20*, 1751-1758, doi:10.1200/jco.2002.07.037.

76. Nagtegaal, I.D.; Marijnen, C.A.; Kranenbarg, E.K.; Mulder-Stapel, A.; Hermans, J.; van de Velde, C.J.; van Krieken, J.H. Short-term preoperative radiotherapy interferes with the determination of pathological parameters in rectal cancer. *J Pathol* **2002**, *197*, 20-27, doi:10.1002/path.1098.

77. Nagtegaal, I.D.; van de Velde, C.J.; van der Worp, E.; Kapiteijn, E.; Quirke, P.; van Krieken, J.H. Macroscopic evaluation of rectal cancer resection specimen: clinical significance of the pathologist in quality control. *Journal of clinical oncology : official journal of the American Society of Clinical Oncology* **2002**, *20*, 1729-1734, doi:10.1200/jco.2002.07.010.

78. Nagtegaal, I.D.; Marijnen, C.A.; Kranenbarg, E.K.; van de Velde, C.J.; van Krieken, J.H. Circumferential margin involvement is still an important predictor of local recurrence in rectal carcinoma: not one millimeter but two millimeters is the limit. *Am J Surg Pathol* **2002**, *26*, 350-357, doi:10.1097/00000478-200203000-00009.

79. Sauer, R.; Fietkau, R.; Wittekind, C.; Rödel, C.; Martus, P.; Hohenberger, W.; Tschmelitsch, J.; Sabitzer, H.; Karstens, J.H.; Becker, H.; et al. Adjuvant vs. neoadjuvant radiochemotherapy for locally advanced rectal cancer: the German trial CAO/ARO/AIO-94. *Colorectal disease : the official journal of the Association of Coloproctology of Great Britain and Ireland* **2003**, *5*, 406-415, doi:10.1046/j.1463-1318.2003.00509.x.

80. Araujo, S.E.; da Silva eSousa, A.H., Jr.; de Campos, F.G.; Habr-Gama, A.; Dumarco, R.B.; Caravatto, P.P.; Nahas, S.C.; da Silva, J.; Kiss, D.R.; Gama-Rodrigues, J.J. Conventional approach x laparoscopic abdominoperineal resection for rectal cancer treatment after neoadjuvant chemoradiation: results of a prospective randomized trial. *Rev Hosp Clin Fac Med Sao Paulo* **2003**, *58*, 133-140, doi:10.1590/s0041-87812003000300002.

81. Gennatas, C.; Dardoufas, C.; Mouratidou, D.; Tsavaris, N.; Pouli, A.; Androulakis, G.; Philippakis, M.; Voros, D.; Batalis, T.; Besbeas, S.; et al. Surgical adjuvant therapy of rectal carcinoma: a controlled evaluation of leucovorin, 5-fluorouracil and radiation therapy with or without interferon-alpha2b. *Ann Oncol* **2003**, *14*, 378-382, doi:10.1093/annonc/mdg105.

82. Marijnen, C.A.; Nagtegaal, I.D.; Kapiteijn, E.; Kranenbarg, E.K.; Noordijk, E.M.; van Krieken, J.H.; van de Velde, C.J.; Leer, J.W. Radiotherapy does not compensate for positive resection margins in rectal cancer patients: report of a multicenter randomized trial. *Int J Radiat Oncol Biol Phys* **2003**, *55*, 1311-1320, doi:10.1016/s0360-3016(02)04291-8.

83. Cafiero, F.; Gipponi, M.; Lionetto, R. Randomised clinical trial of adjuvant postoperative RT vs. sequential postoperative RT plus 5-FU and levamisole in patients with stage II-III resectable rectal cancer: a final report. *J Surg Oncol* **2003**, *83*, 140-146, doi:10.1002/jso.10261.

84. James, R.D.; Donaldson, D.; Gray, R.; Northover, J.M.; Stenning, S.P.; Taylor, I. Randomized clinical trial of adjuvant radiotherapy and 5-fluorouracil infusion in colorectal cancer (AXIS). *The British journal of surgery* **2003**, *90*, 1200-1212, doi:10.1002/bjs.4266.

85. Glehen, O.; Chapet, O.; Adham, M.; Nemoz, J.C.; Gerard, J.P. Long-term results of the Lyons R90-01 randomized trial of preoperative radiotherapy with delayed surgery and its effect on sphincter-saving surgery in rectal cancer. *The British journal of surgery* **2003**, *90*, 996-998, doi:10.1002/bjs.4162.

86. Tsavaris, N.; Gennatas, K.; Kosmas, C.; Skopelitis, H.M.; Gouveris, P.; Dimitrakopoulos, A.; Zacharakis, M.; Kouraklis, G.; Vasiliou, J.; Felekouras, E.; et al. Leucovorin and fluorouracil vs levamisole and fluorouracil as adjuvant chemotherapy in rectal cancer. *Oncol Rep* **2004**, *12*, 927-932, doi:10.3892/or.12.4.927.

87. Bosset, J.F.; Calais, G.; Daban, A.; Berger, C.; Radosevic-Jelic, L.; Maingon, P.; Bardet, E.; Pierart, M.; Briffaux, A. Preoperative chemoradiotherapy versus preoperative radiotherapy in rectal cancer patients: assessment of acute toxicity and treatment compliance. Report of the 22921 randomised trial conducted by the EORTC Radiotherapy Group. *Eur J Cancer* **2004**, *40*, 219-224, doi:10.1016/j.ejca.2003.09.032.

88. Habr-Gama, A.; Perez, R.O.; Kiss, D.R.; Rawet, V.; Scanavini, A.; Santinho, P.M.; Nadalin, W. Preoperative chemoradiation therapy for low rectal cancer. Impact on downstaging and sphincter-saving operations. *Hepatogastroenterology* **2004**, *51*, 1703-1707.

89. Sauer, R.; Becker, H.; Hohenberger, W.; Rödel, C.; Wittekind, C.; Fietkau, R.; Martus, P.; Tschmelitsch, J.; Hager, E.; Hess, C.F.; et al. Preoperative versus postoperative chemoradiotherapy for rectal cancer. *N Engl J Med* **2004**, *351*, 1731-1740, doi:10.1056/NEJMoa040694.

90. van den Brink, M.; Stiggelbout, A.M.; van den Hout, W.B.; Kievit, J.; Klein Kranenbarg, E.; Marijnen, C.A.; Nagtegaal, I.D.; Rutten, H.J.; Wiggers, T.; van de Velde, C.J. Clinical nature and prognosis of locally recurrent rectal cancer after total mesorectal excision with or without preoperative radiotherapy. *Journal of clinical oncology : official journal of the American Society of Clinical Oncology* **2004**, *22*, 3958-3964, doi:10.1200/jco.2004.01.023.

91. Watanabe, M.; Nishida, O.; Kunii, Y.; Kodaira, S.; Takahashi, T.; Tominaga, T.; Hojyo, K.; Kato, T.; Niimoto, M.; Kunitomo, K.; et al. Randomized controlled trial of the efficacy of adjuvant immunochemotherapy and adjuvant chemotherapy for colorectal cancer, using different combinations of the intracutaneous streptococcal preparation OK-432 and the oral pyrimidines 1-hexylcarbamoyl-5-fluorouracil and uracil/tegafur. *Int J Clin Oncol* **2004**, *9*, 98-106, doi:10.1007/s10147-003-0374-0.

92. Gerard, J.P.; Chapet, O.; Nemoz, C.; Hartweig, J.; Romestaing, P.; Coquard, R.; Barbet, N.; Maingon, P.; Mahe, M.; Baulieux, J.; et al. Improved sphincter preservation in low rectal cancer with high-dose preoperative radiotherapy: the lyon R96-02 randomized trial. *Journal of clinical oncology : official journal of the American Society of Clinical Oncology* **2004**, *22*, 2404-2409, doi:10.1200/jco.2004.08.170.

93. Bosset, J.F.; Calais, G.; Mineur, L.; Maingon, P.; Radosevic-Jelic, L.; Daban, A.; Bardet, E.; Beny, A.; Briffaux, A.; Collette, L. Enhanced tumorocidal effect of chemotherapy with preoperative radiotherapy for rectal cancer: preliminary results--EORTC 22921. *Journal of clinical oncology : official journal of the American Society of Clinical Oncology* **2005**, *23*, 5620-5627, doi:10.1200/jco.2005.02.113.

94. Folkesson, J.; Birgisson, H.; Pahlman, L.; Cedermark, B.; Glimelius, B.; Gunnarsson, U. Swedish Rectal Cancer Trial: long lasting benefits from radiotherapy on survival and local recurrence rate. *Journal of clinical oncology : official journal of the American Society of Clinical Oncology* **2005**, *23*, 5644-5650, doi:10.1200/jco.2005.08.144.

95. Lezoche, E.; Guerrieri, M.; Paganini, A.M.; D'Ambrosio, G.; Baldarelli, M.; Lezoche, G.; Feliciotti, F.; De Sanctis, A. Transanal endoscopic versus total mesorectal laparoscopic resections of T2-N0 low rectal cancers after neoadjuvant treatment: a prospective randomized trial with a 3-years minimum follow-up period. *Surg Endosc* **2005**, *19*, 751-756, doi:10.1007/s00464-004-8930-x.

96. Nagtegaal, I.D.; van de Velde, C.J.; Marijnen, C.A.; van Krieken, J.H.; Quirke, P. Low rectal cancer: a call for a change of approach in abdominoperineal resection. *Journal of clinical oncology : official journal of the American Society of Clinical Oncology* **2005**, *23*, 9257-9264, doi:10.1200/jco.2005.02.9231.

97. Rödel, C.; Martus, P.; Papadoupolos, T.; Füzesi, L.; Klimpfinger, M.; Fietkau, R.; Liersch, T.; Hohenberger, W.; Raab, R.; Sauer, R.; et al. Prognostic significance of tumor regression after preoperative chemoradiotherapy for rectal cancer. *Journal of clinical oncology : official journal of the American Society of Clinical Oncology* **2005**, *23*, 8688-8696, doi:10.1200/jco.2005.02.1329.

98. Lygidakis, N.J.; Safioleas, M. Multimodality approach towards advanced rectal carcinoma. "Its efficacy and safety". *Hepatogastroenterology* **2005**, *52*, 385-390.

99. Bujko, K.; Nowacki, M.P.; Nasierowska-Guttmejer, A.; Kepka, L.; Winkler-Spytkowska, B.; Suwiński, R.; Oledzki, J.; Stryczyńska, G.; Wieczorek, A.; Serkies, K.; et al. Prediction of mesorectal nodal metastases after chemoradiation for rectal cancer: results of a randomised trial: implication for subsequent local excision. *Radiother Oncol* **2005**, *76*, 234-240, doi:10.1016/j.radonc.2005.04.004.

100. Smalley, S.R.; Benedetti, J.K.; Williamson, S.K.; Robertson, J.M.; Estes, N.C.; Maher, T.; Fisher, B.; Rich, T.A.; Martenson, J.A.; Kugler, J.W.; et al. Phase III trial of fluorouracil-based chemotherapy regimens plus radiotherapy in postoperative adjuvant rectal cancer: GI INT 0144. *Journal of clinical oncology : official journal of the American Society of Clinical Oncology* **2006**, *24*, 3542-3547, doi:10.1200/jco.2005.04.9544.

101. Taher, A.N.; El-Baradie, M.M.; Nasr, A.M.; Khorshid, O.; Morsi, A.; Hamza, M.R.; Mokhtar, N.; Ezzat, S. Locally advanced rectal carcinoma: preoperative radiotherapy versus postoperative chemoradiation, 10-year follow-up results of a randomized clinical study. *J Egypt Natl Canc Inst* **2006**, *18*, 233-243.

102. Akasu, T.; Moriya, Y.; Ohashi, Y.; Yoshida, S.; Shirao, K.; Kodaira, S. Adjuvant chemotherapy with uracil-tegafur for pathological stage III rectal cancer after mesorectal excision with selective lateral pelvic lymphadenectomy: a multicenter randomized controlled trial. *Jpn J Clin Oncol* **2006**, *36*, 237-244, doi:10.1093/jjco/hyl014.

103. Brivio, F.; Fumagalli, L.; Lissoni, P.; Nardone, A.; Nespoli, L.; Fattori, L.; Denova, M.; Chiarelli, M.; Nespoli, A. Pre-operative immunoprophylaxis with interleukin-2 may improve prognosis in radical surgery for colorectal cancer stage B-C. *Anticancer Res* **2006**, *26*, 599-603.

104. de Bruin, E.C.; van de Velde, C.J.; van de Pas, S.; Nagtegaal, I.D.; van Krieken, J.H.; Gosens, M.J.; Peltenburg, L.T.; Medema, J.P.; Marijnen, C.A. Prognostic value of apoptosis in rectal cancer patients of the dutch total mesorectal excision trial: radiotherapy is redundant in intrinsically high-apoptotic tumors. *Clin Cancer Res* **2006**, *12*, 6432-6436, doi:10.1158/1078-0432.Ccr-06-0231.

105. Hildebrandt, B.; Rau, B.; Löffel, J.; Wust, P.; Nicolaou, A.; Gellermann, J.; Le Coutre, P.; Neuhaus, P.; Felix, R.; Wernecke, K.D.; et al. Adjuvant chemotherapy with folinic acid and 5-fluorouracil in patients with locally advanced rectal cancer previously treated by preoperative radiochemotherapy and curative tumor resection. *International journal of colorectal disease* **2006**, *21*, 582-589, doi:10.1007/s00384-005-0054-9.

106. Mohiuddin, M.; Winter, K.; Mitchell, E.; Hanna, N.; Yuen, A.; Nichols, C.; Shane, R.; Hayostek, C.; Willett, C. Randomized phase II study of neoadjuvant combined-modality chemoradiation for distal rectal cancer: Radiation Therapy Oncology Group Trial 0012. *Journal of clinical oncology : official journal of the American Society of Clinical Oncology* **2006**, *24*, 650-655, doi:10.1200/jco.2005.03.6095.

107. Pollack, J.; Holm, T.; Cedermark, B.; Altman, D.; Holmström, B.; Glimelius, B.; Mellgren, A. Late adverse effects of short-course preoperative radiotherapy in rectal cancer. *The British journal of surgery* **2006**, *93*, 1519-1525, doi:10.1002/bjs.5525.

108. Bosset, J.F.; Collette, L.; Calais, G.; Mineur, L.; Maingon, P.; Radosevic-Jelic, L.; Daban, A.; Bardet, E.; Beny, A.; Ollier, J.C. Chemotherapy with preoperative radiotherapy in rectal cancer. *N Engl J Med* **2006**, *355*, 1114-1123, doi:10.1056/NEJMoa060829.

109. Bujko, K.; Nowacki, M.P.; Nasierowska-Guttmejer, A.; Michalski, W.; Bebenek, M.; Kryj, M. Long-term results of a randomized trial comparing preoperative short-course radiotherapy with preoperative conventionally fractionated chemoradiation for rectal cancer. *The British journal of surgery* **2006**, *93*, 1215-1223, doi:10.1002/bjs.5506.

110. Fietkau, R.; Rödel, C.; Hohenberger, W.; Raab, R.; Hess, C.; Liersch, T.; Becker, H.; Wittekind, C.; Hutter, M.; Hager, E.; et al. Rectal cancer delivery of radiotherapy in adequate time and with adequate dose is influenced by treatment center, treatment schedule, and gender and is prognostic parameter for local control: results of study CAO/ARO/AIO-94. *Int J Radiat Oncol Biol Phys* **2007**, *67*, 1008-1019, doi:10.1016/j.ijrobp.2006.10.020.

111. Collette, L.; Bosset, J.F.; den Dulk, M.; Nguyen, F.; Mineur, L.; Maingon, P.; Radosevic-Jelic, L.; Piérart, M.; Calais, G. Patients with curative resection of cT3-4 rectal cancer after preoperative radiotherapy or radiochemotherapy: does anybody benefit from adjuvant fluorouracil-based chemotherapy? A trial of the European Organisation for Research and Treatment of Cancer Radiation Oncology Group. *Journal of clinical oncology : official journal of the American Society of Clinical Oncology* **2007**, *25*, 4379-4386, doi:10.1200/jco.2007.11.9685.

112. Rhomberg, W.; Hammer, J.; Sedlmayer, F.; Eiter, H.; Seewald, D.; Schneider, B. Irradiation with and without razoxane in the treatment of incompletely resected or inoperable recurrent rectal cancer. Results of a small randomized multicenter study. *Strahlenther Onkol* **2007**, *183*, 380-384, doi:10.1007/s00066-007-1617-1.

113. Bujko, K.; Michalski, W.; Kepka, L.; Nowacki, M.P.; Nasierowska-Guttmejer, A.; Tokar, P.; Dymecki, D.; Pawlak, M.; Lesniak, T.; Richter, P.; et al. Association between pathologic response in metastatic lymph nodes after preoperative chemoradiotherapy and risk of distant metastases in rectal cancer: An analysis of outcomes in a randomized trial. *Int J Radiat Oncol Biol Phys* **2007**, *67*, 369-377, doi:10.1016/j.ijrobp.2006.08.065.

114. de Heer, P.; Gosens, M.J.; de Bruin, E.C.; Dekker-Ensink, N.G.; Putter, H.; Marijnen, C.A.; van den Brule, A.J.; van Krieken, J.H.; Rutten, H.J.; Kuppen, P.J.; et al. Cyclooxygenase 2 expression in rectal cancer is of prognostic significance in patients receiving preoperative radiotherapy. *Clin Cancer Res* **2007**, *13*, 2955-2960, doi:10.1158/1078-0432.Ccr-06-2042.

115. den Dulk, M.; Marijnen, C.A.; Putter, H.; Rutten, H.J.; Beets, G.L.; Wiggers, T.; Nagtegaal, I.D.; van de Velde, C.J. Risk factors for adverse outcome in patients with rectal cancer treated with an abdominoperineal resection in the total mesorectal excision trial. *Annals of surgery* **2007**, *246*, 83-90, doi:10.1097/01.sla.0000259432.29056.9d.

116. den Dulk, M.; Collette, L.; van de Velde, C.J.; Marijnen, C.A.; Calais, G.; Mineur, L.; Maingon, P.; Radosevic-Jelic, L.; Daban, A.; Bosset, J.F. Quality of surgery in T3-4 rectal cancer: involvement of circumferential resection margin not influenced by preoperative treatment. Results from EORTC trial 22921. *Eur J Cancer* **2007**, *43*, 1821-1828, doi:10.1016/j.ejca.2007.05.025.

117. Lange, M.M.; den Dulk, M.; Bossema, E.R.; Maas, C.P.; Peeters, K.C.; Rutten, H.J.; Klein Kranenbarg, E.; Marijnen, C.A.; van de Velde, C.J. Risk factors for faecal incontinence after rectal cancer treatment. *The British journal of surgery* **2007**, *94*, 1278-1284, doi:10.1002/bjs.5819.

118. Matthiessen, P.; Hallböök, O.; Rutegård, J.; Simert, G.; Sjödahl, R. Defunctioning stoma reduces symptomatic anastomotic leakage after low anterior resection of the rectum for cancer: a randomized multicenter trial. *Annals of surgery* **2007**, *246*, 207-214, doi:10.1097/SLA.0b013e3180603024.

119. Peeters, K.C.; Marijnen, C.A.; Nagtegaal, I.D.; Kranenbarg, E.K.; Putter, H.; Wiggers, T.; Rutten, H.; Pahlman, L.; Glimelius, B.; Leer, J.W.; et al. The TME trial after a median follow-up of 6 years: increased local control but no survival benefit in irradiated patients with resectable rectal carcinoma. *Annals of surgery* **2007**, *246*, 693-701, doi:10.1097/01.sla.0000257358.56863.ce.

120. Pietrzak, L.; Bujko, K.; Nowacki, M.P.; Kepka, L.; Oledzki, J.; Rutkowski, A.; Szmeja, J.; Kladny, J.; Dymecki, D.; Wieczorek, A.; et al. Quality of life, anorectal and sexual functions after preoperative radiotherapy for rectal cancer: report of a randomised trial. *Radiother Oncol* **2007**, *84*, 217-225, doi:10.1016/j.radonc.2007.07.007.

121. Rink, A.D.; Haaf, F.; Knupper, N.; Vestweber, K.H. Prospective randomised trial comparing ileocaecal interposition and colon-J-pouch as rectal replacement after total mesorectal excision. *International journal of colorectal disease* **2007**, *22*, 153-160, doi:10.1007/s00384-006-0122-9.

122. Kaçar, S.; Varilsüha, C.; Gürkan, A.; Karaca, C. Pre-operative radiochemotherapy for rectal cancer. A prospective randomized trial comparing pre-operative vs. postoperative radiochemotherapy in rectal cancer patients. *Acta Chir Belg* **2008**, *108*, 518-523, doi:10.1080/00015458.2008.11680278.

123. Penopoulos, V.; Handolias, M.; Avgerinos, A.; Maris, T.; Ilias, T.; Issopoulos, N.; Christianopoulos, G.; Betsis, D.; Vrettou, E.; Kitis, G.; et al. A short course of preoperative radiotherapy improves prognosis of operable rectal carcinoma: a case control study. *Hepatogastroenterology* **2008**, *55*, 1280-1287.

124. Kalofonos, H.P.; Bamias, A.; Koutras, A.; Papakostas, P.; Basdanis, G.; Samantas, E.; Karina, M.; Misailidou, D.; Pisanidis, N.; Pentheroudakis, G.; et al. A randomised phase III trial of adjuvant radio-chemotherapy comparing Irinotecan, 5FU and Leucovorin to 5FU and Leucovorin in patients with rectal cancer: a Hellenic Cooperative Oncology Group Study. *Eur J Cancer* **2008**, *44*, 1693-1700, doi:10.1016/j.ejca.2008.05.025.

125. Masaki, T.; Takayama, M.; Matsuoka, H.; Abe, N.; Ueki, H.; Sugiyama, M.; Tonari, A.; Kusuda, J.; Mizumoto, S.; Atomi, Y. Intraoperative radiotherapy for oncological and function-preserving surgery in patients with advanced lower rectal cancer. *Langenbecks Arch Surg* **2008**, *393*, 173-180, doi:10.1007/s00423-007-0260-8.

126. Birgisson, H.; Påhlman, L.; Gunnarsson, U.; Glimelius, B. Late gastrointestinal disorders after rectal cancer surgery with and without preoperative radiation therapy. *The British journal of surgery* **2008**, *95*, 206-213, doi:10.1002/bjs.5918.

127. Braendengen, M.; Tveit, K.M.; Berglund, A.; Birkemeyer, E.; Frykholm, G.; Påhlman, L.; Wiig, J.N.; Byström, P.; Bujko, K.; Glimelius, B. Randomized phase III study comparing preoperative radiotherapy with chemoradiotherapy in nonresectable rectal cancer. *Journal of clinical oncology : official journal of the American Society of Clinical Oncology* **2008**, *26*, 3687-3694, doi:10.1200/jco.2007.15.3858.

128. de la Torre, A.; García-Berrocal, M.I.; Arias, F.; Mariño, A.; Valcárcel, F.; Magallón, R.; Regueiro, C.A.; Romero, J.; Zapata, I.; de la Fuente, C.; et al. Preoperative chemoradiotherapy for rectal cancer: randomized trial comparing oral uracil and tegafur and oral leucovorin vs. intravenous 5-fluorouracil and leucovorin. *Int J Radiat Oncol Biol Phys* **2008**, *70*, 102-110, doi:10.1016/j.ijrobp.2007.05.068.

129. Debucquoy, A.; Libbrecht, L.; Roobrouck, V.; Goethals, L.; McBride, W.; Haustermans, K. Morphological features and molecular markers in rectal cancer from 95 patients included in the European Organisation for Research and Treatment of Cancer 22921 trial: prognostic value and effects of preoperative radio (chemo) therapy. *Eur J Cancer* **2008**, *44*, 791-797, doi:10.1016/j.ejca.2008.02.023.

130. Horisberger, K.; Hofheinz, R.D.; Palma, P.; Volkert, A.K.; Rothenhoefer, S.; Wenz, F.; Hochhaus, A.; Post, S.; Willeke, F. Tumor response to neoadjuvant chemoradiation in rectal cancer: predictor for surgical morbidity? *International journal of colorectal disease* **2008**, *23*, 257-264, doi:10.1007/s00384-007-0408-6.

131. Lange, M.M.; Maas, C.P.; Marijnen, C.A.; Wiggers, T.; Rutten, H.J.; Kranenbarg, E.K.; van de Velde, C.J. Urinary dysfunction after rectal cancer treatment is mainly caused by surgery. *The British journal of surgery* **2008**, *95*, 1020-1028, doi:10.1002/bjs.6126.

132. Lezoche, G.; Baldarelli, M.; Guerrieri, M.; Paganini, A.M.; De Sanctis, A.; Bartolacci, S.; Lezoche, E. A prospective randomized study with a 5-year minimum follow-up evaluation of transanal endoscopic microsurgery versus laparoscopic total mesorectal excision after neoadjuvant therapy. *Surg Endosc* **2008**, *22*, 352-358, doi:10.1007/s00464-007-9596-y.

133. Ulrich, A.B.; Seiler, C.M.; Z'Graggen, K.; Löffler, T.; Weitz, J.; Büchler, M.W. Early results from a randomized clinical trial of colon J pouch versus transverse coloplasty pouch after low anterior resection for rectal cancer. *The British journal of surgery* **2008**, *95*, 1257-1263, doi:10.1002/bjs.6301.

134. Valentini, V.; Coco, C.; Minsky, B.D.; Gambacorta, M.A.; Cosimelli, M.; Bellavita, R.; Morganti, A.G.; La Torre, G.; Trodella, L.; Genovesi, D.; et al. Randomized, multicenter, phase IIb study of preoperative chemoradiotherapy in T3 mid-distal rectal cancer: raltitrexed + oxaliplatin + radiotherapy versus cisplatin + 5-fluorouracil + radiotherapy. *Int J Radiat Oncol Biol Phys* **2008**, *70*, 403-412, doi:10.1016/j.ijrobp.2007.06.025.

135. Rutkowski, A.; Bujko, K.; Nowacki, M.P.; Chmielik, E.; Nasierowska-Guttmejer, A.; Wojnar, A. Distal bowel surgical margin shorter than 1 cm after preoperative radiation for rectal cancer: is it safe? *Annals of surgical oncology* **2008**, *15*, 3124-3131, doi:10.1245/s10434-008-0125-6.

136. Kaçar, S.; Varilsüha, C.; Gürkan, A.; Karaca, C. Pre-operative radiochemotherapy for rectal cancer. A prospective randomized trial comparing pre-operative vs. postoperative radiochemotherapy in rectal cancer patients. *Acta Chir Belg* **2009**, *109*, 701-707, doi:10.1080/00015458.2009.11680520.

137. Roh, M.S.; Colangelo, L.H.; O'Connell, M.J.; Yothers, G.; Deutsch, M.; Allegra, C.J.; Kahlenberg, M.S.; Baez-Diaz, L.; Ursiny, C.S.; Petrelli, N.J.; et al. Preoperative multimodality therapy improves disease-free survival in patients with carcinoma of the rectum: NSABP R-03. *Journal of clinical oncology : official journal of the American Society of Clinical Oncology* **2009**, *27*, 5124-5130, doi:10.1200/jco.2009.22.0467.

138. Buunen, M.; Bonjer, H.J.; Hop, W.C.; Haglind, E.; Kurlberg, G.; Rosenberg, J.; Lacy, A.M.; Cuesta, M.A.; D'Hoore, A.; Fürst, A.; et al. COLOR II. A randomized clinical trial comparing laparoscopic and open surgery for rectal cancer. *Dan Med Bull* **2009**, *56*, 89-91.

139. Debucquoy, A.; Roels, S.; Goethals, L.; Libbrecht, L.; Van Cutsem, E.; Geboes, K.; Penninckx, F.; D'Hoore, A.; McBride, W.H.; Haustermans, K. Double blind randomized phase II study with radiation+5-fluorouracil+/-celecoxib for resectable rectal cancer. *Radiother Oncol* **2009**, *93*, 273-278, doi:10.1016/j.radonc.2009.08.006.

140. Fokstuen, T.; Holm, T.; Glimelius, B. Postoperative morbidity and mortality in relation to leukocyte counts and time to surgery after short-course preoperative radiotherapy for rectal cancer. *Radiother Oncol* **2009**, *93*, 293-297, doi:10.1016/j.radonc.2009.08.034.

141. Keilholz, L.; Mese, M.; Henneking, K.; Willner, J. Effect of total mesorectal excision on the outcome of rectal cancer after standardized postoperative radiochemotherapy: do randomized studies translate into clinical routine? *Strahlenther Onkol* **2009**, *185*, 364-370, doi:10.1007/s00066-009-1940-9.

142. Lange, M.M.; Marijnen, C.A.; Maas, C.P.; Putter, H.; Rutten, H.J.; Stiggelbout, A.M.; Meershoek-Klein Kranenbarg, E.; van de Velde, C.J. Risk factors for sexual dysfunction after rectal cancer treatment. *Eur J Cancer* **2009**, *45*, 1578-1588, doi:10.1016/j.ejca.2008.12.014.

143. Lööf, J.; Pfeifer, D.; Adell, G.; Sun, X.F. Significance of an exon 2 G4C14-to-A4T14 polymorphism in the p73 gene on survival in rectal cancer patients with or without preoperative radiotherapy. *Radiother Oncol* **2009**, *92*, 215-220, doi:10.1016/j.radonc.2009.06.007.

144. Lujan, J.; Valero, G.; Hernandez, Q.; Sanchez, A.; Frutos, M.D.; Parrilla, P. Randomized clinical trial comparing laparoscopic and open surgery in patients with rectal cancer. *The British journal of surgery* **2009**, *96*, 982-989, doi:10.1002/bjs.6662.

145. Matsuoka, H.; Masaki, T.; Takayama, M.; Tonari, A.; Sato, K.; Kobayashi, T.; Sugiyama, M.; Atomi, Y. Impact of intra-operative radiotherapy on evacuatory function following ultra-low anterior resection. A preliminary result of prospective randomized trial. *Hepatogastroenterology* **2009**, *56*, 1656-1660.

146. Parc, Y.; Zutshi, M.; Zalinski, S.; Ruppert, R.; Fürst, A.; Fazio, V.W. Preoperative radiotherapy is associated with worse functional results after coloanal anastomosis for rectal cancer. *Dis Colon Rectum* **2009**, *52*, 2004-2014, doi:10.1007/DCR.0b013e3181beb4d8.

147. Sebag-Montefiore, D.; Stephens, R.J.; Steele, R.; Monson, J.; Grieve, R.; Khanna, S.; Quirke, P.; Couture, J.; de Metz, C.; Myint, A.S.; et al. Preoperative radiotherapy versus selective postoperative chemoradiotherapy in patients with rectal cancer (MRC CR07 and NCIC-CTG C016): a multicentre, randomised trial. *Lancet* **2009**, *373*, 811-820, doi:10.1016/s0140-6736(09)60484-0.

148. Kornmann, M.; Staib, L.; Wiegel, T.; Kreuser, E.D.; Kron, M.; Baumann, W.; Henne-Bruns, D.; Link, K.H. Adjuvant chemoradiotherapy of advanced resectable rectal cancer: results of a randomised trial comparing modulation of 5-fluorouracil with folinic acid or with interferon-α. *British journal of cancer* **2010**, *103*, 1163-1172, doi:10.1038/sj.bjc.6605871.

149. Bujko, K.; Kolodziejczyk, M.; Nasierowska-Guttmejer, A.; Michalski, W.; Kepka, L.; Chmielik, E.; Wojnar, A.; Chwalinski, M. Tumour regression grading in patients with residual rectal cancer after preoperative chemoradiation. *Radiother Oncol* **2010**, *95*, 298-302, doi:10.1016/j.radonc.2010.04.005.

150. Fernández-Martos, C.; Pericay, C.; Aparicio, J.; Salud, A.; Safont, M.; Massuti, B.; Vera, R.; Escudero, P.; Maurel, J.; Marcuello, E.; et al. Phase II, randomized study of concomitant chemoradiotherapy followed by surgery and adjuvant capecitabine plus oxaliplatin (CAPOX) compared with induction CAPOX followed by concomitant chemoradiotherapy and surgery in magnetic resonance imaging-defined, locally advanced rectal cancer: Grupo cancer de recto 3 study. *Journal of clinical oncology : official journal of the American Society of Clinical Oncology* **2010**, *28*, 859-865, doi:10.1200/jco.2009.25.8541.

151. Gérard, J.P.; Azria, D.; Gourgou-Bourgade, S.; Martel-Laffay, I.; Hennequin, C.; Etienne, P.L.; Vendrely, V.; François, E.; de La Roche, G.; Bouché, O.; et al. Comparison of two neoadjuvant chemoradiotherapy regimens for locally advanced rectal cancer: results of the phase III trial ACCORD 12/0405-Prodige 2. *Journal of clinical oncology : official journal of the American Society of Clinical Oncology* **2010**, *28*, 1638-1644, doi:10.1200/jco.2009.25.8376.

152. Kusters, M.; Marijnen, C.A.; van de Velde, C.J.; Rutten, H.J.; Lahaye, M.J.; Kim, J.H.; Beets-Tan, R.G.; Beets, G.L. Patterns of local recurrence in rectal cancer; a study of the Dutch TME trial. *European journal of surgical oncology : the journal of the European Society of Surgical Oncology and the British Association of Surgical Oncology* **2010**, *36*, 470-476, doi:10.1016/j.ejso.2009.11.011.

153. Masaki, T.; Matsuoka, H.; Kobayashi, T.; Abe, N.; Takayama, M.; Tonari, A.; Sugiyama, M.; Atomi, Y. Quality assurance of pelvic autonomic nerve-preserving surgery for advanced lower rectal cancer--preliminary results of a randomized controlled trial. *Langenbecks Arch Surg* **2010**, *395*, 607-613, doi:10.1007/s00423-010-0655-9.

154. Pettersson, D.; Cedermark, B.; Holm, T.; Radu, C.; Påhlman, L.; Glimelius, B.; Martling, A. Interim analysis of the Stockholm III trial of preoperative radiotherapy regimens for rectal cancer. *The British journal of surgery* **2010**, *97*, 580-587, doi:10.1002/bjs.6914.

155. Stephens, R.J.; Thompson, L.C.; Quirke, P.; Steele, R.; Grieve, R.; Couture, J.; Griffiths, G.O.; Sebag-Montefiore, D. Impact of short-course preoperative radiotherapy for rectal cancer on patients' quality of life: data from the Medical Research Council CR07/National Cancer Institute of Canada Clinical Trials Group C016 randomized clinical trial. *Journal of clinical oncology : official journal of the American Society of Clinical Oncology* **2010**, *28*, 4233-4239, doi:10.1200/jco.2009.26.5264.

156. Tiv, M.; Puyraveau, M.; Mineur, L.; Calais, G.; Maingon, P.; Bardet, E.; Mercier, M.; Bosset, J.F. Long-term quality of life in patients with rectal cancer treated with preoperative (chemo)-radiotherapy within a randomized trial. *Cancer Radiother* **2010**, *14*, 530-534, doi:10.1016/j.canrad.2010.06.017.

157. Tunio, M.A.; Rafi, M.; Hashmi, A.; Mohsin, R.; Qayyum, A.; Hasan, M.; Sattar, A.; Mubarak, M. High-dose-rate intraluminal brachytherapy during preoperative chemoradiation for locally advanced rectal cancers. *World J Gastroenterol* **2010**, *16*, 4436-4442, doi:10.3748/wjg.v16.i35.4436.

158. Velenik, V.; Ocvirk, J.; Oblak, I.; Anderluh, F. A phase II study of cetuximab, capecitabine and radiotherapy in neoadjuvant treatment of patients with locally advanced resectable rectal cancer. *European journal of surgical oncology : the journal of the European Society of Surgical Oncology and the British Association of Surgical Oncology* **2010**, *36*, 244-250, doi:10.1016/j.ejso.2009.12.002.

159. Velenik, V.; Oblak, I.; Anderluh, F. Long-term results from a randomized phase II trial of neoadjuvant combined-modality therapy for locally advanced rectal cancer. *Radiat Oncol* **2010**, *5*, 88, doi:10.1186/1748-717x-5-88.

160. Aschele, C.; Cionini, L.; Lonardi, S.; Pinto, C.; Cordio, S.; Rosati, G.; Artale, S.; Tagliagambe, A.; Ambrosini, G.; Rosetti, P.; et al. Primary tumor response to preoperative chemoradiation with or without oxaliplatin in locally advanced rectal cancer: pathologic results of the STAR-01 randomized phase III trial. *Journal of clinical oncology : official journal of the American Society of Clinical Oncology* **2011**, *29*, 2773-2780, doi:10.1200/jco.2010.34.4911.

161. Dubois, J.B.; Bussieres, E.; Richaud, P.; Rouanet, P.; Becouarn, Y.; Mathoulin-Pélissier, S.; Saint-Aubert, B.; Ychou, M. Intra-operative radiotherapy of rectal cancer: results of the French multi-institutional randomized study. *Radiother Oncol* **2011**, *98*, 298-303, doi:10.1016/j.radonc.2011.01.017.

162. Fuller, C.D.; Nijkamp, J.; Duppen, J.C.; Rasch, C.R.; Thomas, C.R., Jr.; Wang, S.J.; Okunieff, P.; Jones, W.E., 3rd; Baseman, D.; Patel, S.; et al. Prospective randomized double-blind pilot study of site-specific consensus atlas implementation for rectal cancer target volume delineation in the cooperative group setting. *Int J Radiat Oncol Biol Phys* **2011**, *79*, 481-489, doi:10.1016/j.ijrobp.2009.11.012.

163. Kim, T.W.; Lee, J.H.; Lee, J.H.; Ahn, J.H.; Kang, Y.K.; Lee, K.H.; Yu, C.S.; Kim, J.H.; Ahn, S.D.; Kim, W.K.; et al. Randomized trial of postoperative adjuvant therapy in Stage II and III rectal cancer to define the optimal sequence of chemotherapy and radiotherapy: 10-year follow-up. *Int J Radiat Oncol Biol Phys* **2011**, *81*, 1025-1031, doi:10.1016/j.ijrobp.2010.07.012.

164. Kim, Y.C.; Lim, J.S.; Keum, K.C.; Kim, K.A.; Myoung, S.; Shin, S.J.; Kim, M.J.; Kim, N.K.; Suh, J.; Kim, K.W. Comparison of diffusion-weighted MRI and MR volumetry in the evaluation of early treatment outcomes after preoperative chemoradiotherapy for locally advanced rectal cancer. *J Magn Reson Imaging* **2011**, *34*, 570-576, doi:10.1002/jmri.22696.

165. Nijkamp, J.; Kusters, M.; Beets-Tan, R.G.; Martijn, H.; Beets, G.L.; van de Velde, C.J.; Marijnen, C.A. Three-dimensional analysis of recurrence patterns in rectal cancer: the cranial border in hypofractionated preoperative radiotherapy can be lowered. *Int J Radiat Oncol Biol Phys* **2011**, *80*, 103-110, doi:10.1016/j.ijrobp.2010.01.046.

166. Park, J.H.; Yoon, S.M.; Yu, C.S.; Kim, J.H.; Kim, T.W.; Kim, J.C. Randomized phase 3 trial comparing preoperative and postoperative chemoradiotherapy with capecitabine for locally advanced rectal cancer. *Cancer* **2011**, *117*, 3703-3712, doi:10.1002/cncr.25943.

167. Rotovnik Kozjek, N.; Kompan, L.; Soeters, P.; Oblak, I.; Mlakar Mastnak, D.; Možina, B.; Zadnik, V.; Anderluh, F.; Velenik, V. Oral glutamine supplementation during preoperative radiochemotherapy in patients with rectal cancer: a randomised double blinded, placebo controlled pilot study. *Clin Nutr* **2011**, *30*, 567-570, doi:10.1016/j.clnu.2011.06.003.

168. Sprenger, T.; Rödel, F.; Beissbarth, T.; Conradi, L.C.; Rothe, H.; Homayounfar, K.; Wolff, H.A.; Ghadimi, B.M.; Yildirim, M.; Becker, H.; et al. Failure of downregulation of survivin following neoadjuvant radiochemotherapy in rectal cancer is associated with distant metastases and shortened survival. *Clin Cancer Res* **2011**, *17*, 1623-1631, doi:10.1158/1078-0432.Ccr-10-2592.

169. van Gijn, W.; Marijnen, C.A.; Nagtegaal, I.D.; Kranenbarg, E.M.; Putter, H.; Wiggers, T.; Rutten, H.J.; Påhlman, L.; Glimelius, B.; van de Velde, C.J. Preoperative radiotherapy combined with total mesorectal excision for resectable rectal cancer: 12-year follow-up of the multicentre, randomised controlled TME trial. *The Lancet. Oncology* **2011**, *12*, 575-582, doi:10.1016/s1470-2045(11)70097-3.

170. Wolff, H.A.; Hennies, S.; Herrmann, M.K.; Rave-Fränk, M.; Eickelmann, D.; Virsik, P.; Jung, K.; Schirmer, M.; Ghadimi, M.; Hess, C.F.; et al. Comparison of the micronucleus and chromosome aberration techniques for the documentation of cytogenetic damage in radiochemotherapy-treated patients with rectal cancer. *Strahlenther Onkol* **2011**, *187*, 52-58, doi:10.1007/s00066-010-2163-9.

171. Brændengen, M.; Tveit, K.M.; Bruheim, K.; Cvancarova, M.; Berglund, Å.; Glimelius, B. Late patient-reported toxicity after preoperative radiotherapy or chemoradiotherapy in nonresectable rectal cancer: results from a randomized Phase III study. *Int J Radiat Oncol Biol Phys* **2011**, *81*, 1017-1024, doi:10.1016/j.ijrobp.2010.07.007.

172. Hofheinz, R.D.; Wenz, F.; Post, S.; Matzdorff, A.; Laechelt, S.; Hartmann, J.T.; Müller, L.; Link, H.; Moehler, M.; Kettner, E.; et al. Chemoradiotherapy with capecitabine versus fluorouracil for locally advanced rectal cancer: a randomised, multicentre, non-inferiority, phase 3 trial. *The Lancet. Oncology* **2012**, *13*, 579-588, doi:10.1016/s1470-2045(12)70116-x.

173. Pach, R.; Kulig, J.; Richter, P.; Gach, T.; Szura, M.; Kowalska, T. Randomized clinical trial on preoperative radiotherapy 25 Gy in rectal cancer--treatment results at 5-year follow-up. *Langenbecks Arch Surg* **2012**, *397*, 801-807, doi:10.1007/s00423-011-0890-8.

174. Salmenkylä, S.; Kouri, M.; Österlund, P.; Pukkala, E.; Luukkonen, P.; Hyöty, M.; Pääkkönen, M.; Mäkelä, J.; Mustonen, H.; Järvinen, H.J. Does preoperative radiotherapy with postoperative chemotherapy increase acute side-effects and postoperative complications of total mesorectal excision? Report of the randomized Finnish rectal cancer trial. *Scand J Surg* **2012**, *101*, 275-282, doi:10.1177/145749691210100410.

175. Sauer, R.; Liersch, T.; Merkel, S.; Fietkau, R.; Hohenberger, W.; Hess, C.; Becker, H.; Raab, H.R.; Villanueva, M.T.; Witzigmann, H.; et al. Preoperative versus postoperative chemoradiotherapy for locally advanced rectal cancer: results of the German CAO/ARO/AIO-94 randomized phase III trial after a median follow-up of 11 years. *Journal of clinical oncology : official journal of the American Society of Clinical Oncology* **2012**, *30*, 1926-1933, doi:10.1200/jco.2011.40.1836.

176. Brændengen, M.; Tveit, K.M.; Hjermstad, M.J.; Johansson, H.; Berglund, Å.; Brandberg, Y.; Glimelius, B. Health-related quality of life (HRQoL) after multimodal treatment for primarily non-resectable rectal cancer. Long-term results from a phase III study. *Eur J Cancer* **2012**, *48*, 813-819, doi:10.1016/j.ejca.2011.06.035.

177. Dewdney, A.; Cunningham, D.; Tabernero, J.; Capdevila, J.; Glimelius, B.; Cervantes, A.; Tait, D.; Brown, G.; Wotherspoon, A.; Gonzalez de Castro, D.; et al. Multicenter randomized phase II clinical trial comparing neoadjuvant oxaliplatin, capecitabine, and preoperative radiotherapy with or without cetuximab followed by total mesorectal excision in patients with high-risk rectal cancer (EXPERT-C). *Journal of clinical oncology : official journal of the American Society of Clinical Oncology* **2012**, *30*, 1620-1627, doi:10.1200/jco.2011.39.6036.

178. Doeksen, A.; Bakx, R.; Vincent, A.; van Tets, W.F.; Sprangers, M.A.; Gerhards, M.F.; Bemelman, W.A.; van Lanschot, J.J. J-pouch vs side-to-end coloanal anastomosis after preoperative radiotherapy and total mesorectal excision for rectal cancer: a multicentre randomized trial. *Colorectal disease : the official journal of the Association of Coloproctology of Great Britain and Ireland* **2012**, *14*, 705-713, doi:10.1111/j.1463-1318.2011.02725.x.

179. Gérard, J.P.; Azria, D.; Gourgou-Bourgade, S.; Martel-Lafay, I.; Hennequin, C.; Etienne, P.L.; Vendrely, V.; François, E.; de La Roche, G.; Bouché, O.; et al. Clinical outcome of the ACCORD 12/0405 PRODIGE 2 randomized trial in rectal cancer. *Journal of clinical oncology : official journal of the American Society of Clinical Oncology* **2012**, *30*, 4558-4565, doi:10.1200/jco.2012.42.8771.

180. Jakobsen, A.; Ploen, J.; Vuong, T.; Appelt, A.; Lindebjerg, J.; Rafaelsen, S.R. Dose-effect relationship in chemoradiotherapy for locally advanced rectal cancer: a randomized trial comparing two radiation doses. *Int J Radiat Oncol Biol Phys* **2012**, *84*, 949-954, doi:10.1016/j.ijrobp.2012.02.006.

181. Latkauskas, T.; Pauzas, H.; Gineikiene, I.; Janciauskiene, R.; Juozaityte, E.; Saladzinskas, Z.; Tamelis, A.; Pavalkis, D. Initial results of a randomized controlled trial comparing clinical and pathological downstaging of rectal cancer after preoperative short-course radiotherapy or long-term chemoradiotherapy, both with delayed surgery. *Colorectal disease : the official journal of the Association of Coloproctology of Great Britain and Ireland* **2012**, *14*, 294-298, doi:10.1111/j.1463-1318.2011.02815.x.

182. Maréchal, R.; Vos, B.; Polus, M.; Delaunoit, T.; Peeters, M.; Demetter, P.; Hendlisz, A.; Demols, A.; Franchimont, D.; Verset, G.; et al. Short course chemotherapy followed by concomitant chemoradiotherapy and surgery in locally advanced rectal cancer: a randomized multicentric phase II study. *Ann Oncol* **2012**, *23*, 1525-1530, doi:10.1093/annonc/mdr473.

183. Ngan, S.Y.; Burmeister, B.; Fisher, R.J.; Solomon, M.; Goldstein, D.; Joseph, D.; Ackland, S.P.; Schache, D.; McClure, B.; McLachlan, S.A.; et al. Randomized trial of short-course radiotherapy versus long-course chemoradiation comparing rates of local recurrence in patients with T3 rectal cancer: Trans-Tasman Radiation Oncology Group trial 01.04. *Journal of clinical oncology : official journal of the American Society of Clinical Oncology* **2012**, *30*, 3827-3833, doi:10.1200/jco.2012.42.9597.

184. Niazi, T.M.; Vuong, T.; Azoulay, L.; Marijnen, C.; Bujko, K.; Nasr, E.; Lambert, C.; Duclos, M.; Faria, S.; David, M.; et al. Silver clear nylon dressing is effective in preventing radiation-induced dermatitis in patients with lower gastrointestinal cancer: results from a phase III study. *Int J Radiat Oncol Biol Phys* **2012**, *84*, e305-310, doi:10.1016/j.ijrobp.2012.03.062.

185. Ortholan, C.; Romestaing, P.; Chapet, O.; Gerard, J.P. Correlation in rectal cancer between clinical tumor response after neoadjuvant radiotherapy and sphincter or organ preservation: 10-year results of the Lyon R 96-02 randomized trial. *Int J Radiat Oncol Biol Phys* **2012**, *83*, e165-171, doi:10.1016/j.ijrobp.2011.12.002.

186. Rödel, C.; Liersch, T.; Becker, H.; Fietkau, R.; Hohenberger, W.; Hothorn, T.; Graeven, U.; Arnold, D.; Lang-Welzenbach, M.; Raab, H.R.; et al. Preoperative chemoradiotherapy and postoperative chemotherapy with fluorouracil and oxaliplatin versus fluorouracil alone in locally advanced rectal cancer: initial results of the German CAO/ARO/AIO-04 randomised phase 3 trial. *The Lancet. Oncology* **2012**, *13*, 679-687, doi:10.1016/s1470-2045(12)70187-0.

187. Wong, S.J.; Winter, K.; Meropol, N.J.; Anne, P.R.; Kachnic, L.; Rashid, A.; Watson, J.C.; Mitchell, E.; Pollock, J.; Lee, R.J.; et al. Radiation Therapy Oncology Group 0247: a randomized Phase II study of neoadjuvant capecitabine and irinotecan or capecitabine and oxaliplatin with concurrent radiotherapy for patients with locally advanced rectal cancer. *Int J Radiat Oncol Biol Phys* **2012**, *82*, 1367-1375, doi:10.1016/j.ijrobp.2011.05.027.

188. van den Broek, C.B.; Vermeer, T.A.; Bastiaannet, E.; Rutten, H.J.; van de Velde, C.J.; Marijnen, C.A. Impact of the interval between short-course radiotherapy and surgery on outcomes of rectal cancer patients. *Eur J Cancer* **2013**, *49*, 3131-3139, doi:10.1016/j.ejca.2013.05.025.

189. Bujko, K.; Nasierowska-Guttmejer, A.; Wyrwicz, L.; Malinowska, M.; Krynski, J.; Kosakowska, E.; Rutkowski, A.; Pietrzak, L.; Kepka, L.; Radziszewski, J.; et al. Neoadjuvant treatment for unresectable rectal cancer: an interim analysis of a multicentre randomized study. *Radiother Oncol* **2013**, *107*, 171-177, doi:10.1016/j.radonc.2013.03.001.

190. Engineer, R.; Mohandas, K.M.; Shukla, P.J.; Shrikhande, S.V.; Mahantshetty, U.; Chopra, S.; Goel, M.; Mehta, S.; Patil, P.; Ramadwar, M.; et al. Escalated radiation dose alone vs. concurrent chemoradiation for locally advanced and unresectable rectal cancers: results from phase II randomized study. *International journal of colorectal disease* **2013**, *28*, 959-966, doi:10.1007/s00384-012-1630-4.

191. Helbling, D.; Bodoky, G.; Gautschi, O.; Sun, H.; Bosman, F.; Gloor, B.; Burkhard, R.; Winterhalder, R.; Madlung, A.; Rauch, D.; et al. Neoadjuvant chemoradiotherapy with or without panitumumab in patients with wild-type KRAS, locally advanced rectal cancer (LARC): a randomized, multicenter, phase II trial SAKK 41/07. *Ann Oncol* **2013**, *24*, 718-725, doi:10.1093/annonc/mds519.

192. Mohammadzadeh, M.; Faramarzi, E.; Mahdavi, R.; Nasirimotlagh, B.; Asghari Jafarabadi, M. Effect of conjugated linoleic acid supplementation on inflammatory factors and matrix metalloproteinase enzymes in rectal cancer patients undergoing chemoradiotherapy. *Integr Cancer Ther* **2013**, *12*, 496-502, doi:10.1177/1534735413485417.

193. Mohiuddin, M.; Paulus, R.; Mitchell, E.; Hanna, N.; Yuen, A.; Nichols, R.; Yalavarthi, S.; Hayostek, C.; Willett, C. Neoadjuvant chemoradiation for distal rectal cancer: 5-year updated results of a randomized phase 2 study of neoadjuvant combined modality chemoradiation for distal rectal cancer. *Int J Radiat Oncol Biol Phys* **2013**, *86*, 523-528, doi:10.1016/j.ijrobp.2013.02.020.

194. Pettersson, D.; Glimelius, B.; Iversen, H.; Johansson, H.; Holm, T.; Martling, A. Impaired postoperative leucocyte counts after preoperative radiotherapy for rectal cancer in the Stockholm III Trial. *The British journal of surgery* **2013**, *100*, 969-975, doi:10.1002/bjs.9117.

195. Rullier, A.; Gourgou-Bourgade, S.; Jarlier, M.; Bibeau, F.; Chassagne-Clément, C.; Hennequin, C.; Tisseau, L.; Leroux, A.; Ettore, F.; Peoc'h, M.; et al. Predictive factors of positive circumferential resection margin after radiochemotherapy for rectal cancer: the French randomised trial ACCORD12/0405 PRODIGE 2. *Eur J Cancer* **2013**, *49*, 82-89, doi:10.1016/j.ejca.2012.06.028.

196. Sclafani, F.; Roy, A.; Cunningham, D.; Wotherspoon, A.; Peckitt, C.; Gonzalez de Castro, D.; Tabernero, J.; Glimelius, B.; Cervantes, A.; Eltahir, Z.; et al. HER2 in high-risk rectal cancer patients treated in EXPERT-C, a randomized phase II trial of neoadjuvant capecitabine and oxaliplatin (CAPOX) and chemoradiotherapy (CRT) with or without cetuximab. *Ann Oncol* **2013**, *24*, 3123-3128, doi:10.1093/annonc/mdt408.

197. van der Pas, M.H.; Haglind, E.; Cuesta, M.A.; Fürst, A.; Lacy, A.M.; Hop, W.C.; Bonjer, H.J. Laparoscopic versus open surgery for rectal cancer (COLOR II): short-term outcomes of a randomised, phase 3 trial. *The Lancet. Oncology* **2013**, *14*, 210-218, doi:10.1016/s1470-2045(13)70016-0.

198. Wolff, H.A.; Conradi, L.C.; Beissbarth, T.; Leha, A.; Hohenberger, W.; Merkel, S.; Fietkau, R.; Raab, H.R.; Tschmelitsch, J.; Hess, C.F.; et al. Gender affects acute organ toxicity during radiochemotherapy for rectal cancer: long-term results of the German CAO/ARO/AIO-94 phase III trial. *Radiother Oncol* **2013**, *108*, 48-54, doi:10.1016/j.radonc.2013.05.009.

199. Kotti, A.; Holmqvist, A.; Albertsson, M.; Sun, X.F. SPARCL1 expression increases with preoperative radiation therapy and predicts better survival in rectal cancer patients. *Int J Radiat Oncol Biol Phys* **2014**, *88*, 1196-1202, doi:10.1016/j.ijrobp.2013.12.041.

200. Bosset, J.F.; Calais, G.; Mineur, L.; Maingon, P.; Stojanovic-Rundic, S.; Bensadoun, R.J.; Bardet, E.; Beny, A.; Ollier, J.C.; Bolla, M.; et al. Fluorouracil-based adjuvant chemotherapy after preoperative chemoradiotherapy in rectal cancer: long-term results of the EORTC 22921 randomised study. *The Lancet. Oncology* **2014**, *15*, 184-190, doi:10.1016/s1470-2045(13)70599-0.

201. Sainato, A.; Cernusco Luna Nunzia, V.; Valentini, V.; De Paoli, A.; Maurizi, E.R.; Lupattelli, M.; Aristei, C.; Vidali, C.; Conti, M.; Galardi, A.; et al. No benefit of adjuvant Fluorouracil Leucovorin chemotherapy after neoadjuvant chemoradiotherapy in locally advanced cancer of the rectum (LARC): Long term results of a randomized trial (I-CNR-RT). *Radiother Oncol* **2014**, *113*, 223-229, doi:10.1016/j.radonc.2014.10.006.

202. Andersson, J.; Abis, G.; Gellerstedt, M.; Angenete, E.; Angerås, U.; Cuesta, M.A.; Jess, P.; Rosenberg, J.; Bonjer, H.J.; Haglind, E. Patient-reported genitourinary dysfunction after laparoscopic and open rectal cancer surgery in a randomized trial (COLOR II). *The British journal of surgery* **2014**, *101*, 1272-1279, doi:10.1002/bjs.9550.

203. Appelt, A.L.; Vogelius, I.R.; Pløen, J.; Rafaelsen, S.R.; Lindebjerg, J.; Havelund, B.M.; Bentzen, S.M.; Jakobsen, A. Long-term results of a randomized trial in locally advanced rectal cancer: no benefit from adding a brachytherapy boost. *Int J Radiat Oncol Biol Phys* **2014**, *90*, 110-118, doi:10.1016/j.ijrobp.2014.05.023.

204. Boelens, P.G.; Heesakkers, F.F.; Luyer, M.D.; van Barneveld, K.W.; de Hingh, I.H.; Nieuwenhuijzen, G.A.; Roos, A.N.; Rutten, H.J. Reduction of postoperative ileus by early enteral nutrition in patients undergoing major rectal surgery: prospective, randomized, controlled trial. *Annals of surgery* **2014**, *259*, 649-655, doi:10.1097/sla.0000000000000288.

205. Borg, C.; André, T.; Mantion, G.; Boudghène, F.; Mornex, F.; Maingon, P.; Adenis, A.; Azria, D.; Piutti, M.; Morsli, O.; et al. Pathological response and safety of two neoadjuvant strategies with bevacizumab in MRI-defined locally advanced T3 resectable rectal cancer: a randomized, noncomparative phase II study. *Ann Oncol* **2014**, *25*, 2205-2210, doi:10.1093/annonc/mdu377.

206. Borowski, D.W.; Banky, B.; Banerjee, A.K.; Agarwal, A.K.; Tabaqchali, M.A.; Garg, D.K.; Hobday, C.; Hegab, M.; Gill, T.S. Intra-arterial methylene blue injection into ex vivo colorectal cancer specimens improves lymph node staging accuracy: a randomized controlled trial. *Colorectal disease : the official journal of the Association of Coloproctology of Great Britain and Ireland* **2014**, *16*, 681-689, doi:10.1111/codi.12681.

207. Fokas, E.; Liersch, T.; Fietkau, R.; Hohenberger, W.; Beissbarth, T.; Hess, C.; Becker, H.; Ghadimi, M.; Mrak, K.; Merkel, S.; et al. Tumor regression grading after preoperative chemoradiotherapy for locally advanced rectal carcinoma revisited: updated results of the CAO/ARO/AIO-94 trial. *Journal of clinical oncology : official journal of the American Society of Clinical Oncology* **2014**, *32*, 1554-1562, doi:10.1200/jco.2013.54.3769.

208. François, E.; Azria, D.; Gourgou-Bourgade, S.; Jarlier, M.; Martel-Laffay, I.; Hennequin, C.; Etienne, P.L.; Vendrely, V.; Seitz, J.F.; Conroy, T.; et al. Results in the elderly with locally advanced rectal cancer from the ACCOR12/PRODIGE 2 phase III trial: tolerance and efficacy. *Radiother Oncol* **2014**, *110*, 144-149, doi:10.1016/j.radonc.2013.10.019.

209. Glynne-Jones, R.; Counsell, N.; Quirke, P.; Mortensen, N.; Maraveyas, A.; Meadows, H.M.; Ledermann, J.; Sebag-Montefiore, D. Chronicle: results of a randomised phase III trial in locally advanced rectal cancer after neoadjuvant chemoradiation randomising postoperative adjuvant capecitabine plus oxaliplatin (XELOX) versus control. *Ann Oncol* **2014**, *25*, 1356-1362, doi:10.1093/annonc/mdu147.

210. Hong, Y.S.; Nam, B.H.; Kim, K.P.; Kim, J.E.; Park, S.J.; Park, Y.S.; Park, J.O.; Kim, S.Y.; Kim, T.Y.; Kim, J.H.; et al. Oxaliplatin, fluorouracil, and leucovorin versus fluorouracil and leucovorin as adjuvant chemotherapy for locally advanced rectal cancer after preoperative chemoradiotherapy (ADORE): an open-label, multicentre, phase 2, randomised controlled trial. *The Lancet. Oncology* **2014**, *15*, 1245-1253, doi:10.1016/s1470-2045(14)70377-8.

211. Jeong, S.Y.; Park, J.W.; Nam, B.H.; Kim, S.; Kang, S.B.; Lim, S.B.; Choi, H.S.; Kim, D.W.; Chang, H.J.; Kim, D.Y.; et al. Open versus laparoscopic surgery for mid-rectal or low-rectal cancer after neoadjuvant chemoradiotherapy (COREAN trial): survival outcomes of an open-label, non-inferiority, randomised controlled trial. *The Lancet. Oncology* **2014**, *15*, 767-774, doi:10.1016/s1470-2045(14)70205-0.

212. Mavroidis, P.; Giantsoudis, D.; Awan, M.J.; Nijkamp, J.; Rasch, C.R.; Duppen, J.C.; Thomas, C.R., Jr.; Okunieff, P.; Jones, W.E., 3rd; Kachnic, L.A.; et al. Consequences of anorectal cancer atlas implementation in the cooperative group setting: radiobiologic analysis of a prospective randomized in silico target delineation study. *Radiother Oncol* **2014**, *112*, 418-424, doi:10.1016/j.radonc.2014.05.011.

213. Ng, S.S.; Lee, J.F.; Yiu, R.Y.; Li, J.C.; Hon, S.S.; Mak, T.W.; Ngo, D.K.; Leung, W.W.; Leung, K.L. Laparoscopic-assisted versus open total mesorectal excision with anal sphincter preservation for mid and low rectal cancer: a prospective, randomized trial. *Surg Endosc* **2014**, *28*, 297-306, doi:10.1007/s00464-013-3187-x.

214. O'Connell, M.J.; Colangelo, L.H.; Beart, R.W.; Petrelli, N.J.; Allegra, C.J.; Sharif, S.; Pitot, H.C.; Shields, A.F.; Landry, J.C.; Ryan, D.P.; et al. Capecitabine and oxaliplatin in the preoperative multimodality treatment of rectal cancer: surgical end points from National Surgical Adjuvant Breast and Bowel Project trial R-04. *Journal of clinical oncology : official journal of the American Society of Clinical Oncology* **2014**, *32*, 1927-1934, doi:10.1200/jco.2013.53.7753.

215. Rutkowski, A.; Zając, L.; Pietrzak, L.; Bednarczyk, M.; Byszek, A.; Oledzki, J.; Olesiński, T.; Szpakowski, M.; Saramak, P.; Chwalinski, M. Surgical site infections following short-term radiotherapy and total mesorectal excision: results of a randomized study examining the role of gentamicin collagen implant in rectal cancer surgery. *Tech Coloproctol* **2014**, *18*, 921-928, doi:10.1007/s10151-014-1193-1.

216. Sclafani, F.; Gonzalez, D.; Cunningham, D.; Hulkki Wilson, S.; Peckitt, C.; Giralt, J.; Glimelius, B.; Roselló Keränen, S.; Wotherspoon, A.; Brown, G.; et al. RAS mutations and cetuximab in locally advanced rectal cancer: results of the EXPERT-C trial. *Eur J Cancer* **2014**, *50*, 1430-1436, doi:10.1016/j.ejca.2014.02.002.

217. Touny, A.; Othman, H.; Maamoon, S.; Ramzy, S.; Elmarakby, H. Perineal reconstruction using pedicled vertical rectus abdominis myocutaneous flap (VRAM). *J Surg Oncol* **2014**, *110*, 752-757, doi:10.1002/jso.23692.

218. Wiltink, L.M.; Chen, T.Y.; Nout, R.A.; Kranenbarg, E.M.; Fiocco, M.; Laurberg, S.; van de Velde, C.J.; Marijnen, C.A. Health-related quality of life 14 years after preoperative short-term radiotherapy and total mesorectal excision for rectal cancer: report of a multicenter randomised trial. *Eur J Cancer* **2014**, *50*, 2390-2398, doi:10.1016/j.ejca.2014.06.020.

219. Fernandez-Martos, C.; Garcia-Albeniz, X.; Pericay, C.; Maurel, J.; Aparicio, J.; Montagut, C.; Safont, M.J.; Salud, A.; Vera, R.; Massuti, B.; et al. Chemoradiation, surgery and adjuvant chemotherapy versus induction chemotherapy followed by chemoradiation and surgery: long-term results of the Spanish GCR-3 phase II randomized trial†. *Ann Oncol* **2015**, *26*, 1722-1728, doi:10.1093/annonc/mdv223.

220. Breugom, A.J.; van Gijn, W.; Muller, E.W.; Berglund, Å.; van den Broek, C.B.M.; Fokstuen, T.; Gelderblom, H.; Kapiteijn, E.; Leer, J.W.H.; Marijnen, C.A.M.; et al. Adjuvant chemotherapy for rectal cancer patients treated with preoperative (chemo)radiotherapy and total mesorectal excision: a Dutch Colorectal Cancer Group (DCCG) randomized phase III trial. *Ann Oncol* **2015**, *26*, 696-701, doi:10.1093/annonc/mdu560.

221. Allegra, C.J.; Yothers, G.; O'Connell, M.J.; Beart, R.W.; Wozniak, T.F.; Pitot, H.C.; Shields, A.F.; Landry, J.C.; Ryan, D.P.; Arora, A.; et al. Neoadjuvant 5-FU or Capecitabine Plus Radiation With or Without Oxaliplatin in Rectal Cancer Patients: A Phase III Randomized Clinical Trial. *Journal of the National Cancer Institute* **2015**, *107*, doi:10.1093/jnci/djv248.

222. Chen, T.Y.; Wiltink, L.M.; Nout, R.A.; Meershoek-Klein Kranenbarg, E.; Laurberg, S.; Marijnen, C.A.; van de Velde, C.J. Bowel function 14 years after preoperative short-course radiotherapy and total mesorectal excision for rectal cancer: report of a multicenter randomized trial. *Clin Colorectal Cancer* **2015**, *14*, 106-114, doi:10.1016/j.clcc.2014.12.007.

223. Delbaldo, C.; Ychou, M.; Zawadi, A.; Douillard, J.Y.; André, T.; Guerin-Meyer, V.; Rougier, P.; Dupuis, O.; Faroux, R.; Jouhaud, A.; et al. Postoperative irinotecan in resected stage II-III rectal cancer: final analysis of the French R98 Intergroup trial†. *Ann Oncol* **2015**, *26*, 1208-1215, doi:10.1093/annonc/mdv135.

224. Fan, W.H.; Wang, F.L.; Lu, Z.H.; Pan, Z.Z.; Li, L.R.; Gao, Y.H.; Chen, G.; Wu, X.J.; Ding, P.R.; Zeng, Z.F.; et al. Surgery with versus without preoperative concurrent chemoradiotherapy for mid/low rectal cancer: an interim analysis of a prospective, randomized trial. *Chin J Cancer* **2015**, *34*, 394-403, doi:10.1186/s40880-015-0024-8.

225. Frøseth, T.C.; Strickert, T.; Solli, K.S.; Salvesen, Ø.; Frykholm, G.; Reidunsdatter, R.J. A randomized study of the effect of patient positioning on setup reproducibility and dose distribution to organs at risk in radiotherapy of rectal cancer patients. *Radiat Oncol* **2015**, *10*, 217, doi:10.1186/s13014-015-0524-3.

226. Gérard, J.P.; Chamorey, E.; Gourgou-Bourgade, S.; Benezery, K.; de Laroche, G.; Mahé, M.A.; Boige, V.; Juzyna, B. Clinical complete response (cCR) after neoadjuvant chemoradiotherapy and conservative treatment in rectal cancer. Findings from the ACCORD 12/PRODIGE 2 randomized trial. *Radiother Oncol* **2015**, *115*, 246-252, doi:10.1016/j.radonc.2015.04.003.

227. Jung, M.; Shin, S.J.; Koom, W.S.; Jung, I.; Keum, K.C.; Hur, H.; Min, B.S.; Baik, S.H.; Kim, N.K.; Kim, H.; et al. A Randomized Phase 2 Study of Neoadjuvant Chemoradiaton Therapy With 5-Fluorouracil/Leucovorin or Irinotecan/S-1 in Patients With Locally Advanced Rectal Cancer. *Int J Radiat Oncol Biol Phys* **2015**, *93*, 1015-1022, doi:10.1016/j.ijrobp.2015.08.037.

228. Pettersson, D.; Lörinc, E.; Holm, T.; Iversen, H.; Cedermark, B.; Glimelius, B.; Martling, A. Tumour regression in the randomized Stockholm III Trial of radiotherapy regimens for rectal cancer. *The British journal of surgery* **2015**, *102*, 972-978; discussion 978, doi:10.1002/bjs.9811.

229. Rödel, C.; Graeven, U.; Fietkau, R.; Hohenberger, W.; Hothorn, T.; Arnold, D.; Hofheinz, R.D.; Ghadimi, M.; Wolff, H.A.; Lang-Welzenbach, M.; et al. Oxaliplatin added to fluorouracil-based preoperative chemoradiotherapy and postoperative chemotherapy of locally advanced rectal cancer (the German CAO/ARO/AIO-04 study): final results of the multicentre, open-label, randomised, phase 3 trial. *The Lancet. Oncology* **2015**, *16*, 979-989, doi:10.1016/s1470-2045(15)00159-x.

230. Salazar, R.; Capdevila, J.; Laquente, B.; Manzano, J.L.; Pericay, C.; Villacampa, M.M.; López, C.; Losa, F.; Safont, M.J.; Gómez, A.; et al. A randomized phase II study of capecitabine-based chemoradiation with or without bevacizumab in resectable locally advanced rectal cancer: clinical and biological features. *BMC Cancer* **2015**, *15*, 60, doi:10.1186/s12885-015-1053-z.

231. Sclafani, F.; Chau, I.; Cunningham, D.; Peckitt, C.; Lampis, A.; Hahne, J.C.; Braconi, C.; Tabernero, J.; Glimelius, B.; Cervantes, A.; et al. Prognostic role of the LCS6 KRAS variant in locally advanced rectal cancer: results of the EXPERT-C trial. *Ann Oncol* **2015**, *26*, 1936-1941, doi:10.1093/annonc/mdv285.

232. Sclafani, F.; Peckitt, C.; Cunningham, D.; Tait, D.; Giralt, J.; Glimelius, B.; Keränen, S.R.; Bateman, A.; Hickish, T.; Tabernero, J.; et al. Short- and Long-Term Quality of Life and Bowel Function in Patients With MRI-Defined, High-Risk, Locally Advanced Rectal Cancer Treated With an Intensified Neoadjuvant Strategy in the Randomized Phase 2 EXPERT-C Trial. *Int J Radiat Oncol Biol Phys* **2015**, *93*, 303-312, doi:10.1016/j.ijrobp.2015.03.038.

233. Vidal-Casariego, A.; Hernando-Martín, M.; Calleja-Fernández, A.; Cano-Rodríguez, I.; Cordido, F.; Ballesteros-Pomar, M.D. Tissue effects of glutamine in rectal cancer patients treated with preoperative chemoradiotherapy. *Nutr Hosp* **2015**, *31*, 1689-1692, doi:10.3305/nh.2015.31.4.8521.

234. Ansari, N.; Young, C.J.; Schlub, T.E.; Dhillon, H.M.; Solomon, M.J. Understanding surgeon decision making in the use of radiotherapy as neoadjuvant treatment in rectal cancer. *Int J Surg* **2015**, *24*, 1-6, doi:10.1016/j.ijsu.2015.10.004.

235. Bujko, K.; Wyrwicz, L.; Rutkowski, A.; Malinowska, M.; Pietrzak, L.; Kryński, J.; Michalski, W.; Olędzki, J.; Kuśnierz, J.; Zając, L.; et al. Long-course oxaliplatin-based preoperative chemoradiation versus 5 × 5 Gy and consolidation chemotherapy for cT4 or fixed cT3 rectal cancer: results of a randomized phase III study. *Ann Oncol* **2016**, *27*, 834-842, doi:10.1093/annonc/mdw062.

236. Cho, H.; Kim, J.E.; Kim, K.P.; Yu, C.S.; Kim, J.C.; Kim, J.H.; Lee, M.A.; Jang, H.S.; Oh, S.T.; Kim, S.Y.; et al. Phase II Study of Preoperative Capecitabine and Oxaliplatin-based Intensified Chemoradiotherapy With or Without Induction Chemotherapy in Patients With Locally Advanced Rectal Cancer and Synchronous Liver-limited Resectable Metastases. *Am J Clin Oncol* **2016**, *39*, 623-629, doi:10.1097/coc.0000000000000315.

237. Deng, Y.; Chi, P.; Lan, P.; Wang, L.; Chen, W.; Cui, L.; Chen, D.; Cao, J.; Wei, H.; Peng, X.; et al. Modified FOLFOX6 With or Without Radiation Versus Fluorouracil and Leucovorin With Radiation in Neoadjuvant Treatment of Locally Advanced Rectal Cancer: Initial Results of the Chinese FOWARC Multicenter, Open-Label, Randomized Three-Arm Phase III Trial. *Journal of clinical oncology : official journal of the American Society of Clinical Oncology* **2016**, *34*, 3300-3307, doi:10.1200/jco.2016.66.6198.

238. Feng, Y.R.; Zhu, Y.; Liu, L.Y.; Wang, W.H.; Wang, S.L.; Song, Y.W.; Wang, X.; Tang, Y.; Liu, Y.P.; Ren, H.; et al. Interim analysis of postoperative chemoradiotherapy with capecitabine and oxaliplatin versus capecitabine alone for pathological stage II and III rectal cancer: a randomized multicenter phase III trial. *Oncotarget* **2016**, *7*, 25576-25584, doi:10.18632/oncotarget.8226.

239. Garrer, W.Y.; El Hossieny, H.A.; Gad, Z.S.; Namour, A.E.; Abo Amer, S.M. Appropriate Timing of Surgery after Neoadjuvant ChemoRadiation Therapy for Locally Advanced Rectal Cancer. *Asian Pac J Cancer Prev* **2016**, *17*, 4381-4389.

240. Huang, M.; Lin, J.; Yu, X.; Chen, S.; Kang, L.; Deng, Y.; Zheng, J.; Luo, Y.; Wang, L.; Lan, P.; et al. Erectile and urinary function in men with rectal cancer treated by neoadjuvant chemoradiotherapy and neoadjuvant chemotherapy alone: a randomized trial report. *International journal of colorectal disease* **2016**, *31*, 1349-1357, doi:10.1007/s00384-016-2605-7.

241. Kye, B.H.; Kim, H.J.; Kim, G.; Yoo, R.N.; Cho, H.M. The Effect of Biofeedback Therapy on Anorectal Function After the Reversal of Temporary Stoma When Administered During the Temporary Stoma Period in Rectal Cancer Patients With Sphincter-Saving Surgery: The Interim Report of a Prospective Randomized Controlled Trial. *Medicine (Baltimore)* **2016**, *95*, e3611, doi:10.1097/md.0000000000003611.

242. Latkauskas, T.; Pauzas, H.; Kairevice, L.; Petrauskas, A.; Saladzinskas, Z.; Janciauskiene, R.; Gudaityte, J.; Lizdenis, P.; Svagzdys, S.; Tamelis, A.; et al. Preoperative conventional chemoradiotherapy versus short-course radiotherapy with delayed surgery for rectal cancer: results of a randomized controlled trial. *BMC Cancer* **2016**, *16*, 927, doi:10.1186/s12885-016-2959-9.

243. Lefevre, J.H.; Mineur, L.; Kotti, S.; Rullier, E.; Rouanet, P.; de Chaisemartin, C.; Meunier, B.; Mehrdad, J.; Cotte, E.; Desrame, J.; et al. Effect of Interval (7 or 11 weeks) Between Neoadjuvant Radiochemotherapy and Surgery on Complete Pathologic Response in Rectal Cancer: A Multicenter, Randomized, Controlled Trial (GRECCAR-6). *Journal of clinical oncology : official journal of the American Society of Clinical Oncology* **2016**, *34*, 3773-3780, doi:10.1200/jco.2016.67.6049.

244. McLachlan, S.A.; Fisher, R.J.; Zalcberg, J.; Solomon, M.; Burmeister, B.; Goldstein, D.; Leong, T.; Ackland, S.P.; McKendrick, J.; McClure, B.; et al. The impact on health-related quality of life in the first 12 months: A randomised comparison of preoperative short-course radiation versus long-course chemoradiation for T3 rectal cancer (Trans-Tasman Radiation Oncology Group Trial 01.04). *Eur J Cancer* **2016**, *55*, 15-26, doi:10.1016/j.ejca.2015.10.060.

245. Nahas, S.C.; Rizkallah Nahas, C.S.; Sparapan Marques, C.F.; Ribeiro, U., Jr.; Cotti, G.C.; Imperiale, A.R.; Capareli, F.C.; Chih Chen, A.T.; Hoff, P.M.; Cecconello, I. Pathologic Complete Response in Rectal Cancer: Can We Detect It? Lessons Learned From a Proposed Randomized Trial of Watch-and-Wait Treatment of Rectal Cancer. *Dis Colon Rectum* **2016**, *59*, 255-263, doi:10.1097/dcr.0000000000000558.

246. Qin, Q.; Ma, T.; Deng, Y.; Zheng, J.; Zhou, Z.; Wang, H.; Wang, L.; Wang, J. Impact of Preoperative Radiotherapy on Anastomotic Leakage and Stenosis After Rectal Cancer Resection: Post Hoc Analysis of a Randomized Controlled Trial. *Dis Colon Rectum* **2016**, *59*, 934-942, doi:10.1097/dcr.0000000000000665.

247. Rosati, G.; Ambrosini, G.; Barni, S.; Andreoni, B.; Corradini, G.; Luchena, G.; Daniele, B.; Gaion, F.; Oliverio, G.; Duro, M.; et al. A randomized trial of intensive versus minimal surveillance of patients with resected Dukes B2-C colorectal carcinoma. *Ann Oncol* **2016**, *27*, 274-280, doi:10.1093/annonc/mdv541.

248. Saito, S.; Fujita, S.; Mizusawa, J.; Kanemitsu, Y.; Saito, N.; Kinugasa, Y.; Akazai, Y.; Ota, M.; Ohue, M.; Komori, K.; et al. Male sexual dysfunction after rectal cancer surgery: Results of a randomized trial comparing mesorectal excision with and without lateral lymph node dissection for patients with lower rectal cancer: Japan Clinical Oncology Group Study JCOG0212. *European journal of surgical oncology : the journal of the European Society of Surgical Oncology and the British Association of Surgical Oncology* **2016**, *42*, 1851-1858, doi:10.1016/j.ejso.2016.07.010.

249. Sclafani, F.; Chau, I.; Cunningham, D.; Lampis, A.; Hahne, J.C.; Ghidini, M.; Lote, H.; Zito, D.; Tabernero, J.; Glimelius, B.; et al. Sequence variation in mature microRNA-608 and benefit from neo-adjuvant treatment in locally advanced rectal cancer patients. *Carcinogenesis* **2016**, *37*, 852-857, doi:10.1093/carcin/bgw073.

250. Shi, L.; Li, X.; Pei, H.; Zhao, J.; Qiang, W.; Wang, J.; Xu, B.; Chen, L.; Wu, J.; Ji, M.; et al. Phase II study of computed tomography-guided (125)I-seed implantation plus chemotherapy for locally recurrent rectal cancer. *Radiother Oncol* **2016**, *118*, 375-381, doi:10.1016/j.radonc.2015.10.026.

251. Wiltink, L.M.; Marijnen, C.A.; Meershoek-Klein Kranenbarg, E.; van de Velde, C.J.; Nout, R.A. A comprehensive longitudinal overview of health-related quality of life and symptoms after treatment for rectal cancer in the TME trial. *Acta Oncol* **2016**, *55*, 502-508, doi:10.3109/0284186x.2015.1088171.

252. Wiśniowska, K.; Nasierowska-Guttmejer, A.; Polkowski, W.; Michalski, W.; Wyrwicz, L.; Pietrzak, L.; Rutkowski, A.; Malinowska, M.; Kryński, J.; Kosakowska, E.; et al. Does the addition of oxaliplatin to preoperative chemoradiation benefit cT4 or fixed cT3 rectal cancer treatment? A subgroup analysis from a prospective study. *European journal of surgical oncology : the journal of the European Society of Surgical Oncology and the British Association of Surgical Oncology* **2016**, *42*, 1859-1865, doi:10.1016/j.ejso.2016.08.001.

253. Bianco, F.; Romano, G.; Tsarkov, P.; Stanojevic, G.; Shroyer, K.; Giuratrabocchetta, S.; Bergamaschi, R. Extralevator with vs nonextralevator abdominoperineal excision for rectal cancer: the RELAPe randomized controlled trial. *Colorectal disease : the official journal of the Association of Coloproctology of Great Britain and Ireland* **2017**, *19*, 148-157, doi:10.1111/codi.13436.

254. Cotte, E.; Passot, G.; Decullier, E.; Maurice, C.; Glehen, O.; François, Y.; Lorchel, F.; Chapet, O.; Gerard, J.P. Pathologic Response, When Increased by Longer Interval, Is a Marker but Not the Cause of Good Prognosis in Rectal Cancer: 17-year Follow-up of the Lyon R90-01 Randomized Trial. *Int J Radiat Oncol Biol Phys* **2016**, *94*, 544-553, doi:10.1016/j.ijrobp.2015.10.061.

255. Foster, J.D.; Ewings, P.; Falk, S.; Cooper, E.J.; Roach, H.; West, N.P.; Williams-Yesson, B.A.; Hanna, G.B.; Francis, N.K. Surgical timing after chemoradiotherapy for rectal cancer, analysis of technique (STARRCAT): results of a feasibility multi-centre randomized controlled trial. *Tech Coloproctol* **2016**, *20*, 683-693, doi:10.1007/s10151-016-1514-7.

256. Kairevičė, L.; Latkauskas, T.; Tamelis, A.; Petrauskas, A.; Paužas, H.; Žvirblis, T.; Jaruševičius, L.; Saladžinskas, Ž.; Pavalkis, D.; Jančiauskienė, R. Preoperative long-course chemoradiotherapy plus adjuvant chemotherapy versus short-course radiotherapy without adjuvant chemotherapy both with delayed surgery for stage II-III resectable rectal cancer: 5-Year survival data of a randomized controlled trial. *Medicina (Kaunas)* **2017**, *53*, 150-158, doi:10.1016/j.medici.2017.05.006.

257. Ansari, N.; Solomon, M.J.; Fisher, R.J.; Mackay, J.; Burmeister, B.; Ackland, S.; Heriot, A.; Joseph, D.; McLachlan, S.A.; McClure, B.; et al. Acute Adverse Events and Postoperative Complications in a Randomized Trial of Preoperative Short-course Radiotherapy Versus Long-course Chemoradiotherapy for T3 Adenocarcinoma of the Rectum: Trans-Tasman Radiation Oncology Group Trial (TROG 01.04). *Annals of surgery* **2017**, *265*, 882-888, doi:10.1097/sla.0000000000001987.

258. Azria, D.; Doyen, J.; Jarlier, M.; Martel-Lafay, I.; Hennequin, C.; Etienne, P.; Vendrely, V.; François, E.; de La Roche, G.; Bouché, O.; et al. Late toxicities and clinical outcome at 5 years of the ACCORD 12/0405-PRODIGE 02 trial comparing two neoadjuvant chemoradiotherapy regimens for intermediate-risk rectal cancer. *Ann Oncol* **2017**, *28*, 2436-2442, doi:10.1093/annonc/mdx351.

259. Erlandsson, J.; Holm, T.; Pettersson, D.; Berglund, Å.; Cedermark, B.; Radu, C.; Johansson, H.; Machado, M.; Hjern, F.; Hallböök, O.; et al. Optimal fractionation of preoperative radiotherapy and timing to surgery for rectal cancer (Stockholm III): a multicentre, randomised, non-blinded, phase 3, non-inferiority trial. *The Lancet. Oncology* **2017**, *18*, 336-346, doi:10.1016/s1470-2045(17)30086-4.

260. Fokas, E.; Ströbel, P.; Fietkau, R.; Ghadimi, M.; Liersch, T.; Grabenbauer, G.G.; Hartmann, A.; Kaufmann, M.; Sauer, R.; Graeven, U.; et al. Tumor Regression Grading After Preoperative Chemoradiotherapy as a Prognostic Factor and Individual-Level Surrogate for Disease-Free Survival in Rectal Cancer. *Journal of the National Cancer Institute* **2017**, *109*, doi:10.1093/jnci/djx095.

261. Haddad, P.; Miraie, M.; Farhan, F.; Fazeli, M.S.; Alikhassi, A.; Maddah-Safaei, A.; Aghili, M.; Kalaghchi, B.; Babaei, M. Addition of oxaliplatin to neoadjuvant radiochemotherapy in MRI-defined T3, T4 or N+ rectal cancer: a randomized clinical trial. *Asia Pac J Clin Oncol* **2017**, *13*, 416-422, doi:10.1111/ajco.12675.

262. Rotovnik Kozjek, N.; Kompan, L.; Žagar, T.; Mrevlje, Ž. Influence of enteral glutamine on inflammatory and hormonal response in patients with rectal cancer during preoperative radiochemotherapy. *Eur J Clin Nutr* **2017**, *71*, 671-673, doi:10.1038/ejcn.2017.11.

263. Moore, J.; Price, T.; Carruthers, S.; Selva-Nayagam, S.; Luck, A.; Thomas, M.; Hewett, P. Prospective randomized trial of neoadjuvant chemotherapy during the 'wait period' following preoperative chemoradiotherapy for rectal cancer: results of the WAIT trial. *Colorectal disease : the official journal of the Association of Coloproctology of Great Britain and Ireland* **2017**, *19*, 973-979, doi:10.1111/codi.13724.

264. Musters, G.D.; Klaver, C.E.L.; Bosker, R.J.I.; Burger, J.W.A.; van Duijvendijk, P.; van Etten, B.; van Geloven, A.A.W.; de Graaf, E.J.R.; Hoff, C.; Leijtens, J.W.A.; et al. Biological Mesh Closure of the Pelvic Floor After Extralevator Abdominoperineal Resection for Rectal Cancer: A Multicenter Randomized Controlled Trial (the BIOPEX-study). *Annals of surgery* **2017**, *265*, 1074-1081, doi:10.1097/sla.0000000000002020.

265. Rouanet, P.; Rullier, E.; Lelong, B.; Maingon, P.; Tuech, J.J.; Pezet, D.; Castan, F.; Nougaret, S. Tailored Treatment Strategy for Locally Advanced Rectal Carcinoma Based on the Tumor Response to Induction Chemotherapy: Preliminary Results of the French Phase II Multicenter GRECCAR4 Trial. *Dis Colon Rectum* **2017**, *60*, 653-663, doi:10.1097/dcr.0000000000000849.

266. Rullier, E.; Rouanet, P.; Tuech, J.J.; Valverde, A.; Lelong, B.; Rivoire, M.; Faucheron, J.L.; Jafari, M.; Portier, G.; Meunier, B.; et al. Organ preservation for rectal cancer (GRECCAR 2): a prospective, randomised, open-label, multicentre, phase 3 trial. *Lancet* **2017**, *390*, 469-479, doi:10.1016/s0140-6736(17)31056-5.

267. Seshadri, R.A.; West, N.P.; Sundersingh, S. A pilot randomized study comparing extralevator with conventional abdominoperineal excision for low rectal cancer after neoadjuvant chemoradiation. *Colorectal disease : the official journal of the Association of Coloproctology of Great Britain and Ireland* **2017**, *19*, O253-o262, doi:10.1111/codi.13726.

268. Singh, K.; Gupta, M.K.; Seam, R.K.; Gupta, M. A prospective randomized trial comparing capecitabine-based chemoradiotherapy with 5-FU-based chemoradiotherapy in neoadjuvant setting in locally advanced carcinoma rectum. *Indian J Cancer* **2017**, *54*, 347-351, doi:10.4103/ijc.IJC_174_17.

269. Rutkowski, A.; Pietrzak, L.; Kryński, J.; Zając, L.; Bednarczyk, M.; Olesiński, T.; Szpakowski, M.; Saramak, P.; Pierzankowski, I.; Hevelke, P.; et al. The gentamicin-collagen implant and the risk of distant metastases of rectal cancer following short-course radiotherapy and curative resection: the long-term outcomes of a randomized study. *International journal of colorectal disease* **2018**, *33*, 1087-1096, doi:10.1007/s00384-018-3045-3.

270. Sprenger, T.; Beißbarth, T.; Sauer, R.; Tschmelitsch, J.; Fietkau, R.; Liersch, T.; Hohenberger, W.; Staib, L.; Gaedcke, J.; Raab, H.R.; et al. Long-term prognostic impact of surgical complications in the German Rectal Cancer Trial CAO/ARO/AIO-94. *The British journal of surgery* **2018**, *105*, 1510-1518, doi:10.1002/bjs.10877.

271. von den Grün, J.M.; Hartmann, A.; Fietkau, R.; Ghadimi, M.; Liersch, T.; Hohenberger, W.; Weitz, J.; Sauer, R.; Wittekind, C.; Ströbel, P.; et al. Can clinicopathological parameters predict for lymph node metastases in ypT0-2 rectal carcinoma? Results of the CAO/ARO/AIO-94 and CAO/ARO/AIO-04 phase 3 trials. *Radiother Oncol* **2018**, *128*, 557-563, doi:10.1016/j.radonc.2018.06.008.

272. Akgun, E.; Caliskan, C.; Bozbiyik, O.; Yoldas, T.; Sezak, M.; Ozkok, S.; Kose, T.; Karabulut, B.; Harman, M.; Ozutemiz, O. Randomized clinical trial of short or long interval between neoadjuvant chemoradiotherapy and surgery for rectal cancer. *The British journal of surgery* **2018**, *105*, 1417-1425, doi:10.1002/bjs.10984.

273. Dias, A.R.; Pereira, M.A.; de Mello, E.S.; Nahas, S.C.; Cecconello, I.; Ribeiro, U., Jr. Lymph Node Yield After Neoadjuvant Chemoradiotherapy in Rectal Cancer Specimens: A Randomized Trial Comparing Two Fixatives. *Dis Colon Rectum* **2018**, *61*, 888-896, doi:10.1097/dcr.0000000000001097.

274. Fokas, E.; Fietkau, R.; Hartmann, A.; Hohenberger, W.; Grützmann, R.; Ghadimi, M.; Liersch, T.; Ströbel, P.; Grabenbauer, G.G.; Graeven, U.; et al. Neoadjuvant rectal score as individual-level surrogate for disease-free survival in rectal cancer in the CAO/ARO/AIO-04 randomized phase III trial. *Ann Oncol* **2018**, *29*, 1521-1527, doi:10.1093/annonc/mdy143.

275. Hofheinz, R.D.; Arnold, D.; Fokas, E.; Kaufmann, M.; Hothorn, T.; Folprecht, G.; Fietkau, R.; Hohenberger, W.; Ghadimi, M.; Liersch, T.; et al. Impact of age on the efficacy of oxaliplatin in the preoperative chemoradiotherapy and adjuvant chemotherapy of rectal cancer: a post hoc analysis of the CAO/ARO/AIO-04 phase III trial. *Ann Oncol* **2018**, *29*, 1793-1799, doi:10.1093/annonc/mdy205.

276. Kim, S.Y.; Joo, J.; Kim, T.W.; Hong, Y.S.; Kim, J.E.; Hwang, I.G.; Kim, B.G.; Lee, K.W.; Kim, J.W.; Oh, H.S.; et al. A Randomized Phase 2 Trial of Consolidation Chemotherapy After Preoperative Chemoradiation Therapy Versus Chemoradiation Therapy Alone for Locally Advanced Rectal Cancer: KCSG CO 14-03. *Int J Radiat Oncol Biol Phys* **2018**, *101*, 889-899, doi:10.1016/j.ijrobp.2018.04.013.

277. Okada, K.; Sadahiro, S.; Ogimi, T.; Miyakita, H.; Saito, G.; Tanaka, A.; Suzuki, T. Tattooing improves the detection of small lymph nodes and increases the number of retrieved lymph nodes in patients with rectal cancer who receive preoperative chemoradiotherapy: A randomized controlled clinical trial. *American journal of surgery* **2018**, *215*, 563-569, doi:10.1016/j.amjsurg.2017.06.030.

278. Qi, F.; Zheng, Z.; Yan, Q.; Liu, J.; Chen, Y.; Zhang, G. Comparisons of Efficacy, Safety, and Cost of Chemotherapy Regimens FOLFOX4 and FOLFIRINOX in Rectal Cancer: A Randomized, Multicenter Study. *Med Sci Monit* **2018**, *24*, 1970-1979, doi:10.12659/msm.906934.

279. Sclafani, F.; Chau, I.; Cunningham, D.; Hahne, J.C.; Vlachogiannis, G.; Eltahir, Z.; Lampis, A.; Braconi, C.; Kalaitzaki, E.; De Castro, D.G.; et al. KRAS and BRAF mutations in circulating tumour DNA from locally advanced rectal cancer. *Sci Rep* **2018**, *8*, 1445, doi:10.1038/s41598-018-19212-5.

280. Wawok, P.; Polkowski, W.; Richter, P.; Szczepkowski, M.; Olędzki, J.; Wierzbicki, R.; Gach, T.; Rutkowski, A.; Dziki, A.; Kołodziejski, L.; et al. Preoperative radiotherapy and local excision of rectal cancer: Long-term results of a randomised study. *Radiother Oncol* **2018**, *127*, 396-403, doi:10.1016/j.radonc.2018.04.004.

281. Zhu, J.; Li, X.; Shen, Y.; Guan, Y.; Gu, W.; Lian, P.; Sheng, W.; Cai, S.; Zhang, Z. Genotype-driven phase I study of weekly irinotecan in combination with capecitabine-based neoadjuvant chemoradiation for locally advanced rectal cancer. *Radiother Oncol* **2018**, *129*, 143-148, doi:10.1016/j.radonc.2017.11.026.

282. Hu, H.; Huang, J.; Lan, P.; Wang, L.; Huang, M.; Wang, J.; Deng, Y. CEA clearance pattern as a predictor of tumor response to neoadjuvant treatment in rectal cancer: a post-hoc analysis of FOWARC trial. *BMC Cancer* **2018**, *18*, 1145, doi:10.1186/s12885-018-4997-y.

283. Wang, F.; Fan, W.; Peng, J.; Lu, Z.; Pan, Z.; Li, L.; Gao, Y.; Li, H.; Chen, G.; Wu, X.; et al. Total mesorectal excision with or without preoperative chemoradiotherapy for resectable mid/low rectal cancer: a long-term analysis of a prospective, single-center, randomized trial. *Cancer Commun (Lond)* **2018**, *38*, 73, doi:10.1186/s40880-018-0342-8.

284. Kitz, J.; Fokas, E.; Beissbarth, T.; Ströbel, P.; Wittekind, C.; Hartmann, A.; Rüschoff, J.; Papadopoulos, T.; Rösler, E.; Ortloff-Kittredge, P.; et al. Association of Plane of Total Mesorectal Excision With Prognosis of Rectal Cancer: Secondary Analysis of the CAO/ARO/AIO-04 Phase 3 Randomized Clinical Trial. *JAMA Surg* **2018**, *153*, e181607, doi:10.1001/jamasurg.2018.1607.

285. Fiori, E.; Crocetti, D.; Lamazza, A.; F, D.E.F.; Tarallo, M.; Sterpetti, A.V.; Mingoli, A.; Sapienza, P.; G, D.E.T. Resection or Stenting in the Treatment of Symptomatic Advanced Metastatic Rectal Cancer: A Dilemma. *Anticancer Res* **2019**, *39*, 6781-6786, doi:10.21873/anticanres.13893.

286. Diefenhardt, M.; Hofheinz, R.D.; Martin, D.; Beißbarth, T.; Arnold, D.; Hartmann, A.; von der Grün, J.; Grützmann, R.; Liersch, T.; Ströbel, P.; et al. Leukocytosis and neutrophilia as independent prognostic immunological biomarkers for clinical outcome in the CAO/ARO/AIO-04 randomized phase 3 rectal cancer trial. *International journal of cancer. Journal international du cancer* **2019**, *145*, 2282-2291, doi:10.1002/ijc.32274.

287. Ciseł, B.; Pietrzak, L.; Michalski, W.; Wyrwicz, L.; Rutkowski, A.; Kosakowska, E.; Cencelewicz, A.; Spałek, M.; Polkowski, W.; Jankiewicz, M.; et al. Long-course preoperative chemoradiation versus 5 × 5 Gy and consolidation chemotherapy for clinical T4 and fixed clinical T3 rectal cancer: long-term results of the randomized Polish II study. *Ann Oncol* **2019**, *30*, 1298-1303, doi:10.1093/annonc/mdz186.

288. Erlandsson, J.; Lörinc, E.; Ahlberg, M.; Pettersson, D.; Holm, T.; Glimelius, B.; Martling, A. Tumour regression after radiotherapy for rectal cancer - Results from the randomised Stockholm III trial. *Radiother Oncol* **2019**, *135*, 178-186, doi:10.1016/j.radonc.2019.03.016.

289. Erlandsson, J.; Pettersson, D.; Glimelius, B.; Holm, T.; Martling, A. Postoperative complications in relation to overall treatment time in patients with rectal cancer receiving neoadjuvant radiotherapy. *The British journal of surgery* **2019**, *106*, 1248-1256, doi:10.1002/bjs.11200.

290. Fokas, E.; Allgäuer, M.; Polat, B.; Klautke, G.; Grabenbauer, G.G.; Fietkau, R.; Kuhnt, T.; Staib, L.; Brunner, T.; Grosu, A.L.; et al. Randomized Phase II Trial of Chemoradiotherapy Plus Induction or Consolidation Chemotherapy as Total Neoadjuvant Therapy for Locally Advanced Rectal Cancer: CAO/ARO/AIO-12. *Journal of clinical oncology : official journal of the American Society of Clinical Oncology* **2019**, *37*, 3212-3222, doi:10.1200/jco.19.00308.

291. Nougaret, S.; Castan, F.; de Forges, H.; Vargas, H.A.; Gallix, B.; Gourgou, S.; Rouanet, P. Early MRI predictors of disease-free survival in locally advanced rectal cancer from the GRECCAR 4 trial. *The British journal of surgery* **2019**, *106*, 1530-1541, doi:10.1002/bjs.11233.

292. Moug, S.J.; Mutrie, N.; Barry, S.J.E.; Mackay, G.; Steele, R.J.C.; Boachie, C.; Buchan, C.; Anderson, A.S. Prehabilitation is feasible in patients with rectal cancer undergoing neoadjuvant chemoradiotherapy and may minimize physical deterioration: results from the REx trial. *Colorectal disease : the official journal of the Association of Coloproctology of Great Britain and Ireland* **2019**, *21*, 548-562, doi:10.1111/codi.14560.

293. Hong, Y.S.; Kim, S.Y.; Lee, J.S.; Nam, B.H.; Kim, K.P.; Kim, J.E.; Park, Y.S.; Park, J.O.; Baek, J.Y.; Kim, T.Y.; et al. Oxaliplatin-Based Adjuvant Chemotherapy for Rectal Cancer After Preoperative Chemoradiotherapy (ADORE): Long-Term Results of a Randomized Controlled Trial. *Journal of clinical oncology : official journal of the American Society of Clinical Oncology* **2019**, *37*, 3111-3123, doi:10.1200/jco.19.00016.

294. Borg, C.; Mantion, G.; Boudghène, F.; Mornex, F.; Ghiringhelli, F.; Adenis, A.; Azria, D.; Balosso, J.; Ben Abdelghani, M.; Bachet, J.B.; et al. Efficacy and Safety of Two Neoadjuvant Strategies With Bevacizumab in MRI-Defined Locally Advanced T3 Resectable Rectal Cancer: Final Results of a Randomized, Noncomparative Phase 2 INOVA Study. *Clin Colorectal Cancer* **2019**, *18*, 200-208.e201, doi:10.1016/j.clcc.2019.04.006.

295. van der Valk, M.J.M.; Hilling, D.E.; Meershoek-Klein Kranenbarg, E.; Peeters, K.; Kapiteijn, E.; Tsonaka, R.; van de Velde, C.J.H.; Marang-van de Mheen, P.J. Quality of Life After Curative Resection for Rectal Cancer in Patients Treated With Adjuvant Chemotherapy Compared With Observation: Results of the Randomized Phase III SCRIPT Trial. *Dis Colon Rectum* **2019**, *62*, 711-720, doi:10.1097/dcr.0000000000001336.

296. Lefèvre, J.H.; Mineur, L.; Cachanado, M.; Denost, Q.; Rouanet, P.; de Chaisemartin, C.; Meunier, B.; Mehrdad, J.; Cotte, E.; Desrame, J.; et al. Does A Longer Waiting Period After Neoadjuvant Radio-chemotherapy Improve the Oncological Prognosis of Rectal Cancer?: Three Years' Follow-up Results of the Greccar-6 Randomized Multicenter Trial. *Annals of surgery* **2019**, *270*, 747-754, doi:10.1097/sla.0000000000003530.

297. Deng, Y.; Chi, P.; Lan, P.; Wang, L.; Chen, W.; Cui, L.; Chen, D.; Cao, J.; Wei, H.; Peng, X.; et al. Neoadjuvant Modified FOLFOX6 With or Without Radiation Versus Fluorouracil Plus Radiation for Locally Advanced Rectal Cancer: Final Results of the Chinese FOWARC Trial. *Journal of clinical oncology : official journal of the American Society of Clinical Oncology* **2019**, *37*, 3223-3233, doi:10.1200/jco.18.02309.

298. Wang, S.; Wen, F.; Zhang, P.; Wang, X.; Li, Q. Cost-effectiveness analysis of long-course oxaliplatin and bolus of fluorouracil based preoperative chemoradiotherapy vs. 5x5Gy radiation plus FOLFOX4 for locally advanced resectable rectal cancer. *Radiat Oncol* **2019**, *14*, 113, doi:10.1186/s13014-019-1319-8.

299. Wang, J.; Guan, Y.; Gu, W.; Yan, S.; Zhou, J.; Huang, D.; Tong, T.; Li, C.; Cai, S.; Zhang, Z.; et al. Long-course neoadjuvant chemoradiotherapy with versus without a concomitant boost in locally advanced rectal cancer: a randomized, multicenter, phase II trial (FDRT-002). *Radiat Oncol* **2019**, *14*, 215, doi:10.1186/s13014-019-1420-z.

300. Sun, W.; Dou, R.; Chen, J.; Lai, S.; Zhang, C.; Ruan, L.; Kang, L.; Deng, Y.; Lan, P.; Wang, L.; et al. Impact of Long-Course Neoadjuvant Radiation on Postoperative Low Anterior Resection Syndrome and Quality of Life in Rectal Cancer: Post Hoc Analysis of a Randomized Controlled Trial. *Annals of surgical oncology* **2019**, *26*, 746-755, doi:10.1245/s10434-018-07096-8.

301. Valentini, V.; Gambacorta, M.A.; Cellini, F.; Aristei, C.; Coco, C.; Barbaro, B.; Alfieri, S.; D'Ugo, D.; Persiani, R.; Deodato, F.; et al. The INTERACT Trial: Long-term results of a randomised trial on preoperative capecitabine-based radiochemotherapy intensified by concomitant boost or oxaliplatin, for cT2 (distal)-cT3 rectal cancer. *Radiother Oncol* **2019**, *134*, 110-118, doi:10.1016/j.radonc.2018.11.023.

302. Chakravarthy, A.B.; Zhao, F.; Meropol, N.J.; Flynn, P.J.; Wagner, L.I.; Sloan, J.; Diasio, R.B.; Mitchell, E.P.; Catalano, P.; Giantonio, B.J.; et al. Intergroup Randomized Phase III Study of Postoperative Oxaliplatin, 5-Fluorouracil, and Leucovorin Versus Oxaliplatin, 5-Fluorouracil, Leucovorin, and Bevacizumab for Patients with Stage II or III Rectal Cancer Receiving Preoperative Chemoradiation: A Trial of the ECOG-ACRIN Research Group (E5204). *Oncologist* **2020**, *25*, e798-e807, doi:10.1634/theoncologist.2019-0437.

303. Rullier, E.; Vendrely, V.; Asselineau, J.; Rouanet, P.; Tuech, J.J.; Valverde, A.; de Chaisemartin, C.; Rivoire, M.; Trilling, B.; Jafari, M.; et al. Organ preservation with chemoradiotherapy plus local excision for rectal cancer: 5-year results of the GRECCAR 2 randomised trial. *Lancet Gastroenterol Hepatol* **2020**, *5*, 465-474, doi:10.1016/s2468-1253(19)30410-8.

304. Masaki, T.; Matsuoka, H.; Kishiki, T.; Kojima, K.; Tonari, A.; Aso, N.; Beniya, A.; Iioka, A.; Wakamatsu, T.; Sunami, E. Changing Patterns of Distant Metastasis in Patients With Lower Rectal Cancer Undergoing Intraoperative Radiotherapy. *In Vivo* **2020**, *34*, 3655-3659, doi:10.21873/invivo.12212.

305. Terzi, C.; Bingul, M.; Arslan, N.C.; Ozturk, E.; Canda, A.E.; Isik, O.; Yilmazlar, T.; Obuz, F.; Birkay Gorken, I.; Kurt, M.; et al. Randomized controlled trial of 8 weeks' vs 12 weeks' interval between neoadjuvant chemoradiotherapy and surgery for locally advanced rectal cancer. *Colorectal disease : the official journal of the Association of Coloproctology of Great Britain and Ireland* **2020**, *22*, 279-288, doi:10.1111/codi.14867.

306. Deng, X.; Liu, P.; Jiang, D.; Wei, M.; Wang, X.; Yang, X.; Zhang, Y.; Wu, B.; Liu, Y.; Qiu, M.; et al. Neoadjuvant Radiotherapy Versus Surgery Alone for Stage II/III Mid-low Rectal Cancer With or Without High-risk Factors: A Prospective Multicenter Stratified Randomized Trial. *Annals of surgery* **2020**, *272*, 1060-1069, doi:10.1097/sla.0000000000003649.

307. van den Ende, R.P.J.; Peters, F.P.; Harderwijk, E.; Rütten, H.; Bouwmans, L.; Berbee, M.; Canters, R.A.M.; Stoian, G.; Compagner, K.; Rozema, T.; et al. Radiotherapy quality assurance for mesorectum treatment planning within the multi-center phase II STAR-TReC trial: Dutch results. *Radiat Oncol* **2020**, *15*, 41, doi:10.1186/s13014-020-01487-6.

308. van der Valk, M.J.M.; Marijnen, C.A.M.; van Etten, B.; Dijkstra, E.A.; Hilling, D.E.; Kranenbarg, E.M.; Putter, H.; Roodvoets, A.G.H.; Bahadoer, R.R.; Fokstuen, T.; et al. Compliance and tolerability of short-course radiotherapy followed by preoperative chemotherapy and surgery for high-risk rectal cancer - Results of the international randomized RAPIDO-trial. *Radiother Oncol* **2020**, *147*, 75-83, doi:10.1016/j.radonc.2020.03.011.

309. Couwenberg, A.M.; Burbach, J.P.M.; Berbee, M.; Lacle, M.M.; Arensman, R.; Raicu, M.G.; Wessels, F.J.; Verdult, J.; Roodhart, J.; Reerink, O.; et al. Efficacy of Dose-Escalated Chemoradiation on Complete Tumor Response in Patients with Locally Advanced Rectal Cancer (RECTAL-BOOST): A Phase 2 Randomized Controlled Trial. *Int J Radiat Oncol Biol Phys* **2020**, *108*, 1008-1018, doi:10.1016/j.ijrobp.2020.06.013.

310. Diefenhardt, M.; Ludmir, E.B.; Hofheinz, R.D.; Ghadimi, M.; Minsky, B.D.; Rödel, C.; Fokas, E. Association of Treatment Adherence With Oncologic Outcomes for Patients With Rectal Cancer: A Post Hoc Analysis of the CAO/ARO/AIO-04 Phase 3 Randomized Clinical Trial. *JAMA Oncol* **2020**, *6*, 1416-1421, doi:10.1001/jamaoncol.2020.2394.

311. Sprenger, T.; Beißbarth, T.; Sauer, R.; Tschmelitsch, J.; Fietkau, R.; Hohenberger, W.; Staib, L.; Raab, H.R.; Rödel, C.; Ghadimi, M. The long-term influence of hospital and surgeon volume on local control and survival in the randomized German Rectal Cancer Trial CAO/ARO/AIO-94. *Surg Oncol* **2020**, *35*, 200-205, doi:10.1016/j.suronc.2020.08.021.

312. Zhu, J.; Liu, A.; Sun, X.; Liu, L.; Zhu, Y.; Zhang, T.; Jia, J.; Tan, S.; Wu, J.; Wang, X.; et al. Multicenter, Randomized, Phase III Trial of Neoadjuvant Chemoradiation With Capecitabine and Irinotecan Guided by UGT1A1 Status in Patients With Locally Advanced Rectal Cancer. *Journal of clinical oncology : official journal of the American Society of Clinical Oncology* **2020**, *38*, 4231-4239, doi:10.1200/jco.20.01932.

313. Masaki, T.; Matsuoka, H.; Kishiki, T.; Kojima, K.; Aso, N.; Beniya, A.; Tonari, A.; Takayama, M.; Abe, N.; Sunami, E. Intraoperative radiotherapy for resectable advanced lower rectal cancer-final results of a randomized controlled trial (UMIN000021353). *Langenbecks Arch Surg* **2020**, *405*, 247-254, doi:10.1007/s00423-020-01875-2.

314. Salazar, R.; Capdevila, J.; Manzano, J.L.; Pericay, C.; Martínez-Villacampa, M.; López, C.; Losa, F.; Safont, M.J.; Gómez-España, A.; Alonso-Orduña, V.; et al. Phase II randomized trial of capecitabine with bevacizumab and external beam radiation therapy as preoperative treatment for patients with resectable locally advanced rectal adenocarcinoma: long term results. *BMC Cancer* **2020**, *20*, 1164, doi:10.1186/s12885-020-07661-z.

315. Bach, S.P.; Gilbert, A.; Brock, K.; Korsgen, S.; Geh, I.; Hill, J.; Gill, T.; Hainsworth, P.; Tutton, M.G.; Khan, J.; et al. Radical surgery versus organ preservation via short-course radiotherapy followed by transanal endoscopic microsurgery for early-stage rectal cancer (TREC): a randomised, open-label feasibility study. *Lancet Gastroenterol Hepatol* **2021**, *6*, 92-105, doi:10.1016/s2468-1253(20)30333-2.

316. Schmoll, H.J.; Stein, A.; Van Cutsem, E.; Price, T.; Hofheinz, R.D.; Nordlinger, B.; Daisne, J.F.; Janssens, J.; Brenner, B.; Reinel, H.; et al. Pre- and Postoperative Capecitabine Without or With Oxaliplatin in Locally Advanced Rectal Cancer: PETACC 6 Trial by EORTC GITCG and ROG, AIO, AGITG, BGDO, and FFCD. *Journal of clinical oncology : official journal of the American Society of Clinical Oncology* **2021**, *39*, 17-29, doi:10.1200/jco.20.01740.

317. Rouanet, P.; Rivoire, M.; Gourgou, S.; Lelong, B.; Rullier, E.; Jafari, M.; Mineur, L.; Pocard, M.; Faucheron, J.L.; Dravet, F.; et al. Sphincter-saving surgery for ultra-low rectal carcinoma initially indicated for abdominoperineal resection: Is it safe on a long-term follow-up? *J Surg Oncol* **2021**, *123*, 299-310, doi:10.1002/jso.26249.

318. Monteil, J.; Le Brun-Ly, V.; Cachin, F.; Zasadny, X.; Seitz, J.F.; Mundler, O.; Selvy, M.; Smith, D.; Rullier, E.; Lavau-Denes, S.; et al. Comparison of 18FDG-PET/CT and conventional follow-up methods in colorectal cancer: A randomised prospective study. *Dig Liver Dis* **2021**, *53*, 231-237, doi:10.1016/j.dld.2020.10.012.

319. Garant, A.; Kavan, P.; Martin, A.G.; Azoulay, L.; Vendrely, V.; Lavoie, C.; Vasilevsky, C.A.; Boutros, M.; Faria, J.; Nguyen, T.N.; et al. Optimizing treatment sequencing of chemotherapy for patients with rectal cancer: The KIR randomized phase II trial. *Radiother Oncol* **2021**, *155*, 237-245, doi:10.1016/j.radonc.2020.11.008.

320. Bahadoer, R.R.; Dijkstra, E.A.; van Etten, B.; Marijnen, C.A.M.; Putter, H.; Kranenbarg, E.M.; Roodvoets, A.G.H.; Nagtegaal, I.D.; Beets-Tan, R.G.H.; Blomqvist, L.K.; et al. Short-course radiotherapy followed by chemotherapy before total mesorectal excision (TME) versus preoperative chemoradiotherapy, TME, and optional adjuvant chemotherapy in locally advanced rectal cancer (RAPIDO): a randomised, open-label, phase 3 trial. *The Lancet. Oncology* **2021**, *22*, 29-42, doi:10.1016/s1470-2045(20)30555-6.

321. Kosmala, R.; Fokas, E.; Flentje, M.; Sauer, R.; Liersch, T.; Graeven, U.; Fietkau, R.; Hohenberger, W.; Arnold, D.; Hofheinz, R.D.; et al. Quality of life in rectal cancer patients with or without oxaliplatin in the randomised CAO/ARO/AIO-04 phase 3 trial. *Eur J Cancer* **2021**, *144*, 281-290, doi:10.1016/j.ejca.2020.11.029.

322. Miwa, K.; Oki, E.; Enomoto, M.; Ihara, K.; Ando, K.; Fujita, F.; Tominaga, M.; Mori, S.; Nakayama, G.; Shimokawa, M.; et al. Randomized phase II study comparing the efficacy and safety of SOX versus mFOLFOX6 as neoadjuvant chemotherapy without radiotherapy for locally advanced rectal cancer (KSCC1301). *BMC Cancer* **2021**, *21*, 23, doi:10.1186/s12885-020-07766-5.

323. Chakrabarti, D.; Rajan, S.; Akhtar, N.; Qayoom, S.; Gupta, S.; Verma, M.; Srivastava, K.; Kumar, V.; Bhatt, M.L.B.; Gupta, R. Short-course radiotherapy with consolidation chemotherapy versus conventionally fractionated long-course chemoradiotherapy for locally advanced rectal cancer: randomized clinical trial. *The British journal of surgery* **2021**, *108*, 511-520, doi:10.1093/bjs/znab020.

324. Conroy, T.; Bosset, J.F.; Etienne, P.L.; Rio, E.; François, É.; Mesgouez-Nebout, N.; Vendrely, V.; Artignan, X.; Bouché, O.; Gargot, D.; et al. Neoadjuvant chemotherapy with FOLFIRINOX and preoperative chemoradiotherapy for patients with locally advanced rectal cancer (UNICANCER-PRODIGE 23): a multicentre, randomised, open-label, phase 3 trial. *The Lancet. Oncology* **2021**, *22*, 702-715, doi:10.1016/s1470-2045(21)00079-6.

325. Park, J.W.; Kang, S.B.; Hao, J.; Lim, S.B.; Choi, H.S.; Kim, D.W.; Chang, H.J.; Kim, D.Y.; Jung, K.H.; Kim, T.Y.; et al. Open versus laparoscopic surgery for mid or low rectal cancer after neoadjuvant chemoradiotherapy (COREAN trial): 10-year follow-up of an open-label, non-inferiority, randomised controlled trial. *Lancet Gastroenterol Hepatol* **2021**, *6*, 569-577, doi:10.1016/s2468-1253(21)00094-7.

326. Xie, Y.; Lin, J.; Wang, X.; Wang, P.; Zhuang, Z.; Zou, Q.; Cai, D.; Huang, Z.; Bai, L.; Tang, G.; et al. The Addition of Preoperative Radiation Is Insufficient for Lateral Pelvic Control in a Subgroup of Patients With Low Locally Advanced Rectal Cancer: A Post Hoc Study of a Randomized Controlled Trial. *Dis Colon Rectum* **2021**, *64*, 1321-1330, doi:10.1097/dcr.0000000000001935.

327. Morielli, A.R.; Usmani, N.; Boulé, N.G.; Severin, D.; Tankel, K.; Joseph, K.; Nijjar, T.; Fairchild, A.; Courneya, K.S. Feasibility, Safety, and Preliminary Efficacy of Exercise During and After Neoadjuvant Rectal Cancer Treatment: A Phase II Randomized Controlled Trial. *Clin Colorectal Cancer* **2021**, *20*, 216-226, doi:10.1016/j.clcc.2021.05.004.

328. Rahma, O.E.; Yothers, G.; Hong, T.S.; Russell, M.M.; You, Y.N.; Parker, W.; Jacobs, S.A.; Colangelo, L.H.; Lucas, P.C.; Gollub, M.J.; et al. Use of Total Neoadjuvant Therapy for Locally Advanced Rectal Cancer: Initial Results From the Pembrolizumab Arm of a Phase 2 Randomized Clinical Trial. *JAMA Oncol* **2021**, *7*, 1225-1230, doi:10.1001/jamaoncol.2021.1683.

329. Zhao, S.; Zhang, L.; Gao, F.; Wu, M.; Zheng, J.; Bai, L.; Li, F.; Liu, B.; Pan, Z.; Liu, J.; et al. Transanal Drainage Tube Use for Preventing Anastomotic Leakage After Laparoscopic Low Anterior Resection in Patients With Rectal Cancer: A Randomized Clinical Trial. *JAMA Surg* **2021**, *156*, 1151-1158, doi:10.1001/jamasurg.2021.4568.

330. Diefenhardt, M.; Ludmir, E.B.; Hofheinz, R.D.; Ghadimi, M.; Minsky, B.D.; Fleischmann, M.; Fokas, E.; Rödel, C. Impact of body-mass index on treatment and outcome in locally advanced rectal cancer: A secondary, post-hoc analysis of the CAO/ARO/AIO-04 randomized phase III trial. *Radiother Oncol* **2021**, *164*, 223-231, doi:10.1016/j.radonc.2021.09.028.

331. Pach, R.; Sierzega, M.; Szczepanik, A.; Popiela, T.; Richter, P. Preoperative radiotherapy 5 × 5 Gy and short versus long interval between surgery for resectable rectal cancer: 10-Year follow-up of the randomised controlled trial. *Radiother Oncol* **2021**, *164*, 268-274, doi:10.1016/j.radonc.2021.10.006.

332. Li, N.; Zhu, Y.; Liu, L.Y.; Feng, Y.R.; Wang, W.L.; Wang, J.; Wang, H.; Li, G.F.; Tang, Y.; Hu, C.; et al. Postoperative Chemoradiotherapy With Capecitabine and Oxaliplatin vs Capecitabine for Stage II to III Rectal Cancer: A Randomized Clinical Trial. *JAMA Netw Open* **2021**, *4*, e2136116, doi:10.1001/jamanetworkopen.2021.36116.

333. Blok, R.D.; Sharabiany, S.; Stoker, J.; Laan, E.T.M.; Bosker, R.J.I.; Burger, J.W.A.; Chaudhri, S.; van Duijvendijk, P.; van Etten, B.; van Geloven, A.A.W.; et al. Cumulative 5-year Results of a Randomized Controlled Trial Comparing Biological Mesh With Primary Perineal Wound Closure After Extralevator Abdominoperineal Resection (BIOPEX-study). *Annals of surgery* **2022**, *275*, e37-e44, doi:10.1097/sla.0000000000004763.

334. Verweij, M.E.; Hoendervangers, S.; Couwenberg, A.M.; Burbach, J.P.M.; Berbee, M.; Buijsen, J.; Roodhart, J.; Reerink, O.; Pronk, A.; Consten, E.C.J.; et al. Impact of Dose-Escalated Chemoradiation on Quality of Life in Patients With Locally Advanced Rectal Cancer: 2-Year Follow-Up of the Randomized RECTAL-BOOST Trial. *Int J Radiat Oncol Biol Phys* **2022**, *112*, 694-703, doi:10.1016/j.ijrobp.2021.09.052.

335. Rouanet, P.; Rullier, E.; Lelong, B.; Maingon, P.; Tuech, J.J.; Pezet, D.; Castan, F.; Nougaret, S. Tailored Strategy for Locally Advanced Rectal Carcinoma (GRECCAR 4): Long-term Results From a Multicenter, Randomized, Open-Label, Phase II Trial. *Dis Colon Rectum* **2022**, *65*, 986-995, doi:10.1097/dcr.0000000000002153.

336. Fokas, E.; Schlenska-Lange, A.; Polat, B.; Klautke, G.; Grabenbauer, G.G.; Fietkau, R.; Kuhnt, T.; Staib, L.; Brunner, T.; Grosu, A.L.; et al. Chemoradiotherapy Plus Induction or Consolidation Chemotherapy as Total Neoadjuvant Therapy for Patients With Locally Advanced Rectal Cancer: Long-term Results of the CAO/ARO/AIO-12 Randomized Clinical Trial. *JAMA Oncol* **2022**, *8*, e215445, doi:10.1001/jamaoncol.2021.5445.

337. Erlandsson, J.; Fuentes, S.; Radu, C.; Frödin, J.E.; Johansson, H.; Brandberg, Y.; Holm, T.; Glimelius, B.; Martling, A. Radiotherapy regimens for rectal cancer: long-term outcomes and health-related quality of life in the Stockholm III trial. *BJS Open* **2021**, *5*, doi:10.1093/bjsopen/zrab137.

338. Jin, J.; Tang, Y.; Hu, C.; Jiang, L.M.; Jiang, J.; Li, N.; Liu, W.Y.; Chen, S.L.; Li, S.; Lu, N.N.; et al. Multicenter, Randomized, Phase III Trial of Short-Term Radiotherapy Plus Chemotherapy Versus Long-Term Chemoradiotherapy in Locally Advanced Rectal Cancer (STELLAR). *Journal of clinical oncology : official journal of the American Society of Clinical Oncology* **2022**, *40*, 1681-1692, doi:10.1200/jco.21.01667.

339. Liu, W.; Li, Y.; Tang, Y.; Song, Q.; Wang, J.; Li, N.; Chen, S.; Shi, J.; Wang, S.; Li, Y.; et al. Response prediction and risk stratification of patients with rectal cancer after neoadjuvant therapy through an analysis of circulating tumour DNA. *EBioMedicine* **2022**, *78*, 103945, doi:10.1016/j.ebiom.2022.103945.

340. Dijkstra, E.A.; Hospers, G.A.P.; Kranenbarg, E.M.; Fleer, J.; Roodvoets, A.G.H.; Bahadoer, R.R.; Guren, M.G.; Tjalma, J.J.J.; Putter, H.; Crolla, R.; et al. Quality of life and late toxicity after short-course radiotherapy followed by chemotherapy or chemoradiotherapy for locally advanced rectal cancer - The RAPIDO trial. *Radiother Oncol* **2022**, *171*, 69-76, doi:10.1016/j.radonc.2022.04.013.

341. Araujo, R.O.; Vieira, F.M.; Victorino, A.P.; Torres, C.; Martins, I.; Guaraldi, S.; Valadão, M.; Linhares, E.; Ferreira, C.G.; Thuler, L.C. Quality of life in a randomized trial comparing two neoadjuvant regimens for locally advanced rectal cancer-INCAGI004. *Support Care Cancer* **2022**, *30*, 6557-6572, doi:10.1007/s00520-022-07059-6.
